# Molecular dynamics simulations of the conformational plasticity in the active pocket of salt-inducible kinase 2 (SIK2) multi-state binding with bosutinib

**DOI:** 10.1016/j.csbj.2022.05.039

**Published:** 2022-05-23

**Authors:** Mingsong Shi, Lun Wang, Kongjun Liu, Yong Chen, Mengshi Hu, Linyu Yang, Jun He, Lijuan Chen, Dingguo Xu

**Affiliations:** a State Key Laboratory of Biotherapy/Collaborative Innovation Center of Biotherapy and Cancer Center, West China Hospital, Sichuan University, Chengdu, Sichuan 610041, China; b College of Chemistry, MOE Key Laboratory of Green Chemistry and Technology, Sichuan University, Chengdu, Sichuan 610064, China; c Research Center for Material Genome Engineering, Sichuan University, Chengdu, Sichuan 610065, China

**Keywords:** Conformational diversities, Active pocket, Salt-inducible kinase 2, Bosutinib, Molecular dynamics simulation

## Abstract

The kinase domain is highly conserved among protein kinases 'in terms of both sequence and structure. Conformational rearrangements of the kinase domain are affected by the phosphorylation of residues and the binding of kinase inhibitors. Interestingly, the conformational rearrangement of the active pocket plays an important role in kinase activity and can be used to design novel kinase inhibitors. We characterized the conformational plasticity of the active pocket when bosutinib was bound to salt-inducible kinase 2 (SIK2) using homology modeling and molecular dynamics simulations. Ten different initial complex models were constructed using the Morph server, ranging from open to closed conformations of SIK2 binding with bosutinib. Our simulation showed that bosutinib binds SIK2 with up or down conformations of the P-loop and with all the conformations of the activation loop. In addition, the αC-helix conformation was induced by the conformation of the activation loop, and the salt bridge formed only with its open conformation**.** The binding affinity of the models was also determined using the molecular mechanics generalized Born surface area method. Bosutinib was found to form a strong binding model with SIK2 and hydrophobic interactions were the dominant factor. This discovery may help guide the design of novel SIK2 inhibitors.

## Graphical abstract

**Figure:**
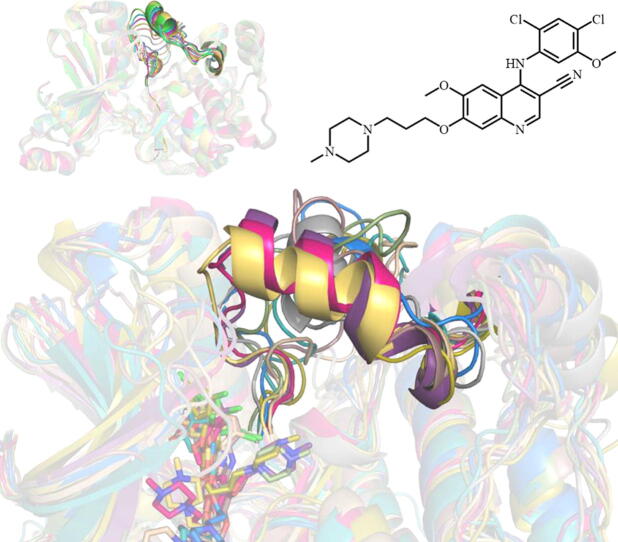

## 1 Introduction

**Fig. 1:**
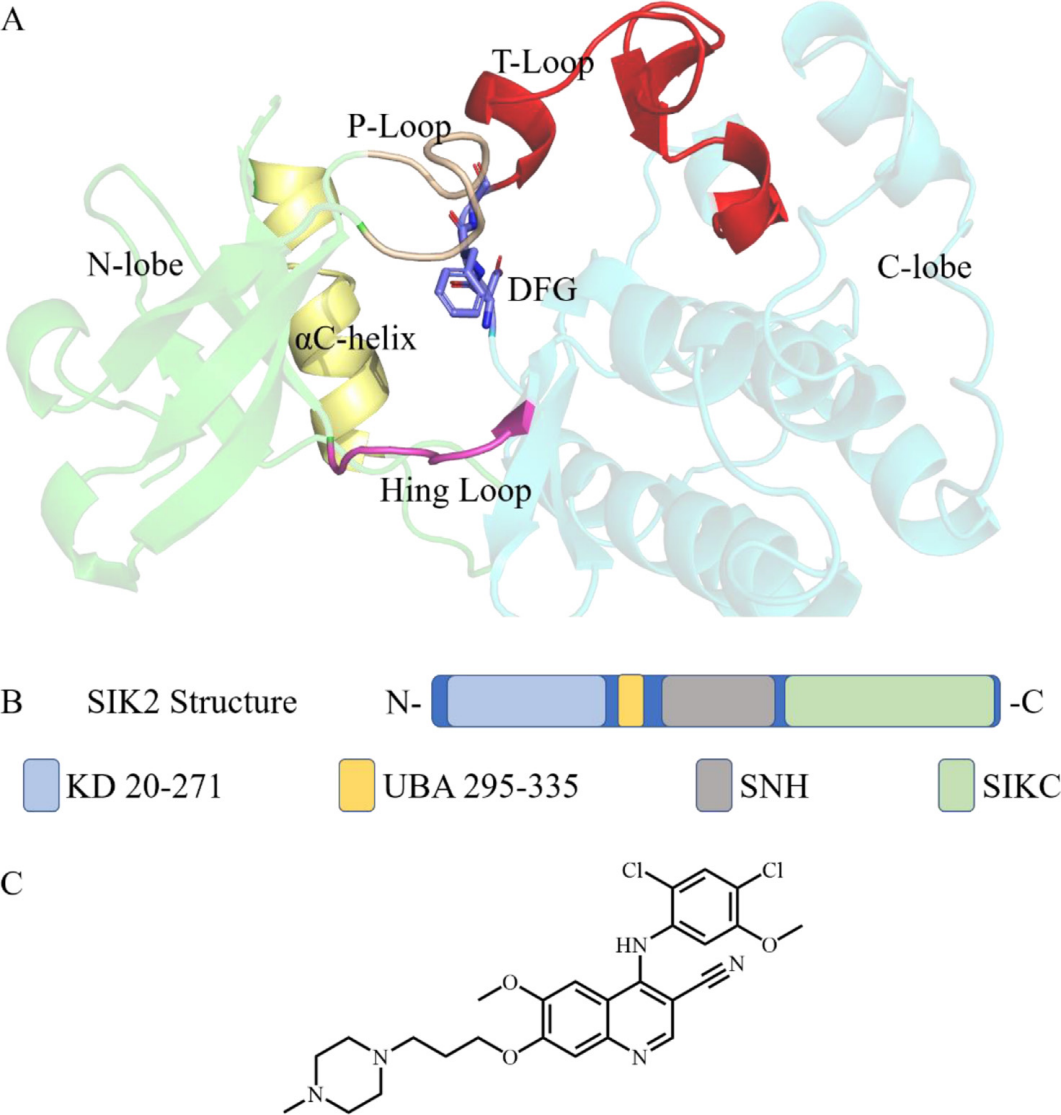
Structure of SIK2 and bosutinib. (A) Structure of the kinase domain. The kinase domain includes an *N*-terminal region (*N*-lobe, green), a C-terminal region (C-lobe, gray), and a hinge loop (magenta). The αC-helix (yellow), DFG motif (marine), *T*-loop (red), and P-loop (wheat) also play important roles in protein kinase activity. The ATP (adenosine triphosphate)-binding pocket (active pocket) was formed between the *N*-lobe and C-lobe. (B) The human SIK2 (UniProt ID: Q9H0K1, residues: 1–926) formed from a kinase domain (K_D_, residues: 20–271), a central SNF protein kinase homology domain (SNH), and a phosphorylation domain near the C-terminal (SIKC). The UBA domain (residues: 295–335) is a linker between K_D_ and SNH. (C) The structure of bosutinib, which is approved as a pan-kinase inhibitor by the Food and Drug Administration (FDA). Bosutinib inhibits SIK2 with an IC_50_ (50 % inhibitory concentration) value of 15 nM in vitro. (For interpretation of the references to color in this figure legend, the reader is referred to the web version of this article.)

The kinase domain structure in protein kinases is highly conserved [1]. The three-dimensional structure of the domain is formed from an *N*-terminal region (*N*-lobe), a C-terminal region (C-lobe), and a linker, referred to as a hinge loop (Fig. 1A) [[1], [2]]. The adenosine triphosphate (ATP)-binding pocket (which is also an activation pocket and active site) is formed between the *N*-lobe and C-lobe. The DFG (Asp-Phe-Gly) motif is highly conserved in the C-lobe among protein kinases and follows the activation loop (also named *T*-loop or A-loop), which serves as an important regulator of kinase activities [[3], [4], [5], [6]]. The salt bridge between a glutamate in the αC-helix and a lysine in the active site regulates protein kinase activation. The conformation rearrangement of the active pocket for most protein kinases can be induced by kinase inhibitors, which can also be utilized for the design of selective inhibitors [[7], [8], [9]].

Conformational rearrangements of the kinase domain are influenced by inhibitor binding, residue phosphorylation, autoinhibition, and amino acid mutations [10]. Generally, for many protein kinases this rearrangement involves the following: (i) movement of the P-loop; (ii) movement of the *T*-loop; and (iii) rotation of the αC-helix in the *N*-lobe. Partial rearrangement of the *T*-loop can be induced by different kinase inhibitors for open and/or closed conformations. For example, dasatinib can bind with the open conformation of the *T*-loop for ABL1 and imatinib in a closed conformation, which results in a higher potential binding affinity than dasatinib (Figure S1) [[11], [12], [13], [14]]. These conformational rearrangements have resulted in conformational diversity of the ATP-binding site when bound to different inhibitors. Thus, the conformation diversities of the active pocket of the kinase domain are important to consider when designing novel kinase inhibitors.

To design a novel selective inhibitor of protein kinase, the conformation plasticity needs to understood. In the present study, we characterized the conformational plasticity of bosutinib binding with salt-inducible kinase 2 (SIK2) as a model system. Although some studies have identified the conformational plasticity of protein kinases [[15], [16], [17], [18], [19], [20], [21], [22], [23]], to our knowledge, there are no related studies for SIK2. SIK2 belongs to the salt-inducible kinase family, which plays an important role in the regulation of cellular growth, metabolism, and apoptosis [[24], [25], [26], [27], [28], [29], [30]]. In addition, SIK2 dysregulation has been recognized in various cancers such as breast, ovarian, lung, and prostate cancer [[31], [32], [33], [34], [35], [36], [37], [38], [39], [40]]. These findings suggest that SIK2 can be applied as a potential target to treat malignancy. The full SIK2 protein consists of a kinase domain (K_D_), a central SNF protein kinase homology domain (SNH), and a phosphorylation domain near the C-terminal (SIKC) (Fig. 1B) [41]. However, in this investigation we have only considered the K_D_ of SIK2. Recent studies have shown the importance of the *T*-loop when certain inhibitors bind with SIK2 [[42], [43]]. From this, we have speculated that the conformations of SIK2 also influence the binding model with different ligands.

Bosutinib was selected as the template inhibitor for SIK2. Bosutinib, which is a Food and Drug Administration (FDA)-approved pan-kinase inhibitor, can inhibit SIK2 from the screening potential SIK2 inhibitors in vitro [[44], [45], [46]]. Bosutinib (Fig. 1C) was approved to treat chronic myeloid leukemia in patients resistant or intolerant to prior lines of therapy as a second-generation pan-kinase inhibitor [47]. Bosutinib inhibits the activity of Bcr-Abl, Src, Yes, Fgr, Fyn, Lck, Lyn, Hck, and Blk [[48], [49]], as well as SIK2 with an IC_50_ (50 % inhibitory concentration) value of 15 nM in vitro [50]. As it exhibits remarkable activity against SIKs and against multi-non receptor tyrosine kinases, bosutinib may be considered a lead compound in the design and/or development of high affinity and selective SIK2 inhibitors.

The crystallization of SIK isoforms has been challenging due to its poor yields in insect cell expression systems and the instability of the protein [51]. This has hindered the development and improvement of SIK2 modulators. Reliable kinase-inhibitor complex structures are clearly vital for structure-based drug development [[52], [53], [54]]. Furthermore, the conformational rearrangement of protein kinases, such as the rearrangement of the active site, can also provide information to enhance the binding affinity and help design selective inhibitors [[17], [18], [19], [20], [21], [22], [23], [55]]. Molecular dynamics (MD) simulations have proven to be very valuable and are widely used to study the conformation rearrangements of proteins and the binding models for inhibitors with targets [[56], [57], [58]].

In the present study, bosutinib-bound conformation diversities for the ATP-binding pocket of SIK2 were studied using homology modeling (HM) and MD simulations. Different initial conformations of the *T*-loop, constructed from the Morph server, were used to study the diversities of the activation pocket when bosutinib binds with SIK2. The binding free energies were also calculated from the post-analysis of the simulation using the molecular mechanics generalized Born surface area (MM/GBSA) method. We believe that this study will provide useful information to help improve our understanding of the conformational diversities of the active pocket of SIK2 bound with bosutinib.

## 2 Methods

### 2.1 Homology modeling

To construct SIK2 structures with open and closed conformations of the *T*-loop, two separate modeling experiments were carried out using the online service SWISS-MODEL [59]. Herein, only the kinase domain and UBA domain (residues: 1–400) sequences were selected for further structural construction. The atomic coordinates of the crystal structure of MARK2 (PDB ID: 2WZJ [60]), derived from *Homo sapiens*, were used to construct the closed conformation of the *T*-loop for SIK2. The structure of SIK2, with an open *T*-loop conformation, was also modeled using the available crystal structure for MARK2 (PDB ID: 3IEC [61]) from *Homo sapiens*. Both SIK2 and MARK2 belong to the calcium/calmodulin-dependent protein kinase (CAMK)-like family, indicating that these proteins have similar structures and functions [[62], [63]]. ERRAT [64] and VERIFY3D [65] as well as ProCheck [66] software were used to check the quality of the two models.

### 2.2 Molecular dynamics simulations

The complex structures of bosutinib binding with SIK2 were obtained by aligning the structures identified for SIK2 to the bosutinib/Src complex (PDB ID: 4MXO [67]). First, based on the superposition with the protein, the SIK2 structure moved to the bosutinib/Src complex. Second, the coordinates of bosutinib were extracted from the bosutinib/Src complex structure. Therefore, the bosutinib coordinates were fitted into the ATP-binding site of SIK2, thus bosutinib/SIK2 complex systems were constructed. Molecular dynamics simulations were then run for the two complex bosutinib/SIK2 systems. The force field parameters for bosutinib were generated using the restrained electrostatic potential (RESP) protocol [68] and the general amber force field (GAFF, version 2) [69] with the Antechamber module in AMBERTools21 [70]. The AMBER ff19SB force field [71] was used to create the topology parameters of SIK2.

The systems were neutralized using sodium chloride ions, and solvated with TIP3P water [72] in a 15 Å cuboid box. Finally, the system included 318 residues of SIK2, one small molecule of bosutinib, and approximately 15 000–20 000 solvent water molecules. The system was subjected to 9000 steps of the steepest descent method and then a 1000-step conjugate gradient, while fixing all the solute molecules at their initial positions. Then, the 10,000-step conjugate gradient method was used to optimize the entire system. After these steps, the overall system temperature was heated from 0 K to 300 K in 200 ps using Langevin dynamics. The pressure was then maintained at 1 bar for 200 ps with isotropic position scaling. Subsequently, the system was equilibrated at 300 K and 1 bar within the NPT ensemble at 200 ps. Subsequently, the entire system underwent MD simulations for final data collection and analyses. All simulations were performed using AMBER20 [70]. The *CPPTRAJ* module [[73], [74]] was then used to analyze the data from the MD trajectories. Detailed information regarding the molecular dynamics simulations and cluster analysis are provided in the Supporting Information.

### 2.3 Interpolation of structural changes

Representative structures of the open and closed conformation complex systems were obtained from the cluster analyses from the last 100 ns MD simulations. The two structures were also analyzed using the Morph server [75]. The server produces 2D and 3D animations of a plausible or semi-plausible pathway between closed and open bosutinib/SIK2 conformations and uses energy minimization to calculate the intermediate frames. The motion is placed in a standardized coordinate system so that the statistics derived from any two motions will be directly comparable. Instead, several key standardized statistics such as maximum Cα displacement and the differences in the torsion angles between the starting and final structures were chosen to show the motions of each amino acid through the activation process. The resulting eight intermediate structures were used to represent the interpolated motions of the kinase domain through the translation process. For this purpose, these complex structures with intermediate, closed, and open conformations of the *T*-loop were subjected to MD simulations. We ran a 500 ns simulation for every bosutinib/SIK2 complex system and a total simulation of 5000 ns.

### 2.4 Binding free energy

The MM/GBSA approach [[76], [77]] was employed to calculate the binding free energies between the inhibitor bosutinib and SIK2. It is an efficient method that can be used to improve our ability to evaluate ligands and enzyme systems [[78], [79], [80]]. The MM/GBSA framework has previously been extensively discussed [[81], [82], [83]]. For each complex system, we used the MM/GBSA method to obtain energy terms via a statistical average from the last 200 ns of the MD trajectory over 1000 frames. The entropy was averaged over an interval of 2 ns with 100 frames. To gain insight into the contributions of each residue, the total binding energy between SIK2 and the inhibitor bosutinib was decomposed with the MM/GBSA binding energy decomposition, without consideration of the entropies [84]. The energy was calculated using the MMPBA.py program in AMBERTools21 [85]. More information about the binding free energy calculation is provided in the Supporting Information. A scheme of the methods is shown in Scheme S1.

## 3 Results and discussion

### 3.1 Modelling of open and closed conformations

A Protein Data Bank (PBD) survey revealed that ≥ 13 kinase crystal structures formed complexes with bosutinib (Table S1) [[67], [86], [87], [88], [89], [90], [91], [92], [93]]. All structures demonstrated similar binding patterns, as they all had highly conserved three-dimensional structures among the protein kinases (Figure S2). Bosutinib (ligand ID: DB8) showed extremely similar geometric conformations with the protein kinase (Figure S2). However, the conformations of the *T*-loop were diverse from those of the qualitative analyses, such as 3SOA (reference to PDB ID) [86], 3UE4 [87], 4MXO [67], 4MXX [67], 4MXY [67], 4MXZ [67], 4QMN [88], 5VC3 [91], 5VCY [91], and 6FDY [92] with open conformations which moved toward the solvate environment and far away from the ATP-binding pocket, 5AJQ [89] with closed conformation whose *T*-loop moved toward the ATP-binding pocket, 5I9X [90] and 6OP9 [93] with intermediate states between the open and closed conformations of the *T*-loop (Figure S3). Thus, bosutinib can bind to the diverse protein kinase *T*-loop conformations. Consequently, knowledge of the open, intermediate, and closed conformations is required to explore the binding mechanism of SIK2 to bosutinib.

**Fig. 2:**
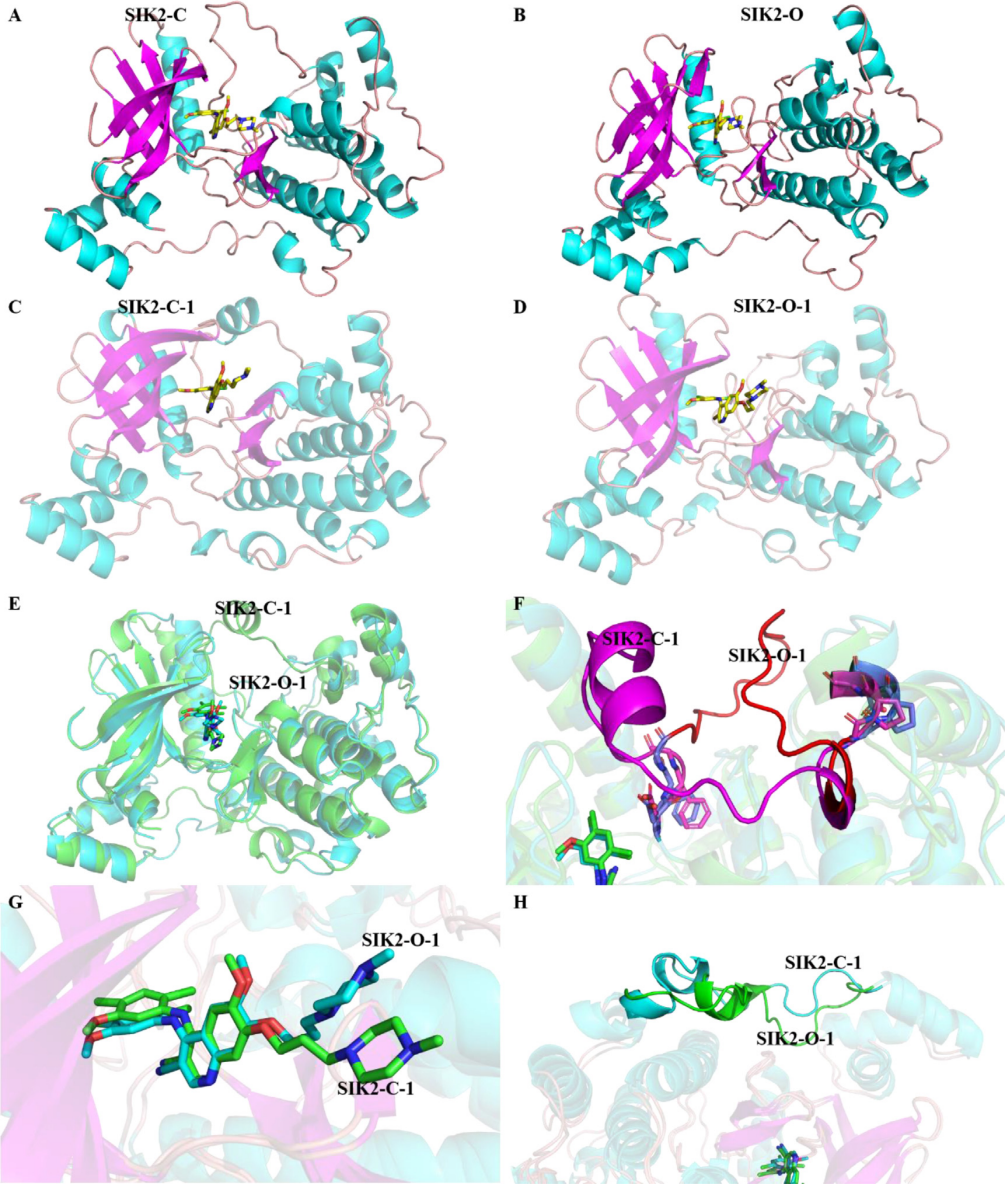
Structures of the SIK2 with open or closed conformations of the *T*-loop. (A) Constructed structure (SIK2-C) with a closed conformation of the *T*-loop from homology modeling based on MARK2 (PDB ID: 2WZJ). (B) Constructed structure (SIK2-O) with open conformations of the *T*-loop from homology modeling based on MARK2 (PDB ID: 3IEC). The center frame of the first cluster defined as the representative structures for (C) the bosutinib/SIK2-O system (SIK2-O-1) and (D) the bosutinib/SIK2-C system (SIK2-C-1), which were determined from the cluster analysis of the last 100 ns of the molecular dynamics simulations. (E) Superposition bosutinib/SIK2-O-1 to bosutinib/SIK2-C-1. (F) The enlarged *T*-loop conformation part of (E). (G) The enlarged ligand molecule of (E). (H) The enlarged linker between the UBA domain and the kinase domain of (E). SIK2 is shown with the cartoon and bosutinib with the sticks.

In this study, we constructed a closed and open conformation of the *T*-loop for SIK2 using homology modeling. First, the closed and open conformations of the *T*-loop of human SIK2 structures were modeled based on the available crystal structures of the MARK family protein. There were four structures with no missing residues in the *T*-loop from the Protein Database Bank (Table S2). The 6C9D [94] and 3FE3 [95] structures have been published for MARK1 and MARK3, respectively. As both 3IEC and 2WZJ are for MARK2, we utilized these two crystal structures as template structures. To construct the closed conformation structure (known as SIK2-C, Fig. 2A), the experimentally determined structures with a closed conformation of MARK2 (PDB ID: 2WZJ [60]) were used as the template (Figure S4). While the open conformation of the *T*-loop for SIK2 (labeled as SIK2-O, Fig. 2B) was constructed using MARK2 (PDB ID: 3IEC [61]) as the template (Figure S5). The least square fitting of the modeled structures with the original crystal templates indicates minor conformational differences in the term root mean square deviation (RMSD) of the backbone (0.10 Å for the SIK2-C model to 2WZJ structure and 0.23 Å for the SIK2-O model to 3IEC structure). The modeled structures were evaluated to determine whether they were equally reliable as the initial template structures. The overall quality factor for the non-bonded atomic interactions was greater than 84 % with the ERRAT analysis (Figure S6). Meanwhile, the analysis results from VERIFY-3D revealed that more than 78.0 % of the residues had an average 3D-1D score greater than 0.2 (Figure S7). The Ramachandran plot analysis of the models showed that less than 0.01 % of the residues were within the disallowed regions and that greater than 89.0 % were in the most favored regions (Figure S8). These results indicate that the constructed SIK2 structures were of high quality and suitable for subsequent studies. In addition, the main difference between SIK2-O and SIK2-C were in the conformation of the *T*-loop (Figure S9). Therefore, the constructed protein structures for SIK2 were used as receptor proteins in the subsequent simulations.

After model building, the initial conformations of the pan-inhibitor bosutinib in the ATP-binding pocket of SIK2 were obtained using three steps. First, we superimposed SIK2-C (or SIK2-O) to the bosutinib/Src complex (PDB ID: 4MXO [67]). The coordinate of SIK2-C (or SIK2-O) were moved to the coordinates of bosutinib/Src. Second, the coordinates of bosutinib were extracted from the bosutinib/Src complex and the coordinates of the superimposed SIK2-C (or SIK2-O) were saved. Third, coordinates of the bosutinib and SIK2-C (or SIK2-O) were combined within the same complex. When bosutinib binds to different kinases, a similar conformation of bosutinib has been found from the RMSD of bosutinib less than 1.2 Å (Figure S10). Therefore, the bosutinib molecule from the bosutinib/Src complex was applied to obtain two complex systems with bosutinib/SIK2-C and bosutinib/SIK2-O (Figure S11). To obtain more accurate complexes, 200 ns MD simulations were employed for the bosutinib/SIK2-O and bosutinib/SIK2-C systems.

The overall stability and structural relaxation of the bosutinib/SIK2-C and bosutinib/SIK2-O were monitored by computing the time evolution of the RMSD of the backbone atoms during the simulations (Figure S12). The backbone RMSD (2.78 ± 0.20 and 2.19 ± 0.26 Å for SIK2-C and SIK2-O, respectively) as a function of time was relatively stable after approximately 50 ns of the simulation. However, the value of the RMSD of bosutinib (2.42 ± 0.72 and 2.51 ± 0.72 Å for SIK2-C and SIK2-O, respectively) meant that the ligand could fluctuate in the active pocket of the SIK2. The stability of the complex systems was also determined from the gyration radius (RADGYR) (Figure S13) and the surface area of the complex (Figure S14). However, the *T*-loop (^160^DFG-APE^186^) of the bosutinib/SIK2-C system appeared more flexible than bosutinib/SIK2-O with an RMSF of more than 2.0 Å (Figure S15), which is in agreement with the other inhibitors bound with SIK2.[43] This indicates that bosutinib can induce greater rearrangement of the *T*-loop for SIK2-C than for SIK2-O. To check the complex in the simulation times, the structures initially and at 50, 100, 150, and 200 ns were also extracted from the MD simulation (Figures S16-S17). The structure of the protein shown little fluctuation from the initial structure. These results indicate that the complex structures were stable, and the inhibitor was strongly bound to SIK2. Overall, these results confirmed that SIK2-O and SIK2-C could form stable complexes with bosutinib in the ATP-binding site of SIK2.

### 3.2 Modelling the transition state

Cluster analysis was applied to find representative structures for the bosutinib binding with SIK2-O and SIK2-C from the 200 ns simulations. Ten thousand frames from the final 100 ns simulation were grouped into five clusters for the bosutinib/SIK2-C and bosutinib/SIK2-O systems (Figures S18–S19). The representative frames (centroid frame in the cluster) of the highest occupancy clusters for the SIK2-C (46.9 %) and SIK2-O (33.8 %) systems were labeled as SIK2-C-1 and SIK2-O-1, respectively (Fig. 2). Meanwhile, the RMSD values for the five centroid frames of each cluster were small enough at 1.82 Å and 2.03 Å for SIK2-C and SIK2-O, respectively. The smaller number of the RMSDs also indicates that the conformations were similar. Simultaneously, a similarity in the structure of the five centroid frames for the SIK2-O system can be found. Therefore, SIK2-C-1 is representative of the closed conformation of SIK2 and the open conformation of SIK2-O-1. In summary, SIK2-C-1 and SIK2-O-1 were selected as representatives of the closed and open conformations of SIK2, respectively.

**Fig. 3:**
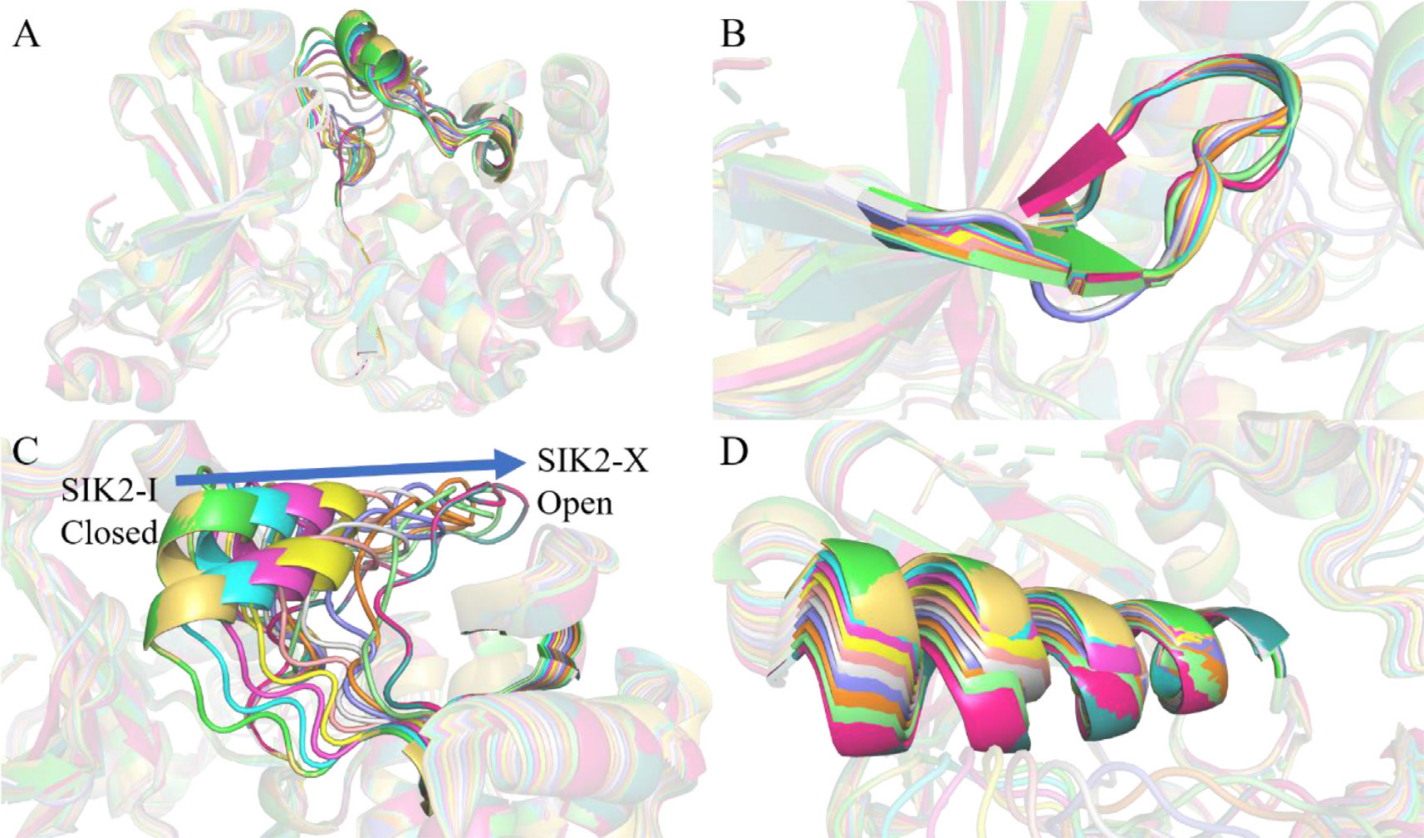
Ten structures had closed, intermediate, and open conformations of the *T*-loop for SIK2. (A) for the overall structures; (B) for the enlarged conformations of the P-loop; (C) for the enlarged conformations of the *T*-loop; and (D) for the enlarged conformations of the αC helix.

Least square fitting of the SIK2-C-1 and SIK2-O-1 conformers indicated that major structural changes were associated with the *T*-loop (Fig. 2). The overall structures and ligand molecules were similar between SIK2-C-1 and SIK2-O-1. To provide a more representative understanding of the conformational motions of the *T*-loop, the closed structure was morphed into an open structure using the Yale Morph Server [75]. There were eight intermediate states between SIK2-C-1 and SIK2-O-1 obtained from the modeling. The 10 different conformations were labeled as SIK2-I (SIK2-C-1), SIK2-II, SIK2-III, SIK2-IV, SIK2-V, SIK2-VI, SIK2-VII, SIK2-VIII, SIK2-IX, and SIK2-X (SIK2-O-1) (Figures 3 and S20). The entire structure for the ten constructed SIK2 structures has a greater RMSD value, especially for the open or closed conformations. The differences among the ten structures mainly come from the *T*-loop, P-loop, and αC-helix changes. Therefore, these ten conformations of SIK2 can be seen as the representative structures from the open to closed conformations of the *T*-loop. To obtain more information regarding the conformation plasticity of the active site for bosutinib bound with SIK2, these complex systems were subjected to 500 ns MD simulations for every system.

### 3.3 System stability

The RMSD of the backbone atoms in SIK2 were plotted for the ten bosutinib/SIK2 systems (Figure S21). The RMSD protein value was calculated to be 1.92 ± 0.32 Å for the bosutinib/SIK2-I system throughout the 500 ns MD simulation. A small fluctuation indicates the stability of the whole system, and the overall structure of the protein has less chance from the initial structure. Meanwhile, a larger RMSD of the ligand (4.53 ± 1.04 Å) than the protein indicates that bosutinib fluctuates in the active pocket of SIK2. However, the ligand was found to stay in the pocket based on the extracted frames from the MD simulation for bosutinib/SIK2-I (Figures S22). A At the same time, the conformation of the bosutinib can fluctuate in the active pocket, for example, the 2,4-dichloro-5-methosyphenyl group changed their conformation and maintained their hydrophobic interactions with SIK2. The piperazine group changed with a larger movement in the solvent environment (Figure S23). Moreover, the piperazine group of bosutinib translated within the solvate, which resulted in the fluctuation of bosutinib for bosutinib/SIK2-III among the 240–460 ns simulation times (Figure S24). Furthermore, the RMSD value of the bosutinib/SIK2-V system also shows that bosutinib is flexible in the pocket from 300 to 500 ns. The bosutinib moves out of the hydrophobic region at the bottom of the active site and back into the pocket after 430 ns (Figure S25). This is not induced by the *T*-loop conformation. Meanwhile, both the protein and bosutinib molecules were stable in the simulations for the other bosutinib/SIK2 systems such as 1.99 ± 0.28 Å and 2.66 ± 1.10 Å for the protein and ligand of SIK2-II, respectively. The initial complex structure and the 500th ns frame were extracted to find that the inhibitor was stable in the pocket of the SIK2, except for the bosutinib/SIK2-I system (Figure S26). In summary, all ten modeling systems were stable after 100 ns and thus suitable for use in subsequent analyses. Meanwhile, the suspicious gap along the reaction coordinates between the open and closed states was checked using the RMSD value of the kinase domain within the 500 ns MD simulation for bosutinib/SIK2 systems referenced to the initial conformation for SIK2-I (Figure S27). It indicated that there is no suspicious gap along the reaction coordinates from the open to closed states of the *T*-loop.

The root-mean-square fluctuation (RMSF) values were further analyzed from the 500 ns MD trajectory. Interestingly, four regions of the sequence showed large fluctuations (RMSF larger 2.00 Å; Figure S28), that is, the regions at positions 26–32 (P-loop), 160–180 (*T*-loop), 217–240 (the K_D_ terminal), and 275–295 (linker between kinase domain and UBA domain) of SIK2 in the SIK2-I system. The same situation also occurred in the other nine models. The details of the fluctuating region are discussed in the following sections. However, this can also be observed from the snapshots taken 100, 200, 300, 400, and 500 ns from the MD trajectory (Figures S22, and S29-S37). In general, among the simulated models, the relative position of the 4-methylpiperazine group on the ligand showed fluctuations, except in the bosutinib/SIK2-I and bosutinib/SIK2-II systems, which agreed with previous RMSD and RMSF analyses. In our previous study for dasatinib/SIK2, it was found that the piperazine group had similar fluctuations to the 4-methylpiperazine group of bosutinib (Figure S38) [43]. The piperazine of the dasatinib, which is similar to the 4-methylpiperazine group of bosutinib, also showed fluctuations. This piperazine region was a surface-exposed portion of the inhibitors [[11], [12], [96]]. However, the fragment in the solvent environment can be used to improve the bioavailability [[97], [98]]. In summary, the SIK2 conformation should be stable when bosutinib is bound to the ATP-binding site.

**Fig. 4:**
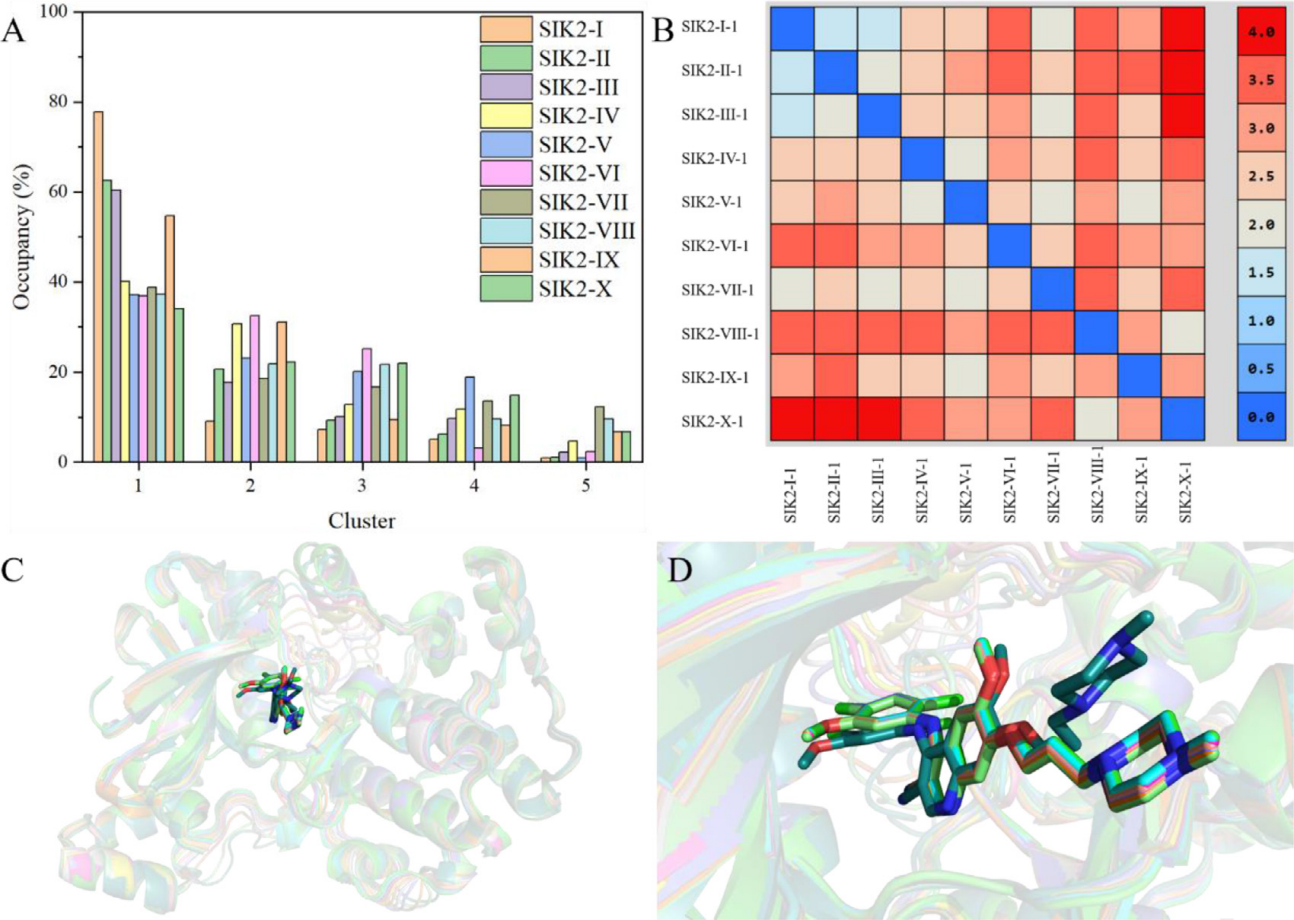
Cluster results for the ten bosutinib/SIK2 complex systems from the last 200 ns MD simulations. (A) Percentage frame number for the five clusters in the total 10 000 frame from the final 200 ns simulations for every bosutinib/SIK2 system. (B) The RMSD values among the ten representative frames. The representative frames were labeled as SIK2-I-1, SIK2-II-1, SIK2-III-1, SIK2-IV-1, SIK2-V-1, SIK2-VI-1, SIK2-VII-1, SIK2-VIII-1, SIK2-IX-1, and SIK2-X-1. (C) The aligned representative frames of cluster 1 from the ten bosutinib/SIK2 systems. (D) Bosutinib conformation of the representative frame for cluster 1 from the ten bosutinib/SIK2 systems.

**Fig. 5:**
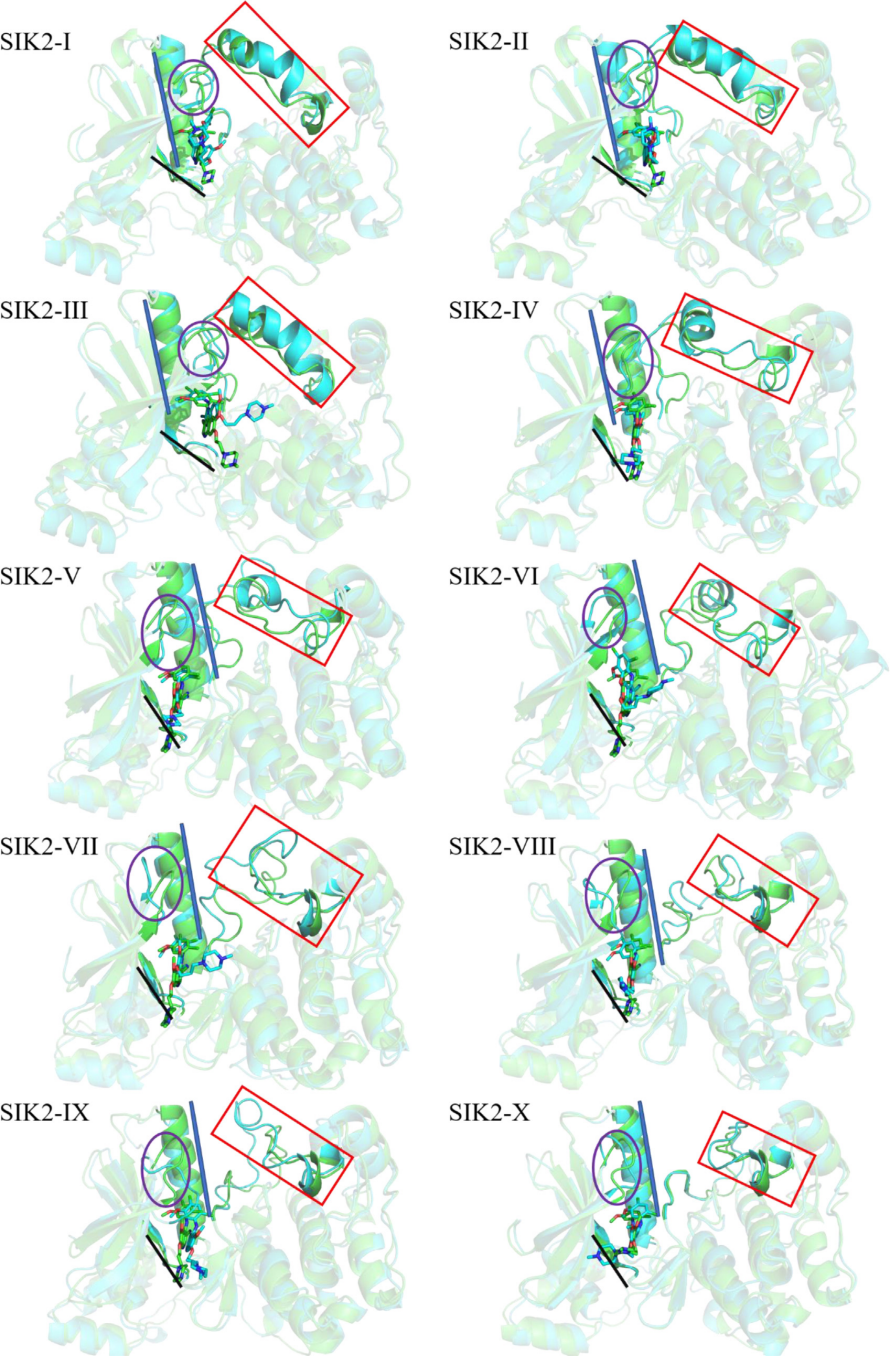
Representative conformations for the bosutinib/SIK2 complex systems and their comparisons with the initial structures. The representative conformations for every bosutinib/SIK2 system were obtained from the centroid of the first cluster using cluster analysis (green color). The initial conformation of every bosutinib/SIK2 system was obtained from the Yale Morph Server results (cyan color). The SIK2 protein is shown with the cartoon and bosutinib with the sticks. The red rectangle represents the *T*-loop region, the purple circle the P-loop region, the black line the hinge loop region, and he blue cylinder the αC helix. (For interpretation of the references to color in this figure legend, the reader is referred to the web version of this article.)

Clustering analysis for all conformations was also performed during the MD simulation to identify structurally similar conformations for each bosutinib/SIK2 system. The centroid frames of the sampled cluster of the bosutinib/SIK2 are shown in Fig. 4. Cluster 1 represented the predominant cluster and was populated around 77.8 % of the total frames for SIK2-I. Cluster 1 accounts for 62.6 %, 60.4 %, 40.1 %, 37.1 %, 36.9 %, 38.8 %, 37.3 %, 54.7 %, and 34.1 % of the SIK2-II, SIK2-III, SIK2-IV, SIK2-V, SIK2-VI, SIK2-VII, SIK2-VIII, SIK2-IX, and SIK2-X, respectively. Meanwhile, we found that the five representative frames were highly similar among the same complex systems from the RMSD value (Figure S39) and the overlay of those frames (Figures S40-S49). Therefore, the centroid structure of cluster 1 for every bosutinib/SIK2 system was selected as the representative conformation (Fig. 5).

### 3.4 P-loop conformation

From the RMSF analysis, we found that the P-loop was flexible during the simulations (Figure S28). To evaluate the fluctuations in the simulation, the RMSD value of the P-loop (^26^LGKGN^30^) was calculated for the ten bosutinib/SIK2 systems (Figure S50). For the SIK2-I and SIK2-X systems, the conformation of the P-loop can be maintained in the last 200 ns, and the others can fluctuate in the 500 MD simulation times. Specifically, the P-loop in bosutinib/SIK2-IX fluctuated between 0.59 Å and 10.23 Å and bosutinib/SIK2-II between 0.38 Å and 6.87 Å. However, as the hinge loop conformation remains rigid in the simulations (Figure S51), the distance for the coordinate center of the selected residues between ^27^GKG^29^ in the P-loop and the ^97^EYA^99^ in the hinge loop were applied to investigate the changes (Figure S52). The distance also indicates that the P-loop can form up or down conformations for all systems. Interestingly, the distance between the N30 in the P-loop and K49 in the bottom of the active pocket was calculated to determine the flexibility of the P-loop in the MD simulation. The distances for N30:CA_K40:CA, N30:ND2_K49:NZ, and N30:OD1_K49:NZ were calculated for the ten systems (atom labels are shown in Figure S53). The distance between the side chains of N30 and K49 was more flexible than the distance between the backbone of bosutinib/SIK2-I (Figure S54). Although the backbone distance was maintained in the last 200 ns, the sidechain distance could not be maintained in the entire simulation time for bosutinib/SIK2-II (Figure S55). A similar trend was observed for the other eight complex systems (Figure S56). They may result from the side chain being flexible in the simulation time, which was also found for the other residues, and the side chain of K49 with five heavy atoms was longer than that with the other residues. This means that the conformation of the P-loop could be a rearrangement with the conformations of the *T*-loop. The rearrangement is clearer in the intermediate state than in the closed or opened conformations of the *T*-loop.

The P-loop can form a down or up conformation when HG-9–91-01, KIN112, MRT199665, and MRT67307 bind with SIK2 [42]. Additionally, the P-loop also forms a down conformation when bosutinib binds to CAMKII [86] and STK10 [89] (Figure S57). Meanwhile, the up conformation of the P-loop was also found for bosutinib with Src [67], STK24 [88], EPHA2 [90], Wee1 [91], and HER3 [93] (Figure S58). Excluding the same inhibitor could induce up or down conformations of the P-loop for different protein kinases, the same protein kinases could also be induced by different inhibitors to form the up or down conformation of the P-loop; for example, MARK2 forms an up conformation with N-((1S,2R)-2-aminocyclohexyl)-4-(6-(1-methyl-1H-pyrazol-4-yl)pyrazolo[1,5-*a*]pyrimidin-3-yl)thiophene-2-carboxamide (PDB ID: 5EAK [99]) and down conformation with (S)-7-(1-(4-fluorophenyl)ethyl)-5,5-dimethyl-2-(pyridin-3-ylamino)-5,7-dihydro-6H-pyrrolo[2,3-*d*]pyrimidin-6-one (PDB ID: 5KZ7 [100]) (Figure S59). Our analyses indicate that bosutinib can bind with SIK2 when the P-loop is in either the up or down conformation.

### 3.5 *T*-loop conformation

The *T*-loop is a long flexible loop that shows high structural variation among protein kinases. In most protein kinases, the phosphorylation of the *T*-loop plays a key role in kinase activation; for example, the phosphorylation the *T*-loop of SIK2 can activate an interaction with the 14–3-3 protein [[101], [102]]. From our previous work on dasatinib, KIN112, HG-9–91-01, MRT199665, and MRT67307 binding with SIK2, it was found that the *T*-loop can form closed and opened conformations.[[42], [43]] Relatively flexible *T*-loop conformations were also found for bosutinib/SIK2 from the previous RMSF analysis (Figure S28). Meanwhile, the *T*-loop maintained a similar fluctuation from the different initial conformations. The residues of the *T*-loop for most of the published crystal structures of the MARK family proteins are missing (Table S2) [[99], [100], [103], [104], [105], [106], [107]]. Furthermore, different conformations of the *T*-loop were also found for other kinases; for instance, the *T*-loops of the RIPK family members can recognize various inhibitors with opened or closed conformations [108]. Therefore, we identified the conformations for bosutinib binding with a different initial conformation of the *T*-loop of SIK2.

**Fig. 6:**
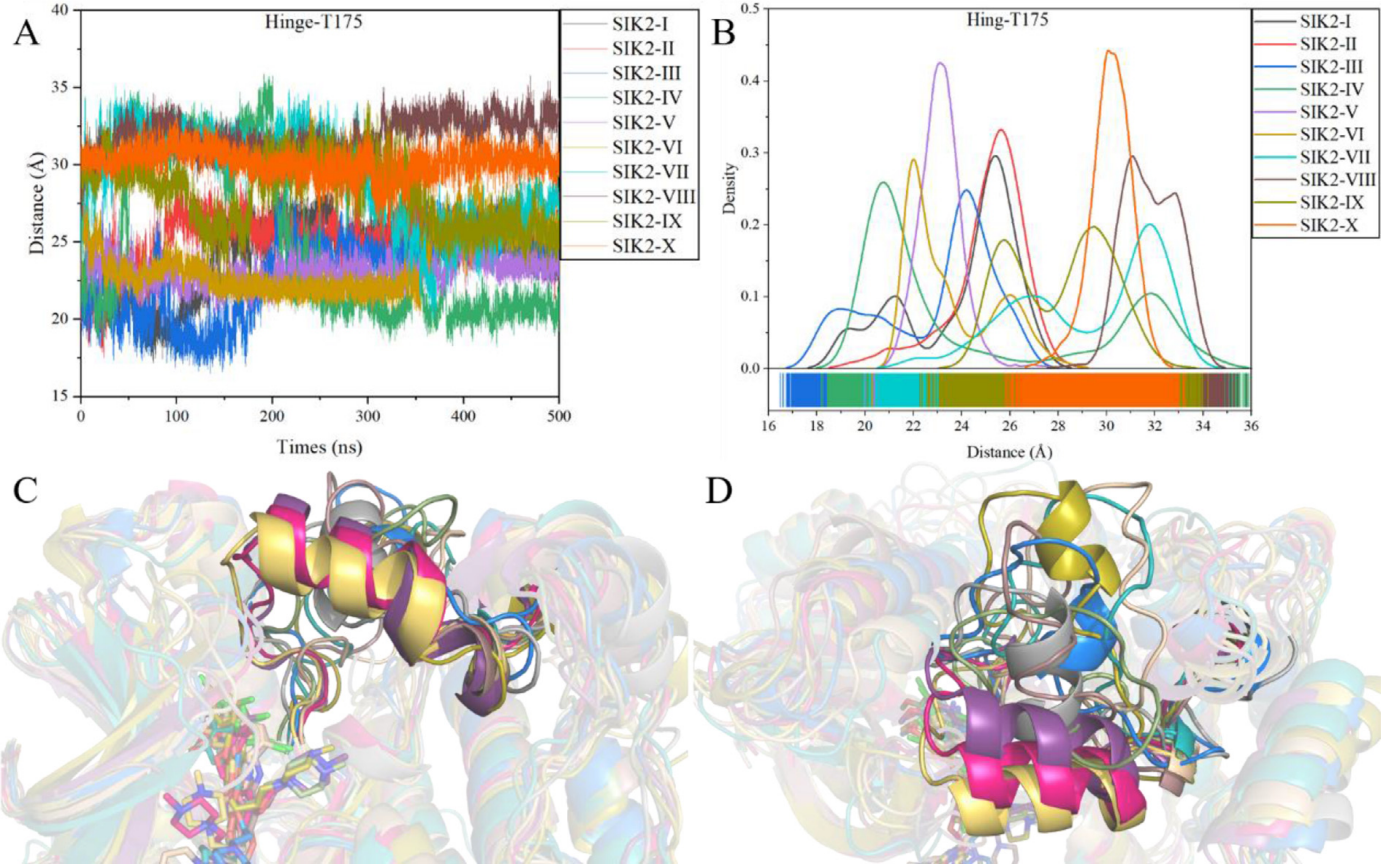
Conformation of the *T*-loop for the ten bosutinib/SIK2 complex systems. (A) For the distance between the center of the hinge and the T175 (*T*-loop) vs the simulation times; (B) for the density of the distance between the center of the hinge and the T175 (*T*-loop) among the simulation times; (C) for the *T*-loop conformations of the ten systems with the representative frames; (D) for the translation view with (C).

Here, the opened and closed conformations of bosutinib/SIK2 were identified. To obtain detailed information about the stability of the subsegments in the bosutinib/SIK2 systems, the RMSD value of the *T*-loop was analyzed (Figure S60). The *T*-loops had higher average RMSD values than the overall protein (Table S3). The *T*-loop plays an important role in the binding of the ATP-binding pocket with ATP or inhibitors. Therefore, their higher average RMSD values and more dynamic behavior indicate that the *T*-loop is more flexible in its open conformations. The *T*-loop can be maintained in the bosutinib/SIK2-I, III, V, VI, and VIII systems, which means that the *T*-loop may maintain one conformation in the last 200 ns simulation (Figure S61). Meanwhile, the *T*-loop conformation was retained in the last 200 ns for bosutinib/SIK2-II, IV, and X, which were also found in the structures (Figure S62). For bosutinib/SIK2-VII, the *T*-loop fluctuated in the overall simulation time, which was also verified from the extracted frames (Figure S63). However, the RMSD of the *T*-loop for SIK2-IX increased in the last 200 ns and agreed with the extracted frames (Figure S64). We also calculated the distance between the hinge loop and the *T*-loop, which indicates the similarity in the results for these complex systems (Figures 6 and S65). For example, the distance between the hinge loop and the T175 of the *T*-loop can be maintained in the last 200 ns, excluding SIK2-VII and IX, which agrees with the RMSD analysis. In summary, the *T*-loop maintains its own conformation based on the initial conformation. In other words, the ten initial conformations need to be considered to find novel inhibitors that target SIK2 when using structure-based drug discovery.

The conformation of the *T*-loop (open conformation) is similar among the protein kinases in the active state. However, the conformation of the *T*-loop for the inactive state of the protein kinase or the kinase with different inhibitors is diverse and flexible [[15], [16], [109]]. In particular, the conformation of the *T*-loop for ABL1 can be open or closed with different inhibitors, such as the closed conformation with imatinib (PDB ID: 2HYY [13]) and open conformation with axitinib (PDB ID: 4TWP [110]) (Figure S66). This flexible *T*-loop also plays an important role in inhibitor recognition. For example, dasatinib can bind to both conformations of the *T*-loop [[79], [111], [112]] and shows more potential ABL1 activity than the imatinib bound in only the closed conformation [[13], [113], [114]]. In this study, open, closed, and intermediate conformations of the *T*-loop were constructed as initial models to consider the bosutinib-binding conformation. In summary, bosutinib was found to bind to all the constructed conformations of the *T*-loop. The conformation of the *T*-loop was plasticity when bosutinib bound into the active pocket of SIK2 with the high binding affinity. Meanwhile, the different conformations may have different binding affinities, which will be discussed in the following section.

### 3.6 αC-helix conformation

The αC-helix also has a mutual regulatory effect on the *T*-loop position. In contrast, the *T*-loop can also regulate the αC-helix position. This regulatory effect is a key mechanism in determining the activity of protein kinases. The residues of the αC-helix show less fluctuating behavior and the linker between the αC-helix and β3 (L1, labeled in Figure S67) showed more fluctuations, especially with bosutinib/SIK2-IX (Figure S67). This linker is located at the *N*-terminus of the αC helix. This means that the position of the αC-helix changed with the fluctuations of the linker. Meanwhile, the linker loop between the αC-helix and β4 (L2, labeled in Figure S67) showed little flexibility in the MD simulations. However, the αC-helix can maintain the conformation in the simulations, which can also be found from the RMSD value (Figure S68).

**Fig. 7:**
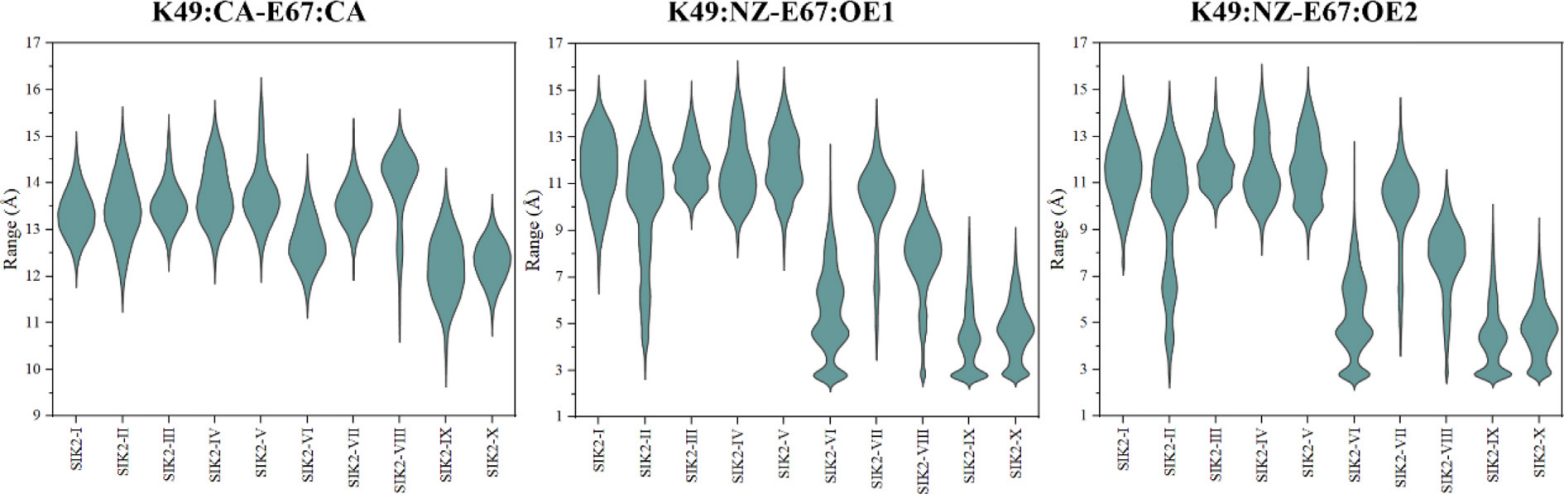
Salt bridge (K49-E67) between the αC helix and the *N*-lobe for the bosutinib/SIK2 complex systems. D_K49:NZ-E67:OE1_ is representative of the distance between the NZ atom of K49 and the OE1 atom of E67, D_K49:NZ-E67:OE2_ the distance between the NZ atom of K49 and the OE2 atom of E67, and D_K49:CA-E67:CA_ the distance between the CA atom of K49 and the CA atom of E67.

As the salt bridge between K49 and E67 plays an important role in kinase activation of SIK2, the distance between K49 and E67 was calculated in the simulation times. The distance can be checked using three different methods: (1) the distance between the NZ atom of K49 and the OE1 atom of E67 (D_K49:NZ-E67:OE1_), (2) the distance between the NZ atom of K49 and the OE2 atom of E67 (D_K49:NZ-E67:OE2_), and (3) the distance between the CA atom of K49 and the CA atom of E67 (D_K49:CA-E67:CA_) (Figure S69). The D_K49:CA-E67:CA_ is approximately 13.5 Å in the bosutinib/SIK2 complex systems excluding SIK2-VI, SIK2-IX, and SIK2-X (Table S4). The distance was found to be well maintained in all ten systems (Figure S70). SIK2-VI, with an initial intermediate conformation of the *T*-loop, can decrease the distance to 12.73 Å. However, the other intermediate conformations of the *T*-loop systems did not exhibit the same tendency. Meanwhile, the open conformation of the *T*-loop can induce the αC-helix towards the ATP pocket. The open or closed conformation of the *T*-loop can also induce the corrected conformation of the αC-helix from the positive value of the cross correlation between the *T*-loop (Residues: 160–180) and αC-helix (Residues: 58–73) although the simulation time (Figure S71). At the same time, the D_K49:NZ-E67:OE1_ and D_K49:NZ-E67:OE2_ for the bosutinib/SIK2 systems were larger than 7 Å, excluding SIK2-VI, SIK2-IX, and SIK2-X, which were larger than 4 Å (Fig. 7). Therefore, a salt bridge can be formed in the open conformation of the *T*-loop and subsequently disordered for closed conformation. In addition, dynamic cross-correlation maps of the Cα–Cα displacement were applied to understand the effects of bosutinib on the conformational changes in the SIK2 (Figure S72). This indicates that pairs of residues in the *T*-loop regions and the αC helix region have been moving in the opposite direction. In summary, the αC-helix conformation was induced by the conformation of the *T*-loop, and the salt bridge formed only with the open conformation of the *T*-loop.

### 3.7 Binding free energy

While binding models obtained from the MD simulations could provide some useful information about the conformation of the *T*-loop effect on the binding model between bosutinib and SIK2, they could not identify which model was best. Analyzing the various statistics in free energy calculations also enables us to obtain some insights into the behavior of the 10 systems with different initial conformations of the *T*-loop. In this work, the binding free energies of bosutinib to SIK2 were calculated using the MM/GBSA approach. The calculated absolute binding free energies are summarized in Tables 1 and S5-S14 for all ten systems. The binding free energy was between − 12.81 and − 24.72 kcal/mol for the 10 complex systems which means that bosutinib can be bound with different conformations of the *T*-loop for SIK2. For SIK2-III, the calculated binding free energy is approximately − 24.724 kcal/mol, which is the best affinity for bosutinib with SIK2 from our calculations. In contrast, there was weak binding in the bosutinib/SIK2-VI and bosutinib/SIK2-I systems with − 12.82 and − 12.38 kcal/mol, respectively. Meanwhile, the vdW interactions of bosutinib/SIK2-VI and bosutinib/SIK2-I systems were decreased when compared with the SIK2-III system, at 12.33 and 13.06 kcal/mol, respectively. This suggests that the SIK2-I, in which the *T*-loop points towards the ATP-binding site, might be more likely the unfavored model agreement with the RMSD analysis and extracted frames. For SIK2-VI, the protein conformation is induced by the ligand, and the ligand moves to the *T*-loop, which is distant from the hinge loop of SIK2. In contrast, the binding free energy for SIK2-IV (−23.49 kcal/mol) is similar to that of SIK2-III, and the initial conformation of the *T*-loop also had a closed conformation. Bosutinib can bind SIK2 with moderate binding affinity for other systems, excluding SIK2-I, SIK2-III, and SIK2-VI, which binding free energy were between the –23.49 kcal/mol and −13.38 kcal/mol. Bosutinib can bind SIK2 at the intermediate state of the *T*-loop with strong affinity, and the overall closed conformation of the *T*-loop is not a good model. This means that the binding model between inhibitors and kinases needs to be studied for further confirmation of the protein kinases. Nevertheless, we cannot exclude the existence of other models in which the conformation of the kinase can change to fit the inhibitor for binding. In summary, bosutinib could form a strong binding model with SIK2. Only the *T*-loop conformation was considered as the initial state in this work, and the overall conformation of the SIK2 protein was free in the MD simulations. Therefore, the obtained conformation for the bosutinib/SIK2 systems could be used as the target protein conformations for ensemble virtual screening in future studies.

**Table 1:** Binding free energy for bosutinib/SIK2 complexes and decompositions to electrostatic interactions ($E_{ele}$), van der Walls interactions ($E_{vdW}$), solvation free energies ($E_{GB}$), and entropy ($TS_{total}$).

Energies	SIK2-I	SIK2-II	SIK2-III	SIK2-IV	SIK2-V	SIK2-VI	SIK2-VII	SIK2-VIII	SIK2-IX	SIK2-X
$E_{vdW}$	−44.51	−52.07	−57.57	−57.27	−55.60	−45.24	−57.20	−52.41	−55.48	−53.94
$E_{ele}$	–23.84	−12.35	−11.19	−10.60	−9.01	−10.64	−11.02	−9.09	−12.26	−10.11
$E_{GB}$	38.57	27.62	27.51	27.54	27.94	25.37	27.83	25.31	29.96	27.13
$E_{surf}$	−5.45	−6.48	−7.14	−6.79	−6.78	−5.88	−7.02	−6.40	−6.81	−6.86
$G_{gas}$	−68.35	−64.43	−68.76	−67.87	−64.62	−55.88	−68.22	−61.51	−67.76	−64.06
$G_{solv}$	33.12	21.14	20.37	20.74	21.16	19.49	20.81	18.90	23.15	20.27
$E_{gas}+G_{sol}$	−35.23	−43.29	−48.39	−47.13	−43.46	−36.39	−47.41	−42.60	−44.61	−43.78
$TS_{total}$	−21.85	−24.58	–23.67	–23.64	–22.97	–23.59	−25.87	−24.10	−24.54	−24.20
$\Delta G_{bind}^{cal}$	−13.38	−18.70	−24.72	–23.49	−20.49	−12.81	−21.54	−18.50	−20.07	−19.58

In addition, the entropies are all negative (<-21.85 kcal/mol), while the enthalpies are all negative (<-35.23 kcal/mol). This suggests that the formation of binding complexes is an enthalpy-driven process. Usually, there are two major factors that could affect inhibitor binding, that is, polar ($E_{ele}$ + $E_{GB}$) and nonpolar ($E_{vdW}$ + $E_{surf}$). The electrostatic terms were calculated to be approximately − 9.01 to − 23.84 kcal/mol. The electrostatic interactions were canceled by the solvation effects from the polar at approximately 14.73 to 18.93 kcal/mol. Positive values for the polar contributions indicate that polar interactions between the ligand and receptor clearly disfavor this binding. In contrast, van der Waals interactions make dominant and favorable contributions to the binding affinity, as the main contributor of the nonpolar part. The calculated total nonpolar terms are − 49.96 to − 64.71 kcal/mol. Interestingly, much larger nonpolar terms for SIK2-III (−64.71 kcal/mol) than the others can be obtained in our simulations. This is also consistent with our deduction of a stronger binding affinity for SIK2-III than other models. The structural features of aromatics included in the cavity revealed by MD simulations supports the hypothesis that hydrophobic interactions are the main contributors to the binding of the inhibitor with the enzyme. In conclusion, we determined that hydrophobic interactions are the dominant factor in bosutinib binding to SIK2.

## 4 Conclusion

The conformation of the active pocket in the kinase domain is important for the design of novel kinase inhibitors. We characterized the conformational rearrangement of the active pocket of SIK2 when bosutinib was bound using HM and MD simulations. Ten different conformations of the *T*-loop were constructed using the Morph server, ranging from opened to closed. These were seen as the initial conformations of the SIK2 that could be utilized to study the rearrangement of the active pocket when bound to bosutinib. Our simulation showed that bosutinib could bind to SIK2 with up or down conformations of the P-loop, while bosutinib could bind to all the tested conformations of the *T*-loop. However, the *T*-loop conformation was induced by bosutinib to obtain the best binding affinity with the closed conformation of the *T*-loop of SIK2. In addition, the αC-helix conformation was induced by the *T*-loop, and the salt bridge formed only with open conformations. The binding affinity of the different conformations was also calculated using the MM/GBSA method. It was found that bosutinib could form a strong binding model with SIK2 and hydrophobic interactions were dominant. This discovery may help to guide the design of novel SIK2 inhibitors. For example, the obtained conformations of SIK2 from bosutinib/SIK2 systems could be used as the target protein conformations for ensemble docking virtual screening in future studies.

## Declaration of Competing Interest

The authors declare that they have no known competing financial interests or personal relationships that could have appeared to influence the work reported in this paper.
