# Supplementary material for: Molecular dynamics simulations of the conformational plasticity in the active pocket of salt-inducible kinase 2 (SIK2) multi-state binding with bosutinib
Source: Comput Struct Biotechnol J. 2022 May 23;20:2574–86. doi: 10.1016/j.csbj.2022.05.039 (PMC9160496; doi:10.1016/j.csbj.2022.05.039)
Supplement: Supplementary data 1 [file mmc1.pdf]

Supporting Information for

Molecular Dynamics Simulations of the Conformational Plasticity in the Active Pocket of Salt-Inducible Kinase 2 (SIK2) Multi-State Binding with Bosutinib

Mingsong Shi[1], Lun Wang[1], Kongjun Liu[1], Yong Chen[1], Mengshi Hu[1], Linyu Yang[1], Jun He[1], Lijuan Chen[1]* and Dingguo Xu[2,3]*

[1]State Key Laboratory of Biotherapy/Collaborative Innovation Center of Biotherapy and Cancer Center, West China Hospital of Sichuan University, Chengdu, Sichuan 610041, China

[2]College of Chemistry, MOE Key Laboratory of Green Chemistry and Technology, Sichuan University, Chengdu, Sichuan 610064, China

[3]Research Center for Material Genome Engineering, Sichuan University, Chengdu, Sichuan 610065, China

***Corresponding authors:** dgxu@scu.edu.cn (D. X.); chenlijuan125@163.com (L. C.)

Content

Method S7

Molecular Dynamics Simulation S7

Cluster Analysis S8

Binding Free Energy Estimation S8

Cross-correlation Analysis S9

Scheme S1. Methods used in this work. S12

Figure S1. Dasatinib and imatinib bound with ABL1 and ABL2. .... S13

Figure S2. Crystal structures for bosutinib binding with protein kinase in the Protein Database Bank S14

Figure S3. T-loop conformation for those protein kinases bound with bosutinib for closed, open and intermediate conformation. S15

Figure S4. Structure for SIK2 with closed conformation of T-loop. S16

Figure S5. Structure for SIK2 with open conformation of T-loop. S17

Figure S6. ERRAT plot of SIK2 for residue-wise analysis of homology model for SIK2-O and SIK2-C. S18

Figure S7. VERIFY3D analysis results for SIK2-O and SIK2-C models. S19

Figure S8. Ramachandran plot of the models. Validation of the model was done by Ramachandran plot analysis using PROCHECK. S20

Figure S9. Diagram of the SIK2-C and SIK2-O models with overall structures and T-loop highlight S21

Figure S10. Root mean square deviation (RMSD) of bosutinib in the ATP-binding site from crystal complex structures. S22

Figure S11. Initial bosutinib/SIK2 complex structures. S23

Figure S12. Root mean square deviation (RMSD) vs Time plot for the 200 ns MD simulation on SIK2 in complex with its inhibitor bosutinib. S24

Figure S13. Gyration radius (RADGYR) of complex for bosutinib with SIK2. .... S25

Figure S14. Surface area of complex for bosutinib with SIK2 .... S26

Figure S15. RMSF variations for Cα atom of SIK2 for bosutinib/SIK2 complex systems from the 200 ns MD simulation. S27

Figure S16. Snapshots of the bosutinib/SIK2-C along the dynamic simulation time for initial, 50, 100, 150, and 200 ns as well as the superimposition conformations. S28

Figure S17. Snapshots of the bosutinib/SIK2-O along the dynamic simulation time for initial, 50, 100, 150, and 200 ns as well as the superimposition conformations. S29

Figure S18. Cluster analysis results for bosutinib/SIK2-C complex system from the last 100 ns simulation.. S30

Figure S19. Cluster analysis results for SIK2-O complex system from the last 100 ns simulation. S31

Figure S20. Ten structures for bosutinib/SIK2 complex system. S32

Figure S21. Root mean square deviation (RMSD) value of heavy atoms of backbone for protein and heavy atoms of inhibitor along 500 ns MD simulation for bosutinib/SIK2 systems. S33

Figure S22. Snapshots of the bosutinib/SIK2-I along the dynamic simulation time for 100, 200, 300, 400, and 500 ns. S34

Figure S23. Superimposition the snapshots of the bosutinib/SIK2-I along the dynamic simulation time for 100, 200, 300, 400, and 500 ns. S35

Figure S24. Snapshots of the bosutinib/SIK2-III along the dynamic simulation time for 240, 260, 280, 300, 320, 340, 360, 380, 400, 420, 440, and 460 ns. S36

Figure S25. Snapshots of the bosutinib/SIK2-V along the dynamic simulation time for 300, 320, 340, 360, 380, 400, 420, 440, 460, 480, and 500 ns. S37

Figure S26. Initial structure and 500th ns frame aligned for bosutinib/SIK2 complex systems. S38

Figure S27. Root mean square deviation (RMSD) value of heavy atoms of backbone for kinase domain within the 500 ns MD simulation for bosutinib/SIK2 systems referenced to the initial conformation for SIK2-I. S39

Figure S28. RMSF of the Cα atoms of SIK2 from simulation trajectory of bosutinib/SIK2 systems with 500 ns MD. S40

Figure S29. Snapshots of the bosutinib/SIK2-II along the dynamic simulation time for 100, 200, 300, 400, and 500 ns. S41

Figure S30. Snapshots of the bosutinib/SIK2-III along the dynamic simulation time for 100, 200, 300, 400, and 500 ns. S42

Figure S31. Snapshots of the bosutinib/SIK2-IV along the dynamic simulation time for 100, 200, 300, 400, and 500 ns. S43

Figure S32. Snapshots of the bosutinib/SIK2-V along the dynamic simulation time for 100, 200, 300, 400, and 500 ns. S44

Figure S33. Snapshots of the bosutinib/SIK2-VI along the dynamic simulation time for 100, 200, 300, 400, and 500 ns. S45

Figure S34. Snapshots of the bosutinib/SIK2-VII along the dynamic simulation time for 100, 200, 300, 400, and 500 ns. S46

Figure S35. Snapshots of the bosutinib/SIK2-VIII along the dynamic simulation time for 100, 200, 300, 400, and 500 ns. S47

Figure S36. Snapshots of the bosutinib/SIK2-IX along the dynamic simulation time for 100, 200, 300, 400, and 500 ns. S48

Figure S37. Snapshots of the bosutinib/SIK2-X along the dynamic simulation time for 100, 200, 300, 400, and 500 ns. S49

Figure S38. Superimposition structure for dasatinib/SIK2 and bosutinib/SIK2. S50

Figure S39. Root mean square deviation (RMSD) for the five representative frames from the ten bosutinib/SIK2 complex systems. S51

Figure S40. Five representative frames from the cluster analysis for bosutinib/SIK2-I systems. S52

Figure S41. Five representative frames from the cluster analysis for bosutinib/SIK2-II systems. S53

Figure S42. Five representative frames from the cluster analysis for bosutinib/SIK2-III systems. S54

Figure S43. Five representative frames from the cluster analysis for bosutinib/SIK2-IV systems. S55

Figure S44. Five representative frames from the cluster analysis for bosutinib/SIK2-V systems. S56

Figure S45. Five representative frames from the cluster analysis for bosutinib/SIK2-VI systems. S57

Figure S46. Five representative frames from the cluster analysis for bosutinib/SIK2-VII systems. S58

Figure S47. Five representative frames from the cluster analysis for bosutinib/SIK2-VIII systems. S59

Figure S48. Five representative frames from the cluster analysis for bosutinib/SIK2-IX systems. S60

Figure S49. Five representative frames from the cluster analysis for bosutinib/SIK2-X systems. S61

Figure S50. Root mean square deviation (RMSD) value of heavy atoms of backbone of protein and of P-loop along 500 ns MD simulation for bosutinib/SIK2 systems. S62

Figure S51. Root mean square deviation (RMSD) value of heavy atoms of backbone of protein and hinge loop of SIK2 along 500 ns MD simulation for bosutinib/SIK2 systems. S63

Figure S52. Distance between ^{27}GKG^{29} in P-loop with the ^{97}EYA^{99} in hinge loop although the 500 ns MD simulation. S64

Figure S53. Residues and atoms label for N30 and K49. S65

Figure S54. Distance between N30 and K49 for bosutinib/SIK2-I systems. S66

Figure S55. Distance between N30 and K49 for bosutinib/SIK2-II systems. S67

Figure S56. Distance between N30 and K49 for bosutinib/SIK2-III, IV, V, VI, VII, VIII, IX and X systems. S68

Figure S57. P-loop with down conformation when the bosutinib bound with CAMKII (PDB ID: 3SOA) and STK10 (PDB ID: 5AJQ). S69

Figure S58. P-loop with up conformation when the bosutinib bound with Src (PDB ID: 4MXO, 4MXX, 4MXY, 4MXZ), STK24 (PDB ID: 4QMN), EHPA2 (PDB ID: 5I9X), Wee1 (PDB ID: 5VC3) and HER3 (PDB ID: 6OP9). S70

Figure S59. P-loop with up conformation (PDB ID: 5EAK) and down conformation (PDB ID: 5KZ7) for MARK2. .... S71

Figure S60. Root mean square deviation (RMSD) value of heavy atoms of backbone of protein and of T-loop along 500 ns MD simulation for bosutinib/SIK2 systems. S72

Figure S61. Snapshots of the bosutinib/SIK2-I, III, V, VI, and VIII systems for T-loop conformation along the dynamic simulation time for 300, 320, 340, 360, 380, 400, 420, 440, 460, 480, and 500 ns. S73

Figure S62. Snapshots of the bosutinib/SIK2-II, IV and X systems for T-loop conformation along the dynamic simulation time for 300, 320, 340, 360, 380, 400, 420, 440, 460, 480, and 500 ns. S74

Figure S63. Snapshots of the bosutinib/SIK2-VII system for T-loop conformation along the dynamic simulation time for 300, 320, 340, 360, 380, 400, 420, 440, 460, 480, and 500 ns.S75

Figure S64. Snapshots of the bosutinib/SIK2-IX system for T-loop conformation along the dynamic simulation time for 300, 320, 340, 360, 380, 400, 420, 440, 460, 480, and 500 ns.S76

Figure S65. Distance between the T-loop and the hinge loop for bosutinib/SIK2 systems. . S77

Figure S66. T-loop conformation for ABL1, closed conformation for imatinib (PDB ID: 2HYY) and open conformation for axitinib (PDB ID: 4TWP). S78

Figure S67. Label for αC-helix of SIK2 in this work and RMSF of the Cα atoms of αC-Helix of SIK2 from simulation trajectory of bosutinib/SIK2 systems with 500 ns MD. S79

Figure S68. Root mean square deviation (RMSD) value of heavy atoms of backbone of protein and of αC-helix along 500 ns MD simulation for bosutinib/SIK2 systems. S80

Figure S69. Label for salt bridge between K49 and E67 of SIK2 in this work. S81

Figure S70. Distance between K49 and E67 for bosutinib/SIK2 systems. S82

Figure S71. Cross-correlation analysis between T-loop and σC-helix for the bosutinib/SIK2 systems. S83

Figure S72. Cross-correlation maps for SIK2 protein of bosutinib/SIK2 complex systems from the last 200 ns of MD simulation for every system. S84

Figure S73. Cluster analysis results for the SIK2-I (bosutinib/SIK2) system. S84

Table S1. Crystal structures for bosutinib binding with protein kinase in the protein database bank. S86

Table S2. Crystal structures for MARK in the protein database bank. S87

Table S3. Root mean square deviation (RMSD) value of the overall protein and the T-loop of SIK2 for the ten bosutinib/SIK2 complex systems although 500 ns MD simulations. S88

Table S4. Distance value of K49 and E67 of SIK2 for the ten bosutinib/SIK2 complex systems although 500 ns MD simulations. S89

Table S5. Binding free energies ($\Delta \mathbf{Gbindcal}$) for bosutinib/SIK2-I complexes. S90

Table S6. Binding free energies ($\Delta \mathbf{Gbindcal}$) for bosutinib/SIK2-II complexes................ S91

Table S7. Binding free energies ($\Delta \mathbf{Gbindcal}$) for bosutinib/SIK2-III complexes. S92

Table S8. Binding free energies ($\Delta \mathbf{Gbindcal}$) for bosutinib/SIK2-IV complexes. S93

Table S9. Binding free energies ($\Delta \mathbf{Gbindcal}$) for bosutinib/SIK2-V complexes. S94

Table S10. Binding free energies ($\Delta \mathbf{Gbindcal}$) for bosutinib/SIK2-VI complexes. S95

Table S11. Binding free energies ($\Delta \mathbf{Gbindcal}$) for bosutinib/SIK2-VII complexes. S96

Table S12. Binding free energies ($\Delta \mathbf{Gbindcal}$) for bosutinib/SIK2-VIII complexes. S97

Table S13. Binding free energies ($\Delta \mathbf{Gbindcal}$) for bosutinib/SIK2-IX complexes. S98

Table S14. Binding free energies ($\Delta \mathbf{Gbindcal}$) for bosutinib/SIK2-X complexes. S99

Method

Molecular Dynamics Simulation

To correctly describe the bosutinib force field, the general Amber force field (GAFF2)[1] generation procedure was used. The geometry structure was optimized at B3LYP/6-31G level of theory using Gaussian 09[2]. To obtain the partial atomic charges, the restrained electrostatic potential (RESP) protocol[3] was employed at the HF/6-31G* level of theory. The force field parameters were generated using the Antechamber module. Meanwhile, the AMBER ff19SB force field[4] was used to create topology parameters of protein SIK2. Firstly, the complex systems were dissolved in a TIP3P water[5] with cuboid box, and the total box size was about 87 Å × 88 Å × 105 Å. The systems were neutralized using the Na^+ ions and the final system included 5277 solute atoms and about 20000 solvent water molecules. The periodic boundary conditions were applied to avoid edge effects and a cut-off radius of 12 Å was employed for van der Waals interactions. The particle mesh Ewald (PME) algorithm[6] also was used in calculating the long-range electrostatic interactions. The SHAKE algorithm[7] was used to constrain the covalent bond involved with hydrogen atoms. To reduce the effect of unfavorable interactions produced by solvents and ion, the system was subjected to 9000 steps of steepest descent method and then 1000-step conjugate gradient, while fixing all the solute molecules at the initial position. Then the 10000-step conjugate gradient method was used to optimize the whole system including solute molecule and solvent molecule. After the first two-step minimization of the system, the overall system temperature was heated from 0 K to 300 K in 200 ps with Langevin dynamics [8, 9]with the collision frequency g = 2.0 ps^{-1}. The pressure was then kept at 1 bar in 200 ps with isotropic position scaling[10] and temperature at 300 K. After that, the system was equilibrated at 300 K and 1 bar within the NPT ensemble with 200 ps. Subsequently, the entire system underwent the 500 ns molecular dynamics simulation for final data collection and analyses. The integration step size was set to 2 fs throughout the molecular dynamics. All dynamics were performed using the CUDA version of the PMEMD in AMBER 20[11]. The *CPPTRAJ* module[12, 13] was used to analysis the data from the MD trajectories.

Cluster Analysis

Cluster analysis is a general unsupervised technique for finding patterns within data. In this work, the root mean-square deviation (RMSD)-based clustering was performed with *ptraj[12, 13]*, a simulation analysis tools implemented in AMBERTools20[11]. As one of the most popular clustering algorithms, the average linkage cluster algorithm was used herein. Each cluster contained a representative structure whose RMSD was equidistant to all other cluster members. Structures were collected by sampling at 100 ps intervals, and cluster analysis was based on the heavy atoms of SIK2 to generate a sum of five clusters (only one clusters were kept in the following studies). Meanwhile, the cluster numbers five, ten, and fifteen for the SIK2-I system were selected to test the power of cluster analysis in this work, which shown in Figure S73.

Binding Free Energy Estimation

In this work, the molecular mechanics generalized Born surface area (MM/GBSA) approach[14, 15] were employed to calculate the binding free energies of those four inhibitors to SIK2 protein kinase. It is an efficient method to improve the ability evaluation of ligand and enzyme systems[16-18]. The MM/GBSA framework has been discussed extensively[19-21]. Only a short description is summarized here. The total binding free energy for the binding of inhibitor of SIK2, namely $\Delta G_{binding}$ was provide in the following:

$$\Delta G_{binding} = \Delta G_{complex} - \Delta G_{protein} - \Delta G_{ligand} \quad (1)$$

$$G = E_{gas} + G_{sol} - TS \quad (2)$$

$$E_{gas} = E_{int} + E_{vdW} + E_{ele} \quad (3)$$

$$G_{sol} = G_{el} + G_{nonel} \quad (4)$$

$\Delta G_{complex}$, $\Delta G_{protein}$ and ΔG_{ligand} are denoted as free energies of the inhibitor/SIK2, SIK2 and inhibitor, respectively. The $\Delta G_{binding}$ can be decomposed into the enthalpy part ($\Delta H = E_{gas} + G_{sol}$) and the entropy part ($T\Delta S$). The molecular mechanical energies (E_{gas}) consist of the intramolecular energy (E_{int}), van der Waals forces (E_{vdW}) and electrostatic forces (E_{ele}) which get these values via a statistical average way based on the AMBER force field. The solvation free energy (G_{sol}) can be principally divided into both electrostatic (G_{el}) and non-electrostatic (G_{nonel}) terms.

The G_{nonel} comes from the combined effect of unfavorable cost of surface formation and the favorable van der Waals interactions between the solute and solvent, which can be evaluated by the equation of $\gamma \cdot SA + b$, where γ =0.0072 kcal/Å^2 and b=0.0 kcal/mol. The solvent accessible surface area (SA) which was estimated using the LCPO method[22]. The G_{el} is calculated by the Generalized Born (GB) equation[23, 24]. The solute dielectric constant was set to 1, and the exterior dielectric constant was set to 80. We used MM/GBSA method to get these former terms via a statistical average way from the last 200 ns MD trajectory. Meanwhile, entropy contributions to the binding free energy may be added to improve the accuracy. The entropy can be estimated using the normal model analysis with quasi harmonic model based on the conformational snapshots from the same MD trajectory[25]. For each complex system, binding energies were averaged over 1000 frames and the $-TS$ was averaged with interval 2 ns. Those energy was calculated with the MMPBA.py program[26].

Cross-correlation Analysis

The cross-correlations between the residue fluctuations in the T-loop (Residues: 160-180) and the residues in the σC-helix (Residues: 58-73) were calculated along with the simulation time. In this work, every 1 ns was defined as a window. The next window included 500 ps from the last 500 ps in the current window. For example, the fist window was for the 1–1000 ps; the second for 501–1500 ps; the third for 1000–2000 ps; and so on. In total there are 998 windows for every bosutinib/SIK2 system. In the present study, the dynamic cross correlation value ranged from −1 to 1. The positive values represent pairs of residues moving in the same direction. Meanwhile, the negative values represent pairs of residues moving in the opposite direction. The higher the absolute cross-correlation value is, the more the two residues are correlated (or anticorrelated). While the cross-correlation value with 0 means that the motions of the residues are completely uncorrelated.

Reference

1. Wang JM, Wolf RM, Caldwell JW, Kollman PA, Case DA (2004) Development and testing of a general amber force field. J Comput Chem 25(9):1157-1174.

2. Frisch MJ, Trucks GW, Schlegel HB, Scuseria GE, Robb MA, et al. Gaussian 09. Wallingford, CT, USA: Gaussian, Inc.; 2009.

3. Bayly CI, Cieplak P, Cornell WD, Kollman PA (1993) A well-behaved electrostatic potential based method using charge restraints for deriving atomic charges: the RESP model. J Phys Chem 97(40):10269-10280.

4. Tian C, Kasavajhala K, Belfon KAA, Raguette L, Huang H, et al. (2020) ff19SB: Amino-Acid-Specific Protein Backbone Parameters Trained against Quantum Mechanics Energy Surfaces in Solution. Journal of Chemical Theory and Computation 16(1):528-552.

5. Jorgensen WL, Chandrasekhar J, Madura JD, Impey RW, Klein ML (1983) Comparison of simple potential functions for simulating liquid water. The Journal of Chemical Physics 79(2):926-935.

6. Darden T, York D, Pedersen L (1993) Particle mesh Ewald: An N · log(N) method for Ewald sums in large systems. The Journal of Chemical Physics 98(12):10089-10092.

7. Ryckaert J-P, Ciccotti G, Berendsen HJC (1977) Numerical integration of the cartesian equations of motion of a system with constraints: molecular dynamics of n-alkanes. Journal of Computational Physics 23(3):327-341.

8. Feller SE, Zhang YH, Pastor RW, Brooks BR (1995) Constant pressure molecular dynamics simulation: The Langevin piston method. J Chem Phys 103(11):4613-4621.

9. Martyna GJ, Tobias DJ, Klein ML (1994) Constant pressure molecular dynamics algorithms. J Chem Phys 101(5):4177-4189.

10. Berendsen HJC, Postma JPM, Vangunsteren WF, Dinola A, Haak JR (1984) Molecular dynamics with coupling to an external bath. J Chem Phys 81(8):3684-3690.

11. D.A. Case KB, I.Y. Ben-Shalom, S.R. Brozell, D.S. Cerutti, T.E. Cheatham, III, V.W.D. Cruzeiro, T.A. Darden, R.E. Duke, G. Giambasu, M.K. Gilson, H. Gohlke, A.W. Goetz, R. Harris, S. Izadi, S.A. Izmailov, K. Kasavajhala, A. Kovalenko, R. Krasny, T. Kurtzman, T.S. Lee, S. LeGrand, P. Li, C. Lin, J. Liu, T. Luchko, R. Luo, V. Man, K.M. Merz, Y. Miao, O. Mikhailovskii, G. Monard, H. Nguyen, A. Onufriev, F.Pan, S. Pantano, R. Qi, D.R. Roe, A. Roitberg, C. Sagui, S. Schott-Verdugo, J. Shen, C. Simmerling, N.R.Skrynnikov, J. Smith, J. Swails, R.C. Walker, J. Wang, L. Wilson, R.M. Wolf, X. Wu, Y. Xiong, Y. Xue, D.M. York and P.A. Kollman (2020) AMBER 2020, University of California, San Francisco.

12. Roe DR, Cheatham TE (2018) Parallelization of CPPTRAJ Enables Large Scale Analysis of Molecular Dynamics Trajectory Data. J Comput Chem 39(25):2110-2117.

13. Roe DR, Cheatham TE (2013) PTRAJ and CPPTRAJ: Software for Processing and Analysis of Molecular Dynamics Trajectory Data. Journal of Chemical Theory and Computation 9(7):3084-3095.

14. Srinivasan J, Cheatham TE, Cieplak P, Kollman PA, Case DA (1998) Continuum solvent studies of the stability of DNA, RNA, and phosphoramidate - DNA helices. J Am Chem Soc 120(37):9401-9409.

15. Lee MS, Salsbury FR, Olson MA (2004) An efficient hybrid explicit/implicit solvent method for biomolecular simulations. J Comput Chem 25(16):1967-1978.

16. Shi M, Xu D (2019) Molecular dynamics investigations suggest a non-specific recognition strategy of 14-3-3σ protein by tweezer: Implication for the inhibition mechanism. Front Chem 7(237).

17. Wang JY, Chen Q, Wang M, Zhong C (2017) The opening/closure of the P-loop and hinge of BCR-ABL1 decodes the low/high bioactivities of dasatinib and axitinib. Phys Chem Chem Phys 19(33):22444-22453.

18. Tse A, Verkhivker GM (2015) Molecular Dynamics Simulations and Structural Network Analysis of c-Abl and c-Src Kinase Core Proteins: Capturing Allosteric Mechanisms and Communication Pathways from Residue Centrality. J Chem Inf Model 55(8):1645-1662.

19. Honig B, Nicholls A (1995) Classical electrostatics in biology and chemistry. Science 268(5214):1144-1149.

20. Genheden S, Ryde U (2015) The MM/PBSA and MM/GBSA methods to estimate ligand-binding affinities. Expert Opin Drug Discov 10(5):449-461.

21. Onufriev AV, Case DA. Generalized Born Implicit Solvent Models for Biomolecules. In: Dill KA, editor. Annual Review of Biophysics, Vol 48. Annual Review of Biophysics. 48. Palo Alto: Annual Reviews; 2019. p. 275-296.

22. Weiser J, Shenkin PS, Still WC (1999) Approximate atomic surfaces from linear combinations of pairwise overlaps (LCPO). J Comput Chem 20(2):217-230.

23. Still WC, Tempczyk A, Hawley RC, Hendrickson T (1990) Semianalytical treatment of solvation for molecular mechanics and dynamics. J Am Chem Soc 112(16):6127-6129.

24. Srinivasan J, Trevathan MW, Beroza P, Case DA (1999) Application of a pairwise generalized Born model to proteins and nucleic acids: inclusion of salt effects. Theor Chem Acc 101(6):426-434.

25. Gao PC, Li ZL (2019) Computation of the Boltzmann entropy of a landscape: a review and a generalization. Landsc Ecol 34(9):2183-2196.

26. Miller BR, McGee TD, Swails JM, Homeyer N, Gohlke H, et al. (2012) MMPBSA.py: An Efficient Program for End-State Free Energy Calculations. Journal of Chemical Theory and Computation 8(9):3314-3321.

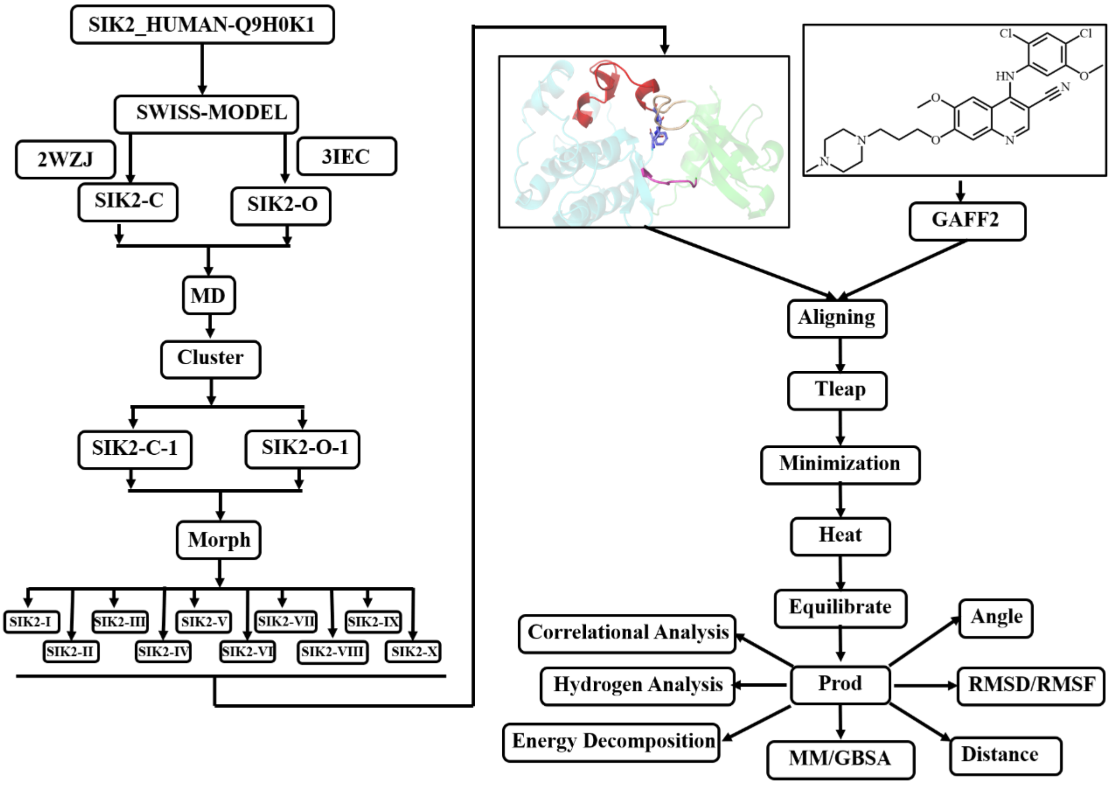

Scheme S1. Methods used in this work.

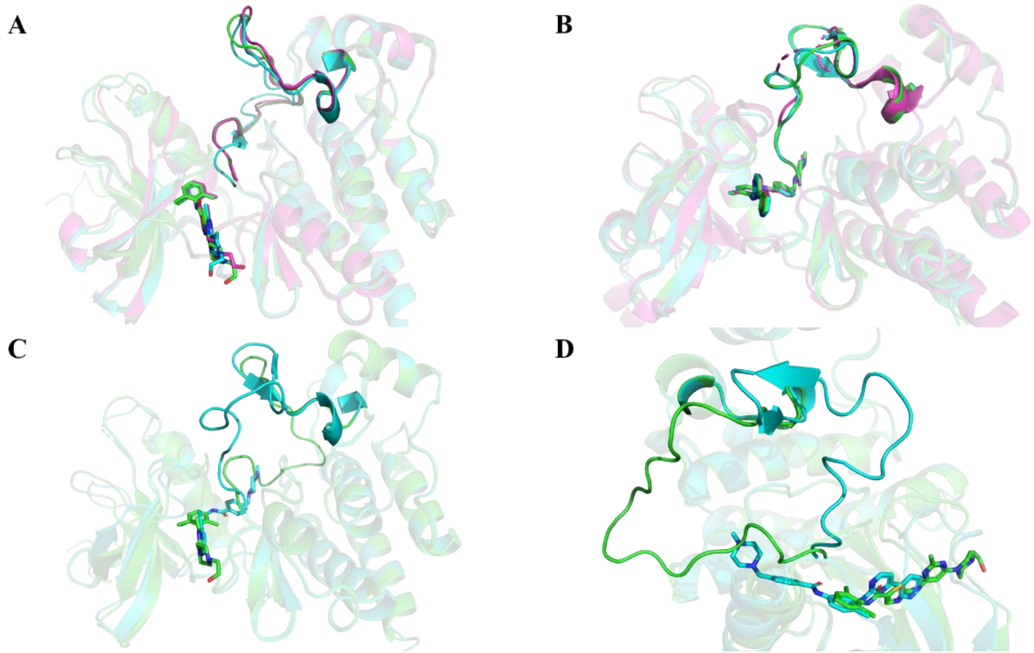

Figure S1. Dasatinib and imatinib bound with ABL1 and ABL2.

(A): dasatinib bound with ABL1 (PDB ID: 2GQG with green and 4XEY with cyan) and ABL2 (PDB ID: 4XLI with magenta); (B): imatinib bound with ABL1 (PDB ID: 2HYY with green and 1IEP with cyan) and ABL2 (PDB ID: 3GVU with magenta); (C): dasatinib (PDB ID: 2GQG with green) and imatinib (PDB ID: 2HYY with cyan). (D): zooming the T-loop region with C. Cartoon shown with protein and stick with dasatinib and imatinib.

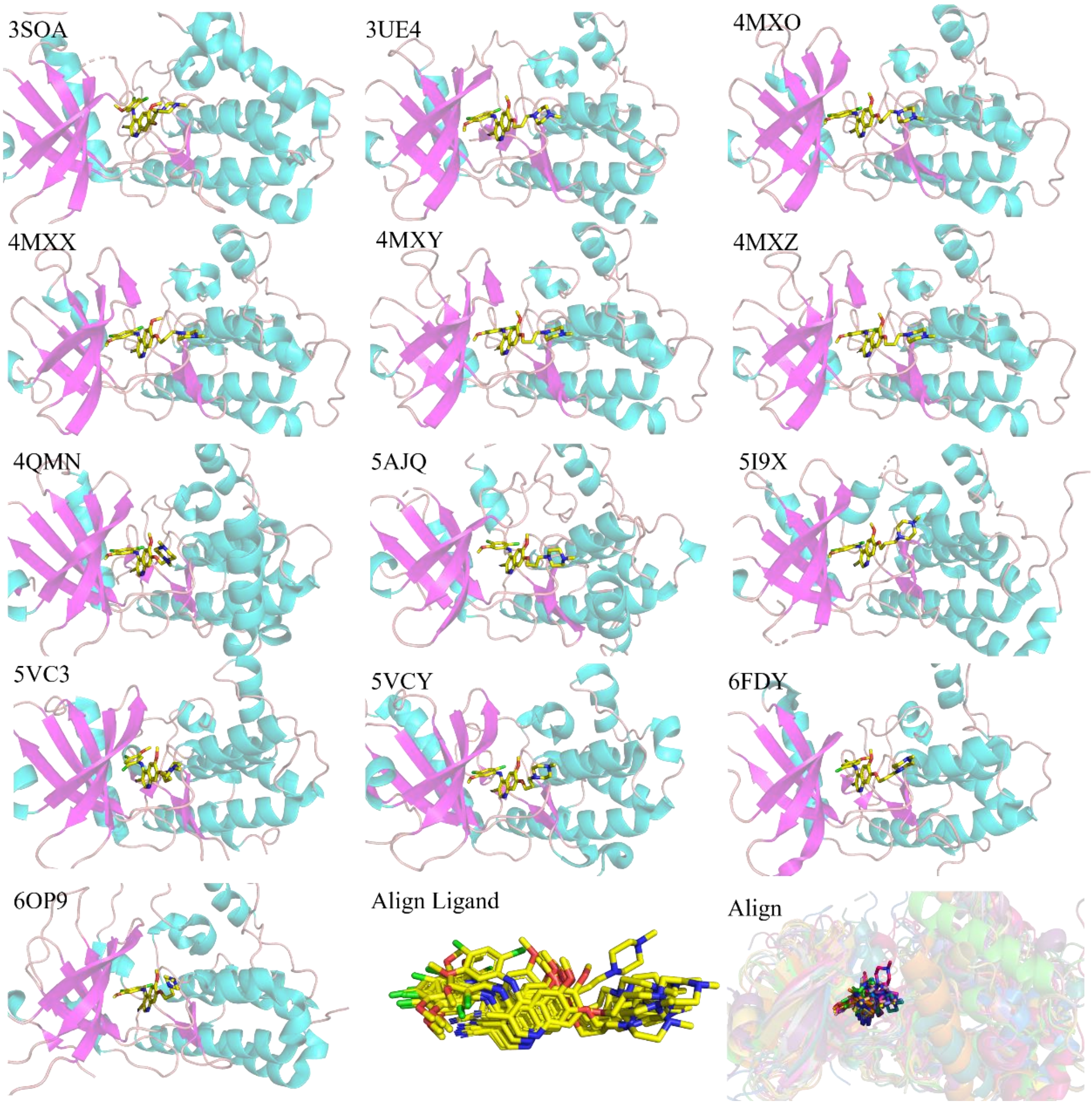

Figure S2. Crystal structures for bosutinib binding with protein kinase in the Protein Database Bank.

The date for searching is 2021-12-08. The ligand ID for bosutinib is DB8.

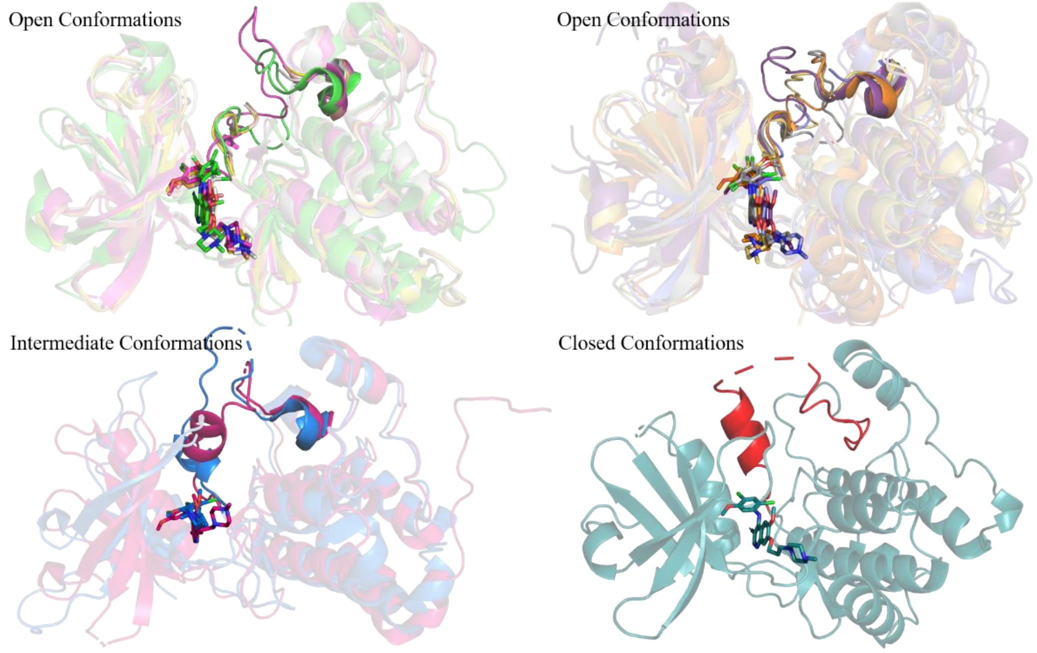

Figure S3. T-loop conformation for those protein kinases bound with bosutinib for closed, open and intermediate conformation.

The conformations of the T-loop were diverse, such as 3SOA, 3UE4, 4MXO, 4MXX, 4MXY, 4MXZ, 4QMN, 5VC3, 5VCY, and 6FDY which were open conformations, 5AJQ a closed conformation, and 5I9X and 6OP9 which were intermediate states.

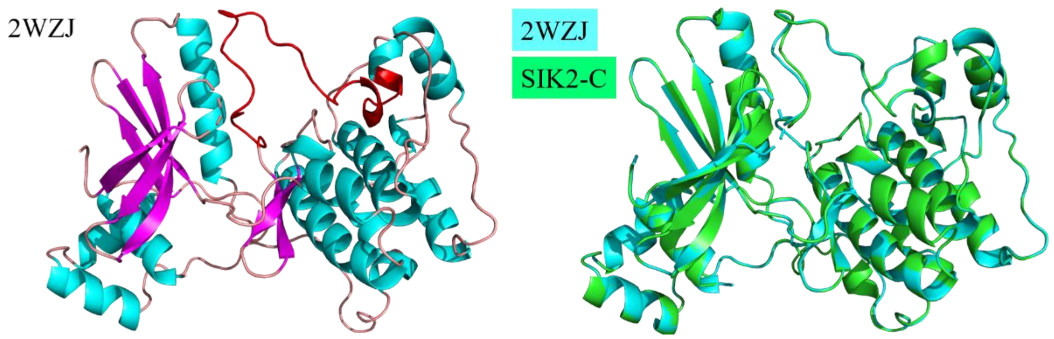

Figure S4. Structure for SIK2 with closed conformation of T-loop.

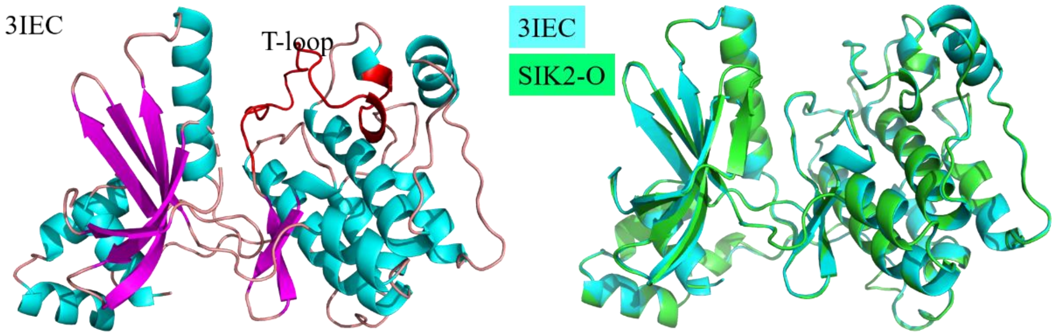

Figure S5. Structure for SIK2 with open conformation of T-loop.

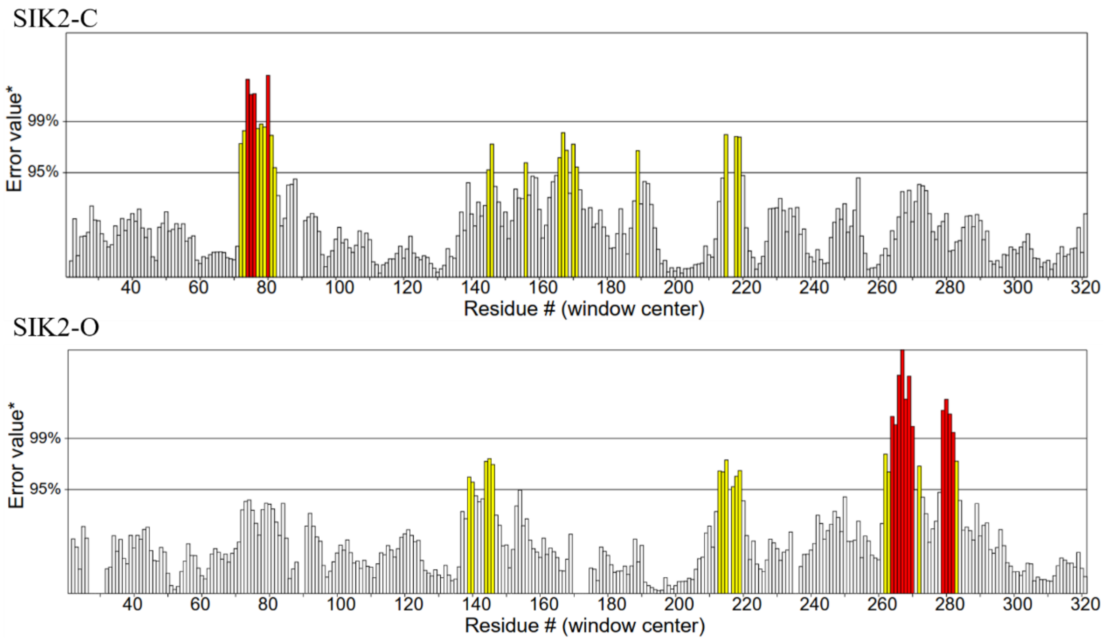

Figure S6. ERRAT plot of SIK2 for residue-wise analysis of homology model for SIK2-O and SIK2-C.

SIK2-C

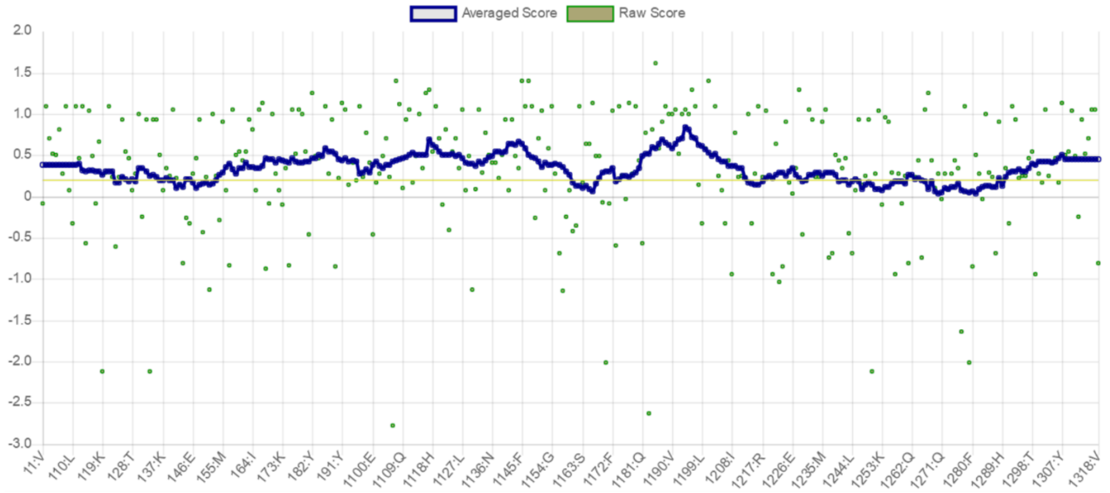

SIK2-O

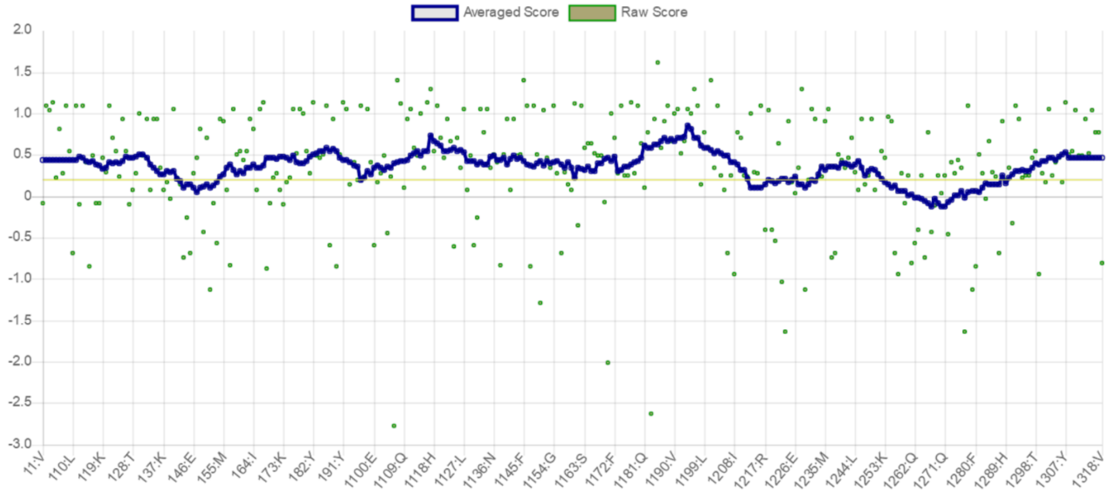

Figure S7. VERIFY3D analysis results for SIK2-O and SIK2-C models.

For SIK2-C, fewer than 80% of the amino acids have scored >= 0.2 in the 3D/1D profile. For SIK2-O, more than 80% of the amino acids have scored >= 0.2 in the 3D/1D profile.

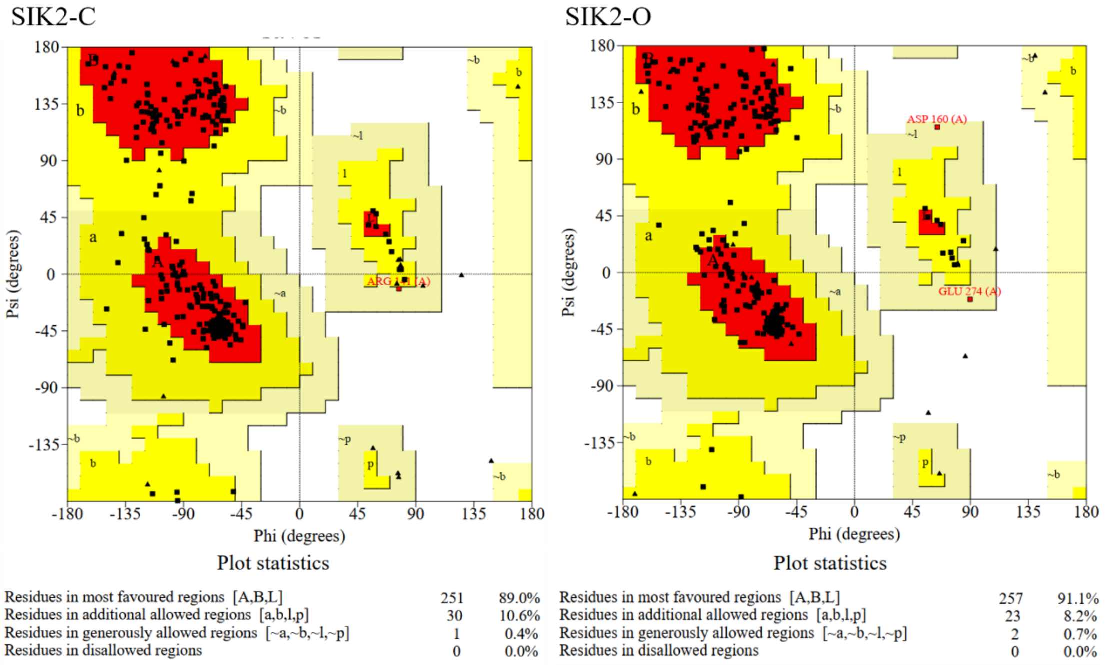

Figure S8. Ramachandran plot of the models. Validation of the model was done by Ramachandran plot analysis using PROCHECK.

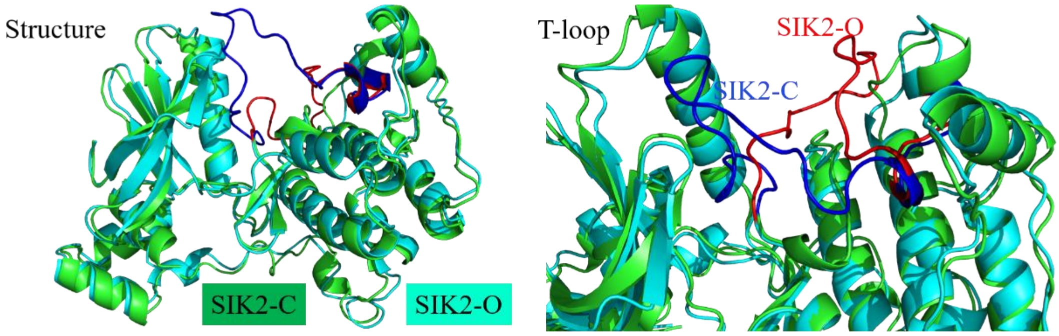

Figure S9. Diagram of the SIK2-C and SIK2-O models with overall structures and T-loop highlight.

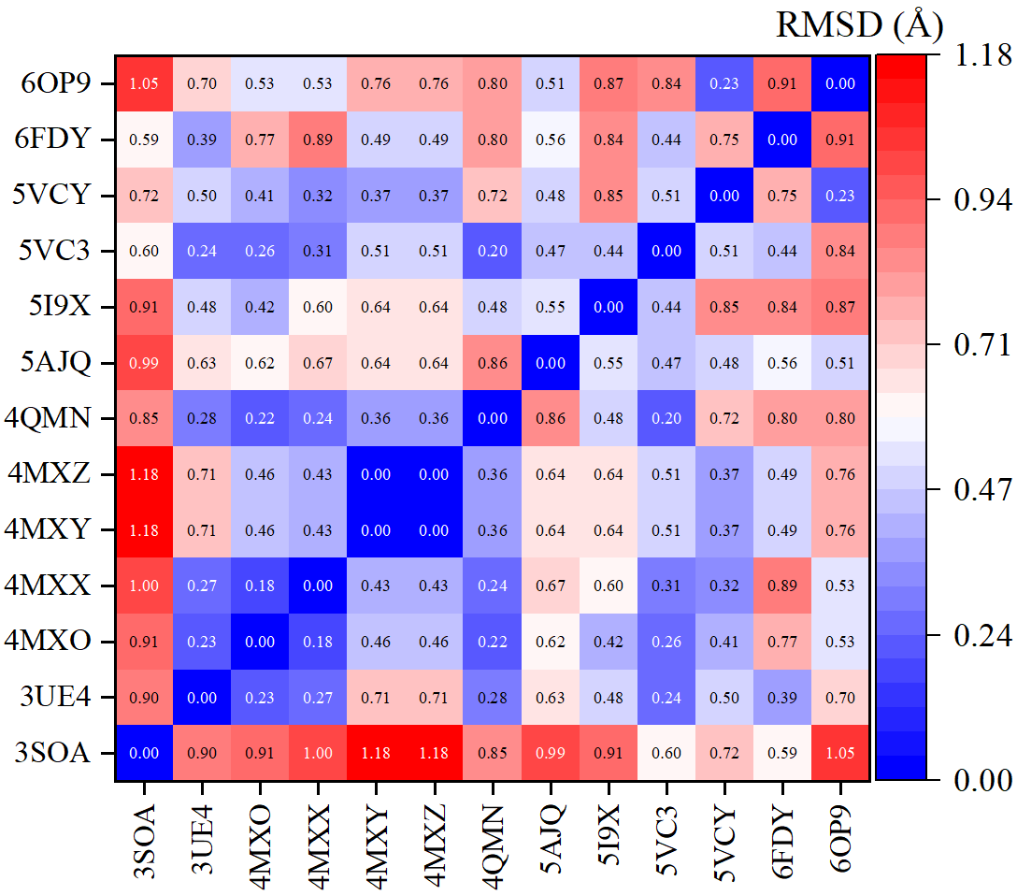

Figure S10. Root mean square deviation (RMSD) of bosutinib in the ATP-binding site from crystal complex structures.

The RMSD of bosutinib has been calculated after the superposition of the crystal complex structures. The structures of bosutinib were labeled with the PDB ID with the complex structure.

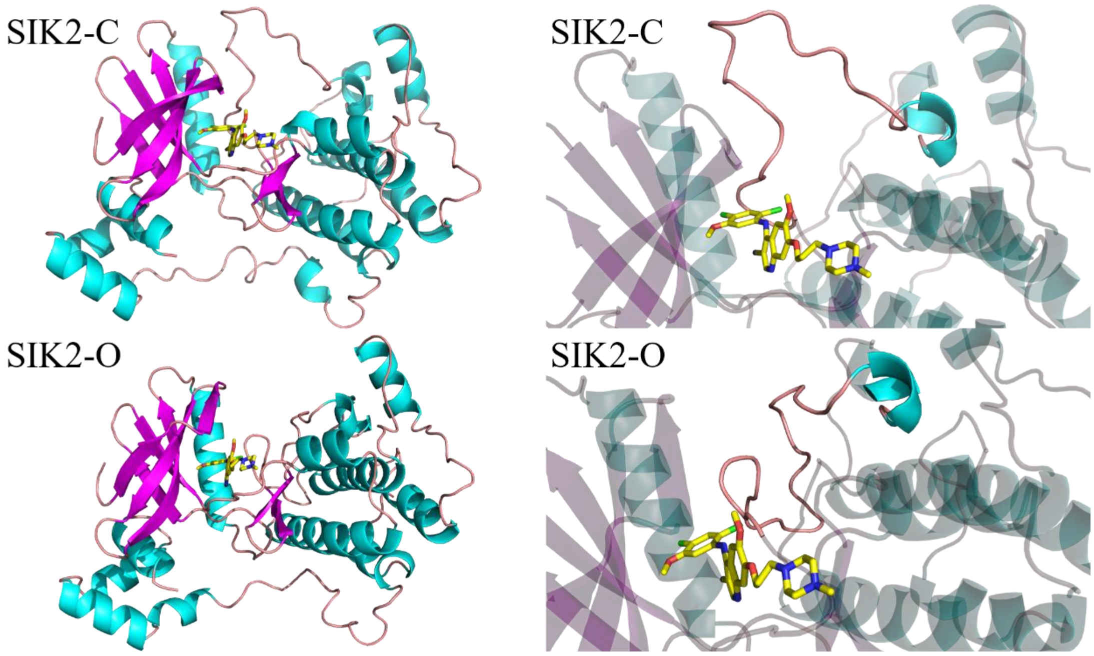

Figure S11. Initial bosutinib/SIK2 complex structures.

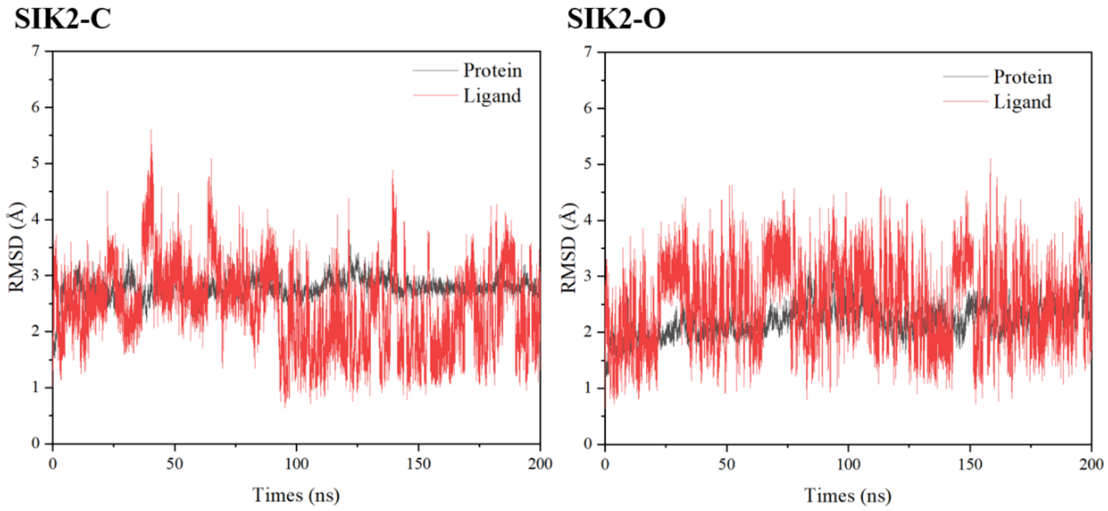

Figure S12. Root mean square deviation (RMSD) vs Time plot for the 200 ns MD simulation on SIK2 in complex with its inhibitor bosutinib.

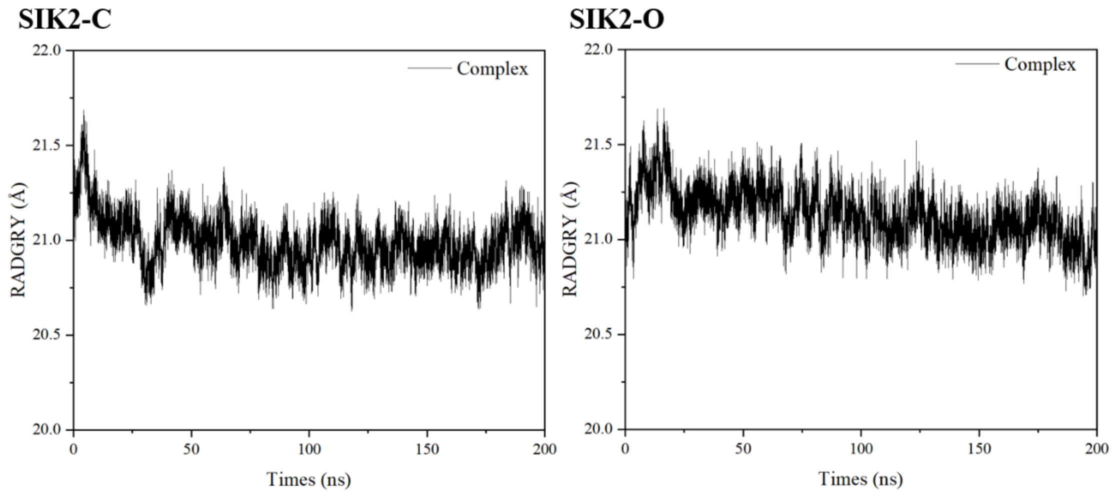

Figure S13. Gyration radius (RADGYR) of complex for bosutinib with SIK2.

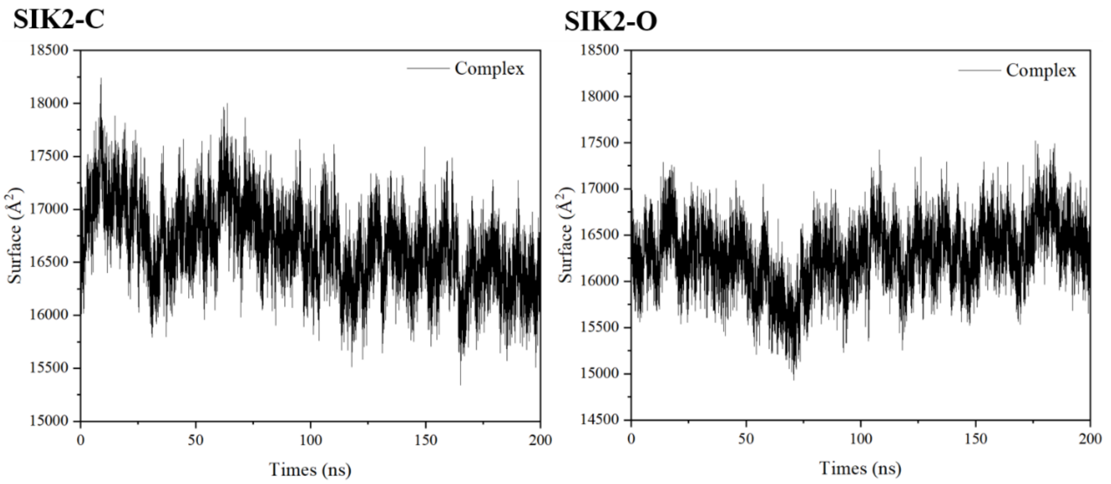

Figure S14. Surface area of complex for bosutinib with SIK2.

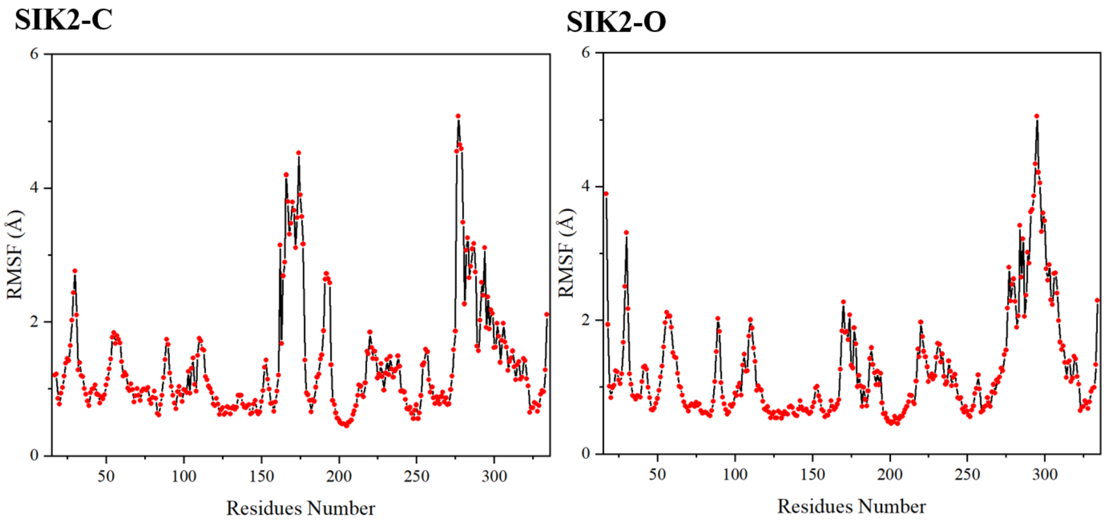

Figure S15. RMSF variations for Cα atom of SIK2 for bosutinib/SIK2 complex systems from the 200 ns MD simulation.

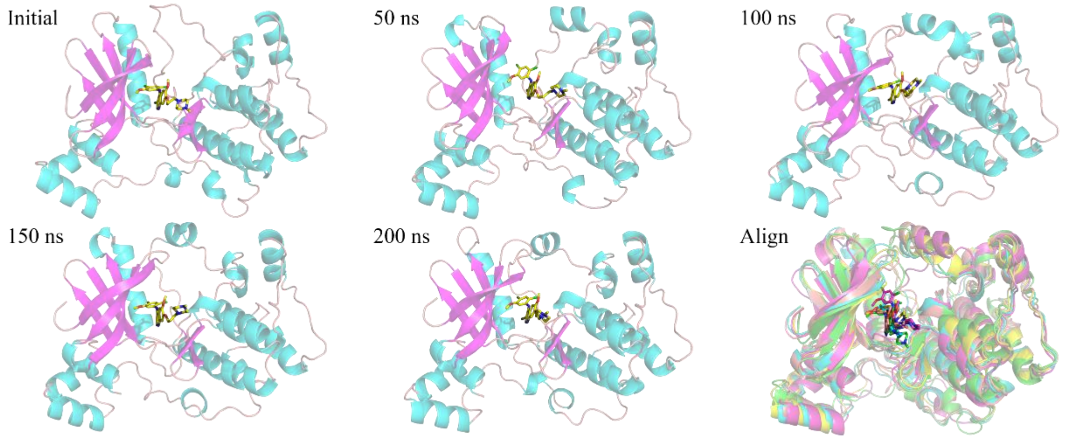

Figure S16. Snapshots of the bosutinib/SIK2-C along the dynamic simulation time for initial, 50, 100, 150, and 200 ns as well as the superimposition conformations.

For clarity, the water molecules have been removed. The bosutinib is plotted using stick style, while cartoon style for SIK2.

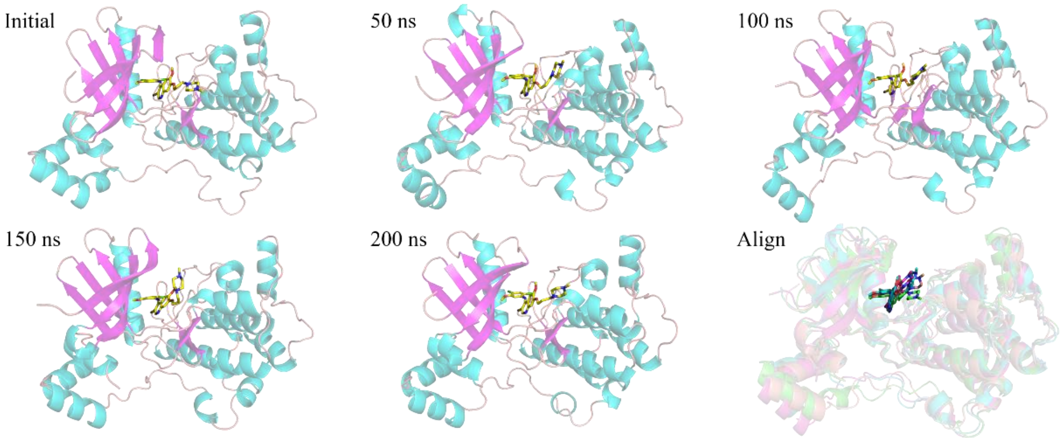

Figure S17. Snapshots of the bosutinib/SIK2-O along the dynamic simulation time for initial, 50, 100, 150, and 200 ns as well as the superimposition conformations.

For clarity, the water molecules have been removed. The bosutinib is plotted using stick style, while cartoon style for SIK2.

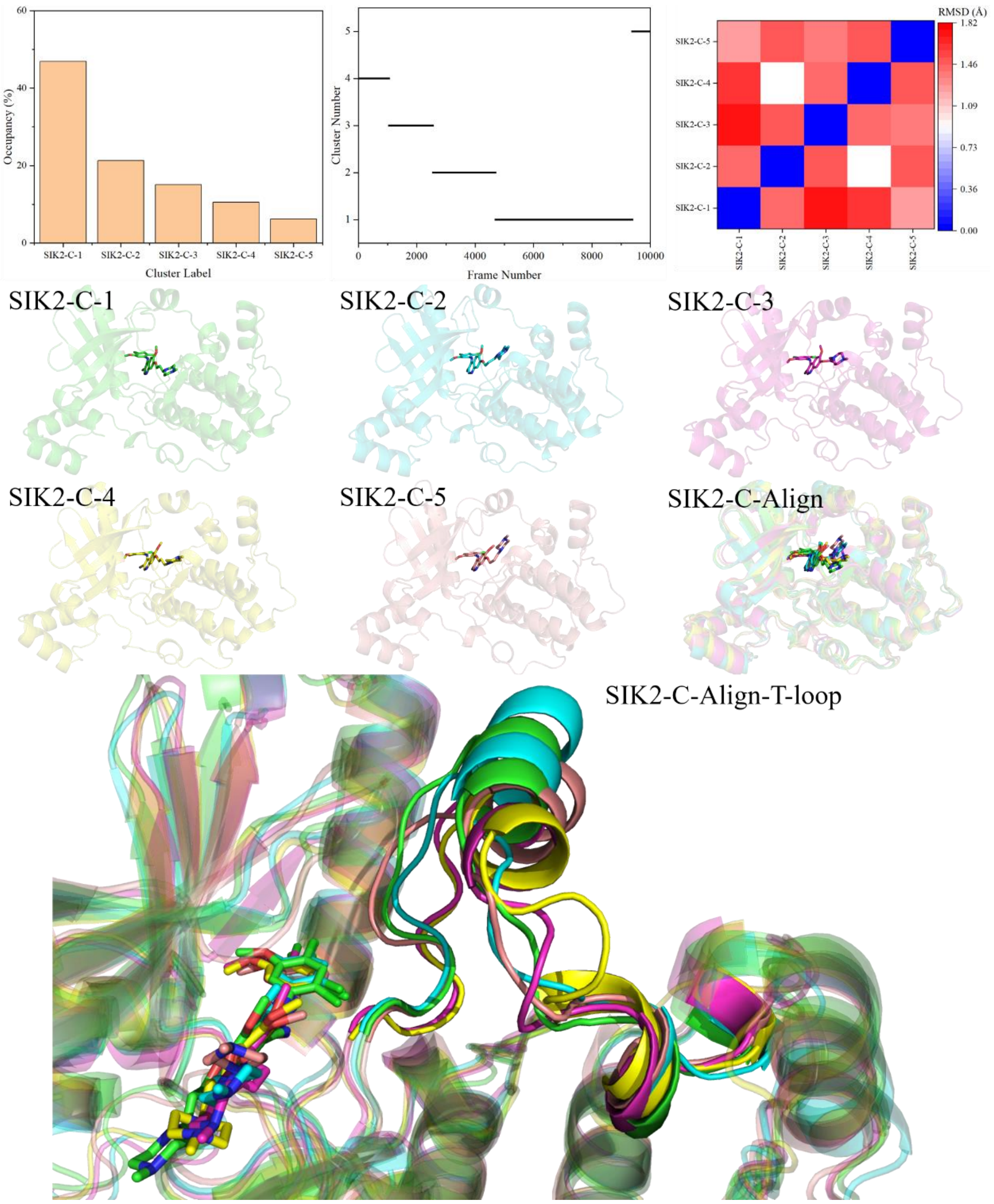

Figure S18. Cluster analysis results for bosutinib/SIK2-C complex system from the last 100 ns simulation.

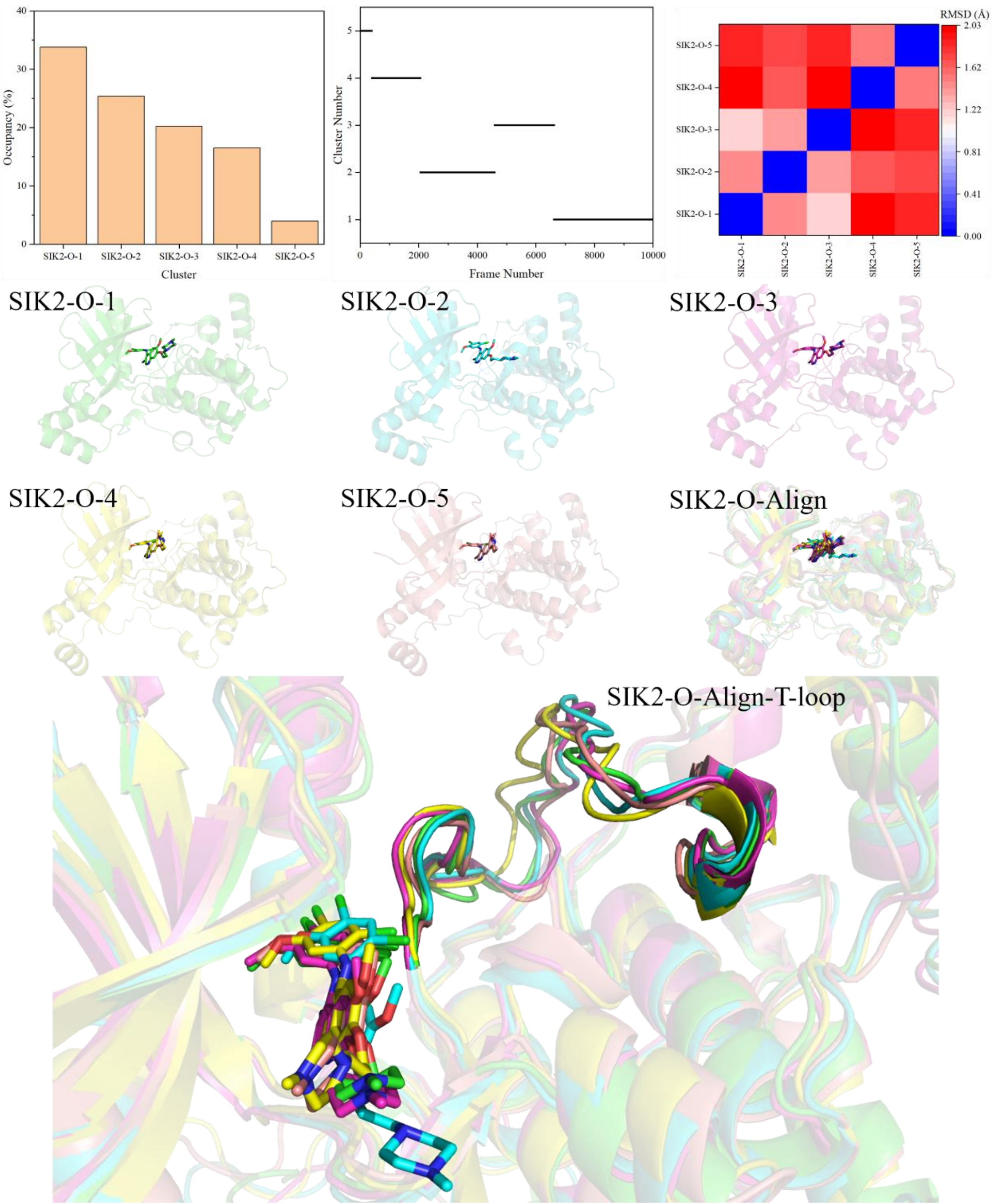

Figure S19. Cluster analysis results for SIK2-O complex system from the last 100 ns simulation.

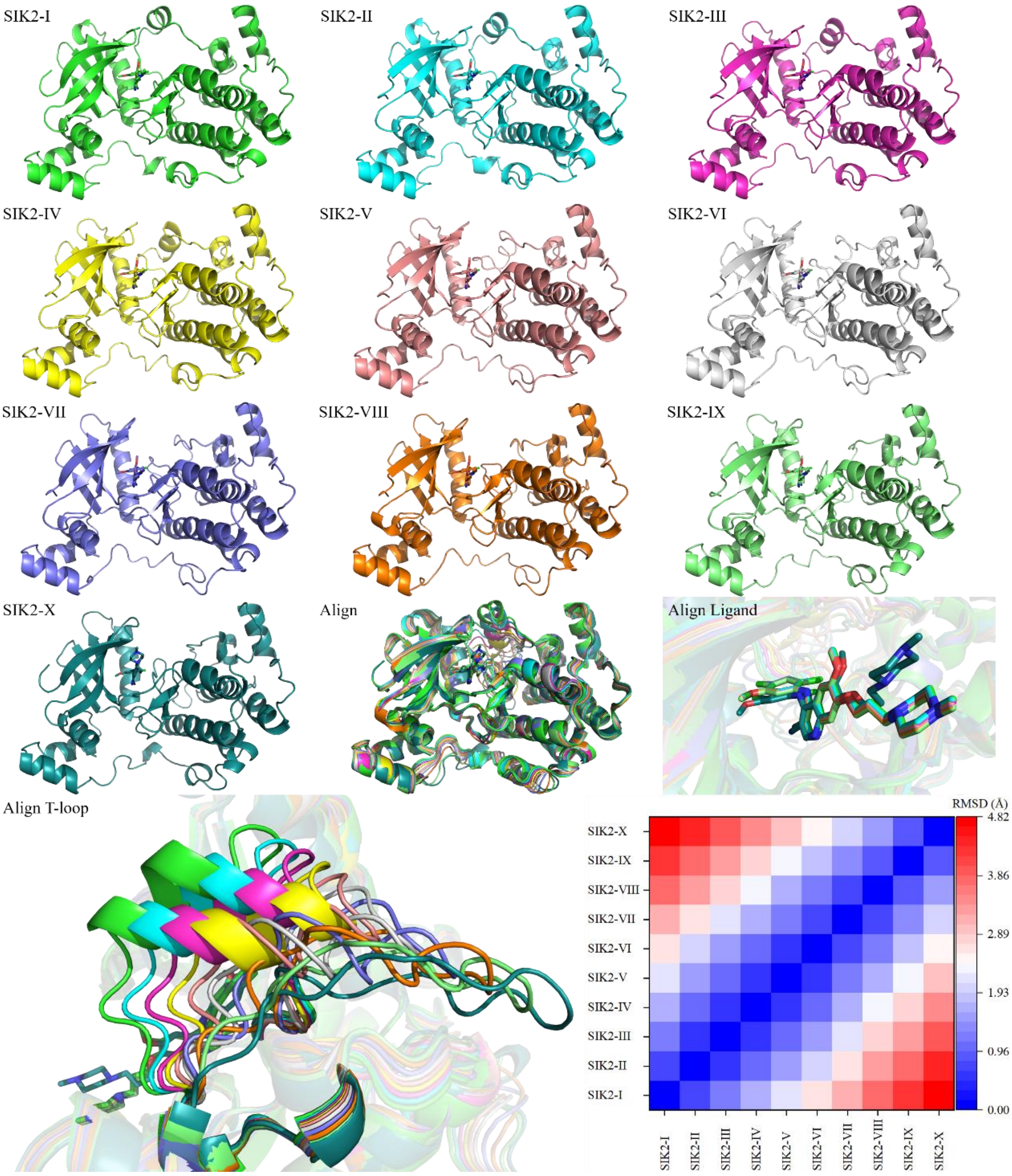

Figure S20. Ten structures for bosutinib/SIK2 complex system.

The eight intermediate conformation between closed and open systems from the Yale Morph Server.

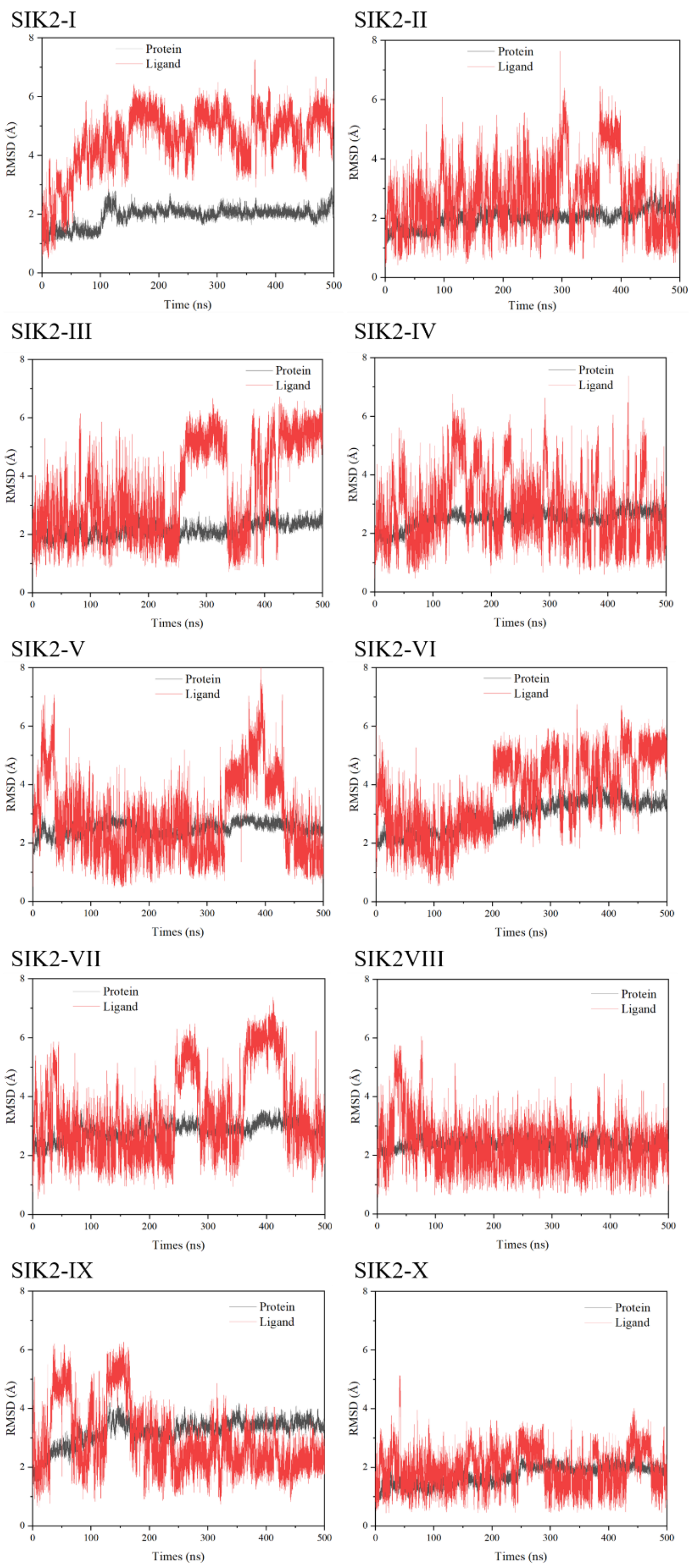

Figure S21. Root mean square deviation (RMSD) value of heavy atoms of backbone for protein and heavy atoms of inhibitor along 500 ns MD simulation for bosutinib/SIK2 systems.

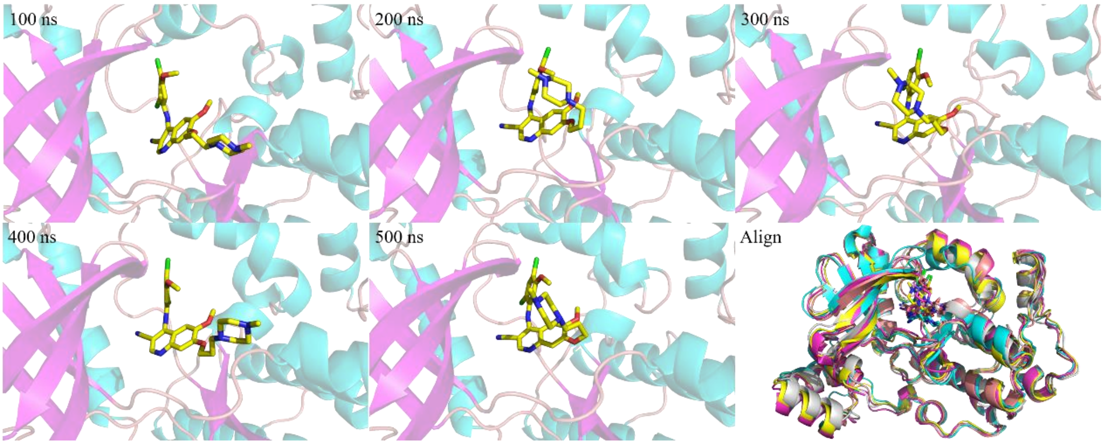

Figure S22. Snapshots of the bosutinib/SIK2-I along the dynamic simulation time for 100, 200, 300, 400, and 500 ns.

For clarity, the water molecules have been removed. The inhibitor is plotted using stick style, while cartoon style for SIK2.

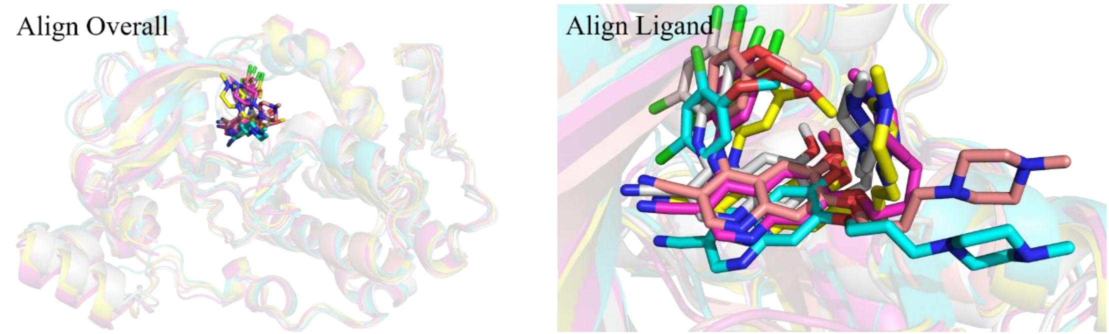

Figure S23. Superimposition the snapshots of the bosutinib/SIK2-I along the dynamic simulation time for 100, 200, 300, 400, and 500 ns.

For clarity, the water molecules have been removed. The inhibitor is plotted using stick style, while cartoon style for SIK2.

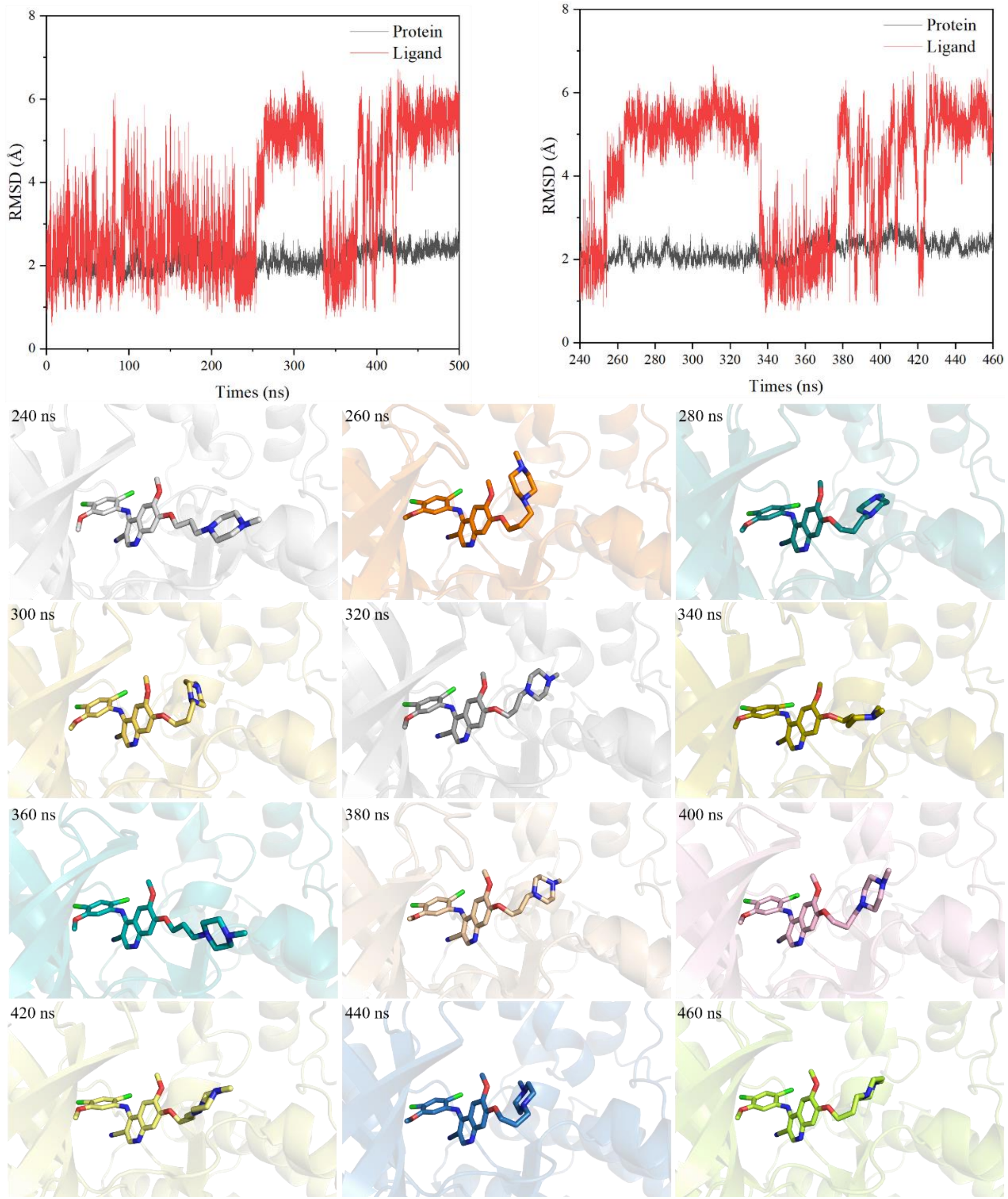

Figure S24. Snapshots of the bosutinib/SIK2-III along the dynamic simulation time for 240, 260, 280, 300, 320, 340, 360, 380, 400, 420, 440, and 460 ns.

For clarity, the water molecules have been removed. The inhibitor is plotted using stick style, while cartoon style for SIK2.

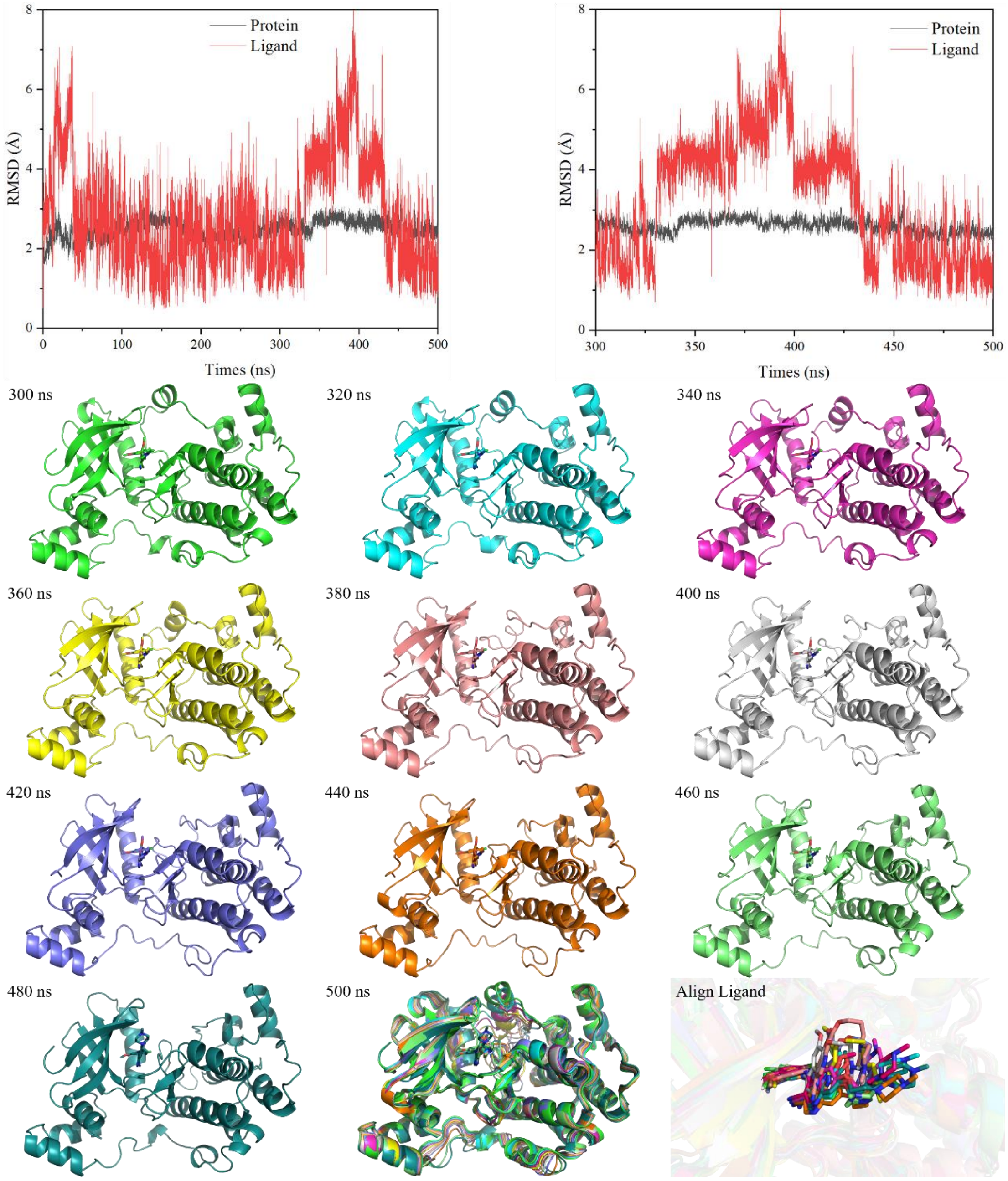

Figure S25. Snapshots of the bosutinib/SIK2-V along the dynamic simulation time for 300, 320, 340, 360, 380, 400, 420, 440, 460, 480, and 500 ns.

For clarity, the water molecules have been removed. The inhibitor is plotted using stick style, while cartoon style for SIK2.

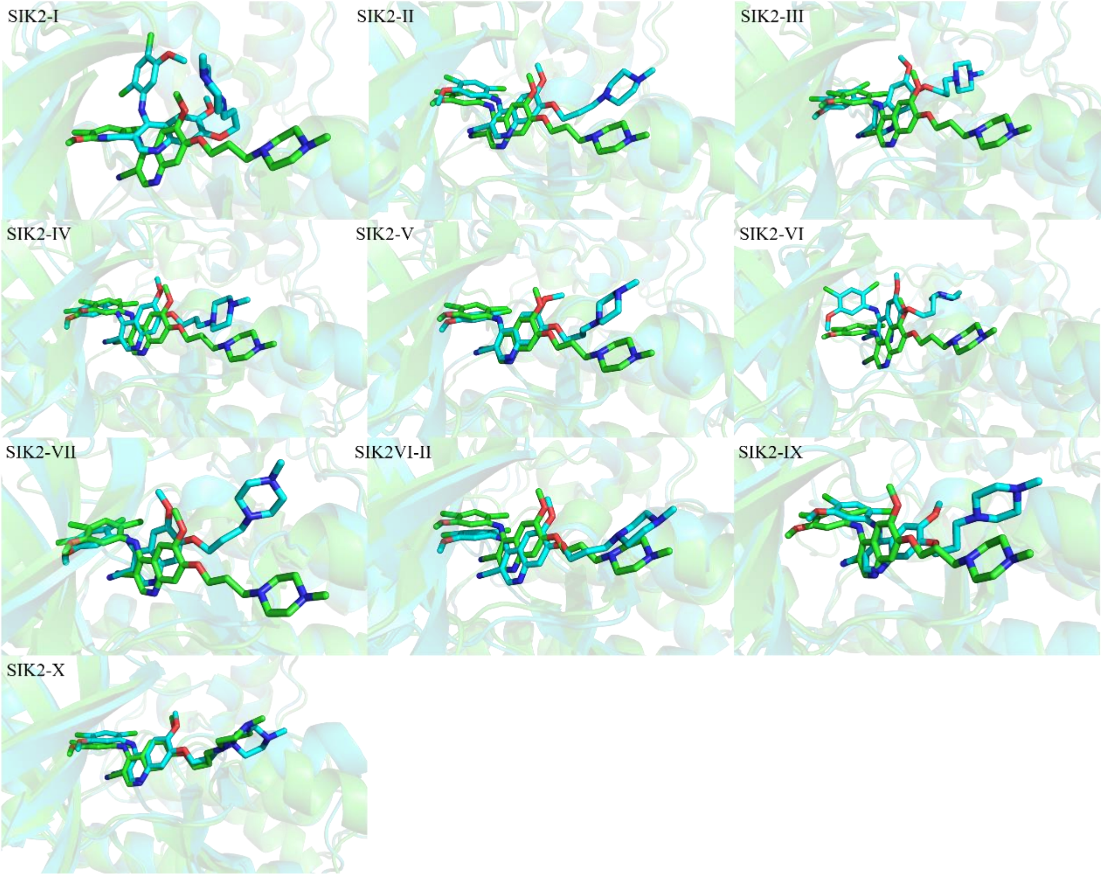

Figure S26. Initial structure and 500th ns frame aligned for bosutinib/SIK2 complex systems.

Green for initial conformation and cyan for 500th ns conformation.

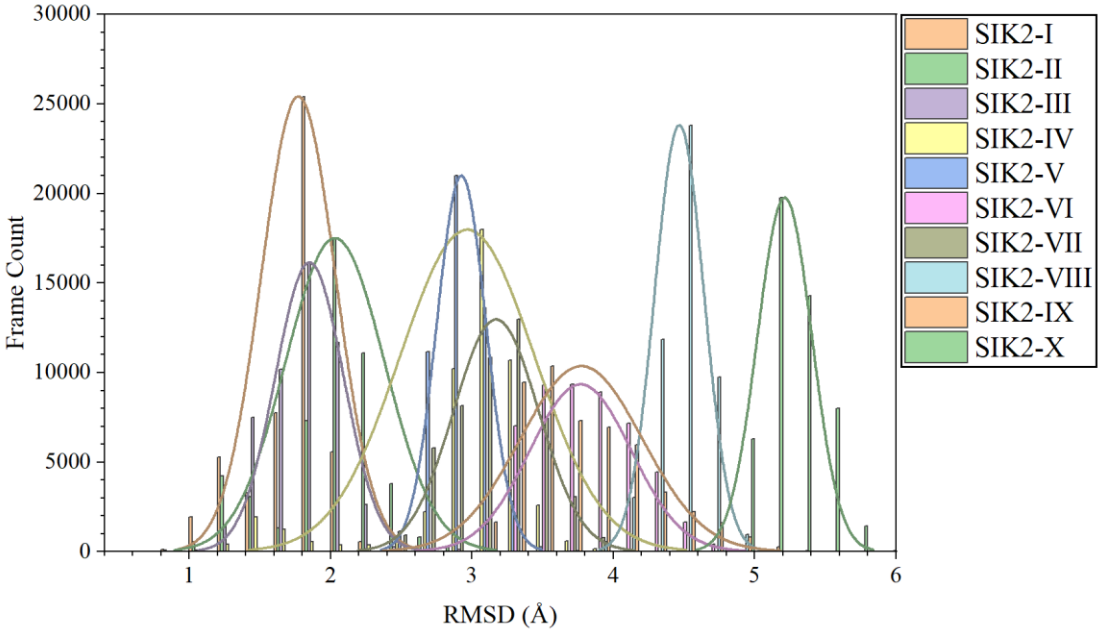

Figure S27. Root mean square deviation (RMSD) values for the heavy atoms of the backbone for kinase domain within the 500 ns MD simulation (total 50000 frames) for bosutinib/SIK2 systems referenced to the initial conformation for SIK2-I.

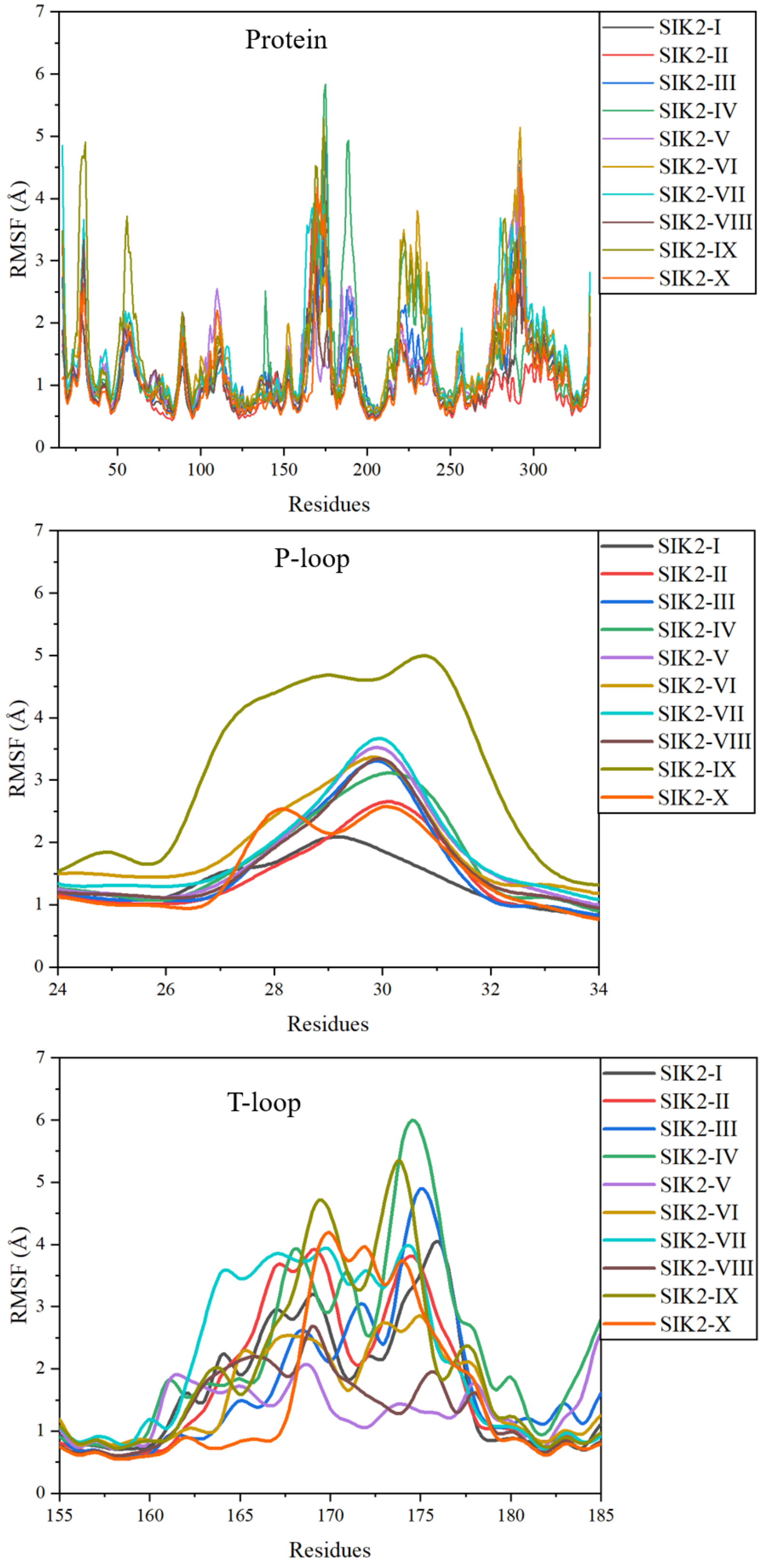

Figure S28. RMSF of the Cα atoms of SIK2 from simulation trajectory of bosutinib/SIK2 systems with 500 ns MD.

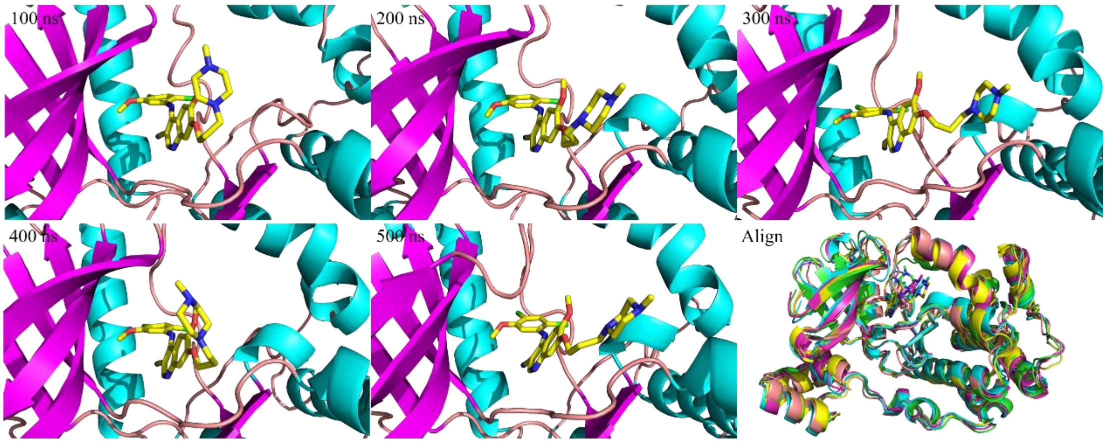

Figure S29. Snapshots of the bosutinib/SIK2-II along the dynamic simulation time for 100, 200, 300, 400, and 500 ns.

For clarity, the water molecules have been removed. The inhibitor is plotted using stick style, while cartoon style for SIK2.

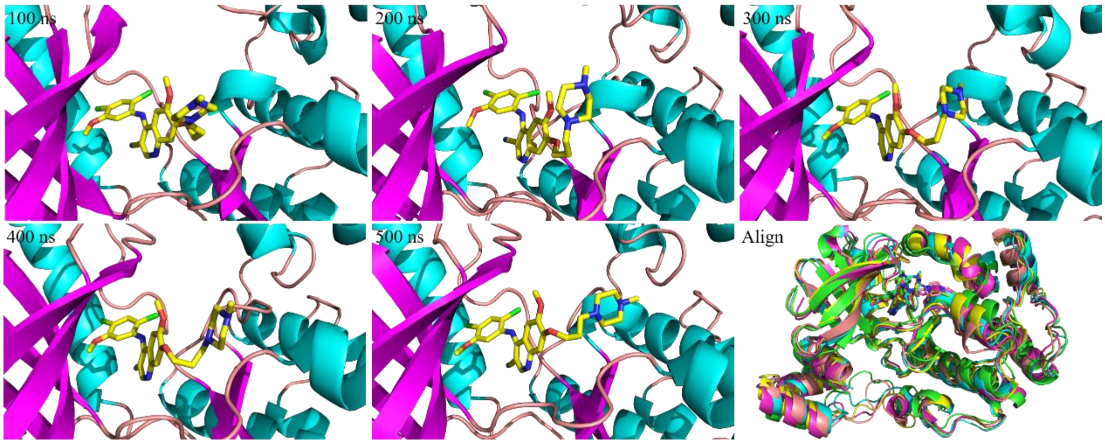

Figure S30. Snapshots of the bosutinib/SIK2-III along the dynamic simulation time for 100, 200, 300, 400, and 500 ns.

For clarity, the water molecules have been removed. The inhibitor is plotted using stick style, while cartoon style for SIK2.

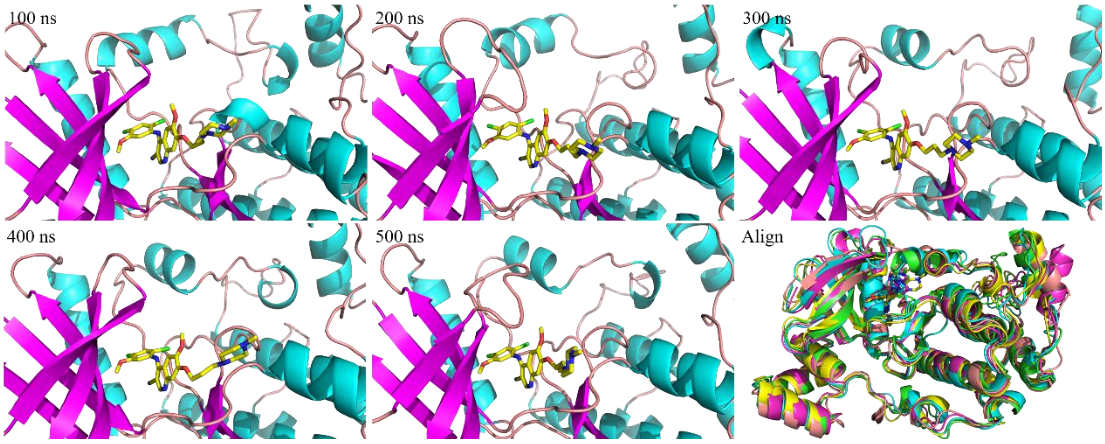

Figure S31. Snapshots of the bosutinib/SIK2-IV along the dynamic simulation time for 100, 200, 300, 400, and 500 ns.

For clarity, the water molecules have been removed. The inhibitor is plotted using stick style, while cartoon style for SIK2.

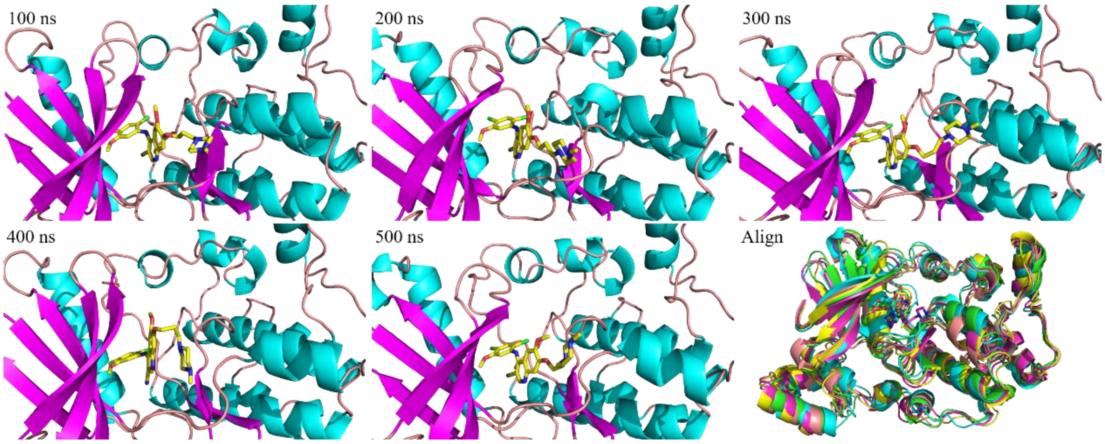

Figure S32. Snapshots of the bosutinib/SIK2-V along the dynamic simulation time for 100, 200, 300, 400, and 500 ns.

For clarity, the water molecules have been removed. The inhibitor is plotted using stick style, while cartoon style for SIK2.

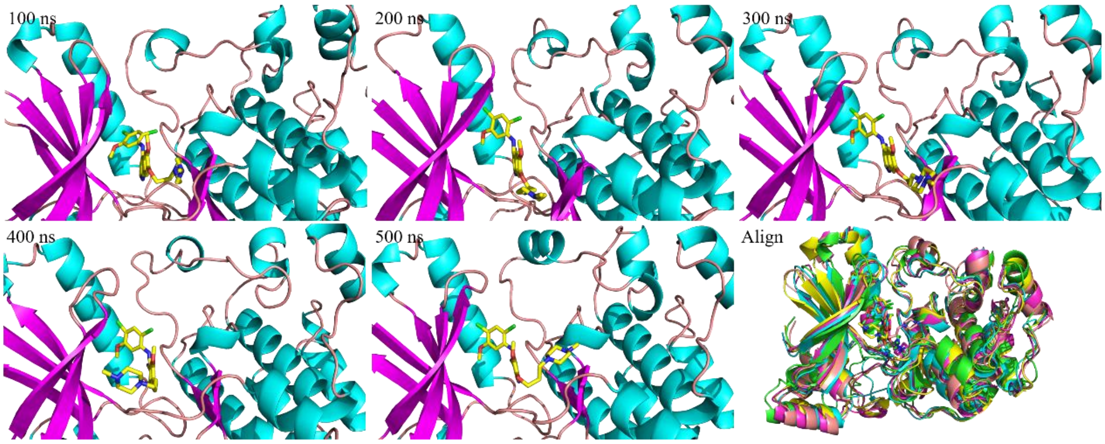

Figure S33. Snapshots of the bosutinib/SIK2-VI along the dynamic simulation time for 100, 200, 300, 400, and 500 ns.

For clarity, the water molecules have been removed. The inhibitor is plotted using stick style, while cartoon style for SIK2.

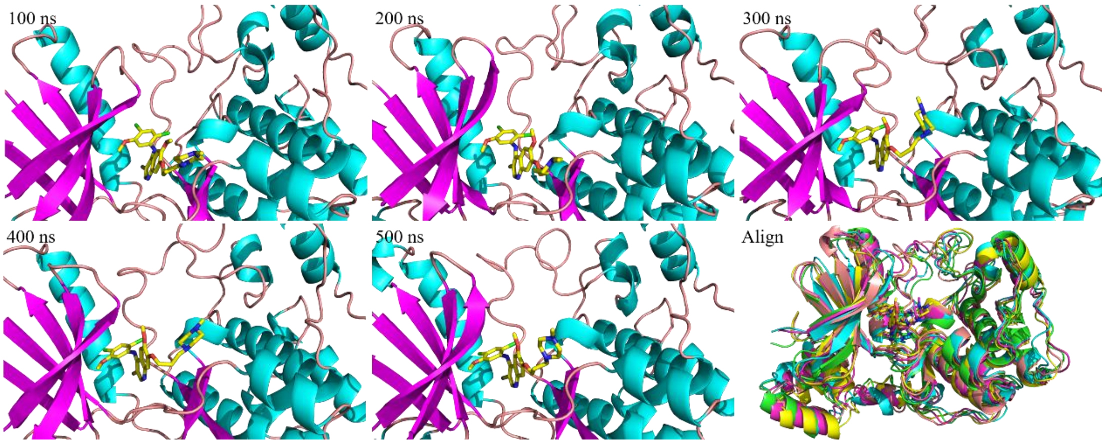

Figure S34. Snapshots of the bosutinib/SIK2-VII along the dynamic simulation time for 100, 200, 300, 400, and 500 ns.

For clarity, the water molecules have been removed. The inhibitor is plotted using stick style, while cartoon style for SIK2.

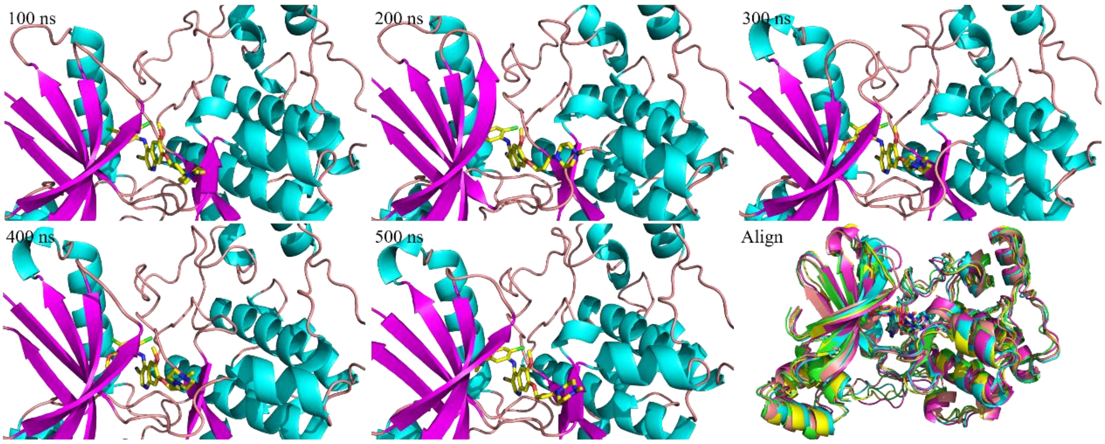

Figure S35. Snapshots of the bosutinib/SIK2-VIII along the dynamic simulation time for 100, 200, 300, 400, and 500 ns.

For clarity, the water molecules have been removed. The inhibitor is plotted using stick style, while cartoon style for SIK2.

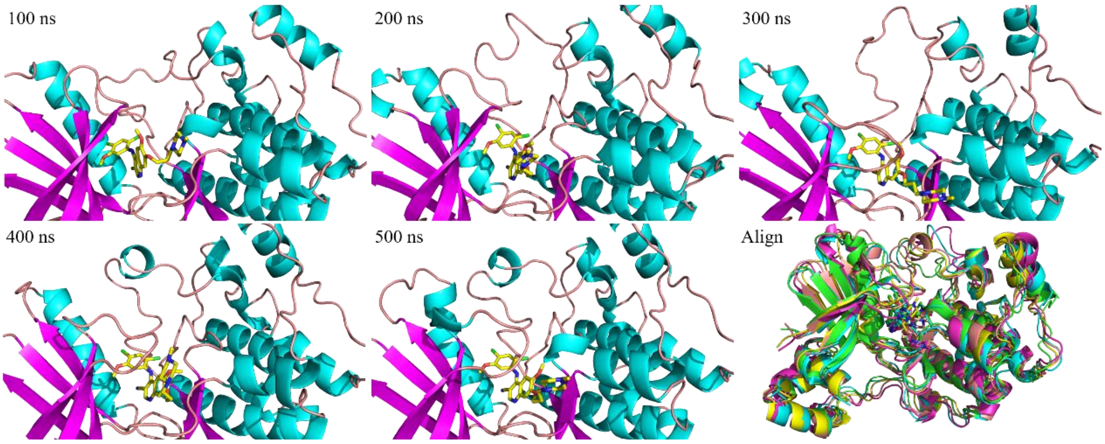

Figure S36. Snapshots of the bosutinib/SIK2-IX along the dynamic simulation time for 100, 200, 300, 400, and 500 ns.

For clarity, the water molecules have been removed. The inhibitor is plotted using stick style, while cartoon style for SIK2.

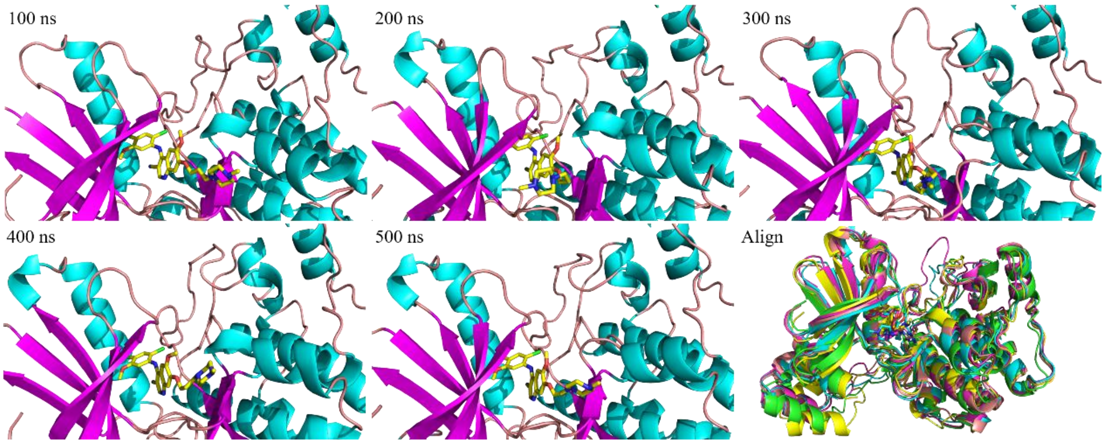

Figure S37. Snapshots of the bosutinib/SIK2-X along the dynamic simulation time for 100, 200, 300, 400, and 500 ns.

For clarity, the water molecules have been removed. The inhibitor is plotted using stick style, while cartoon style for SIK2.

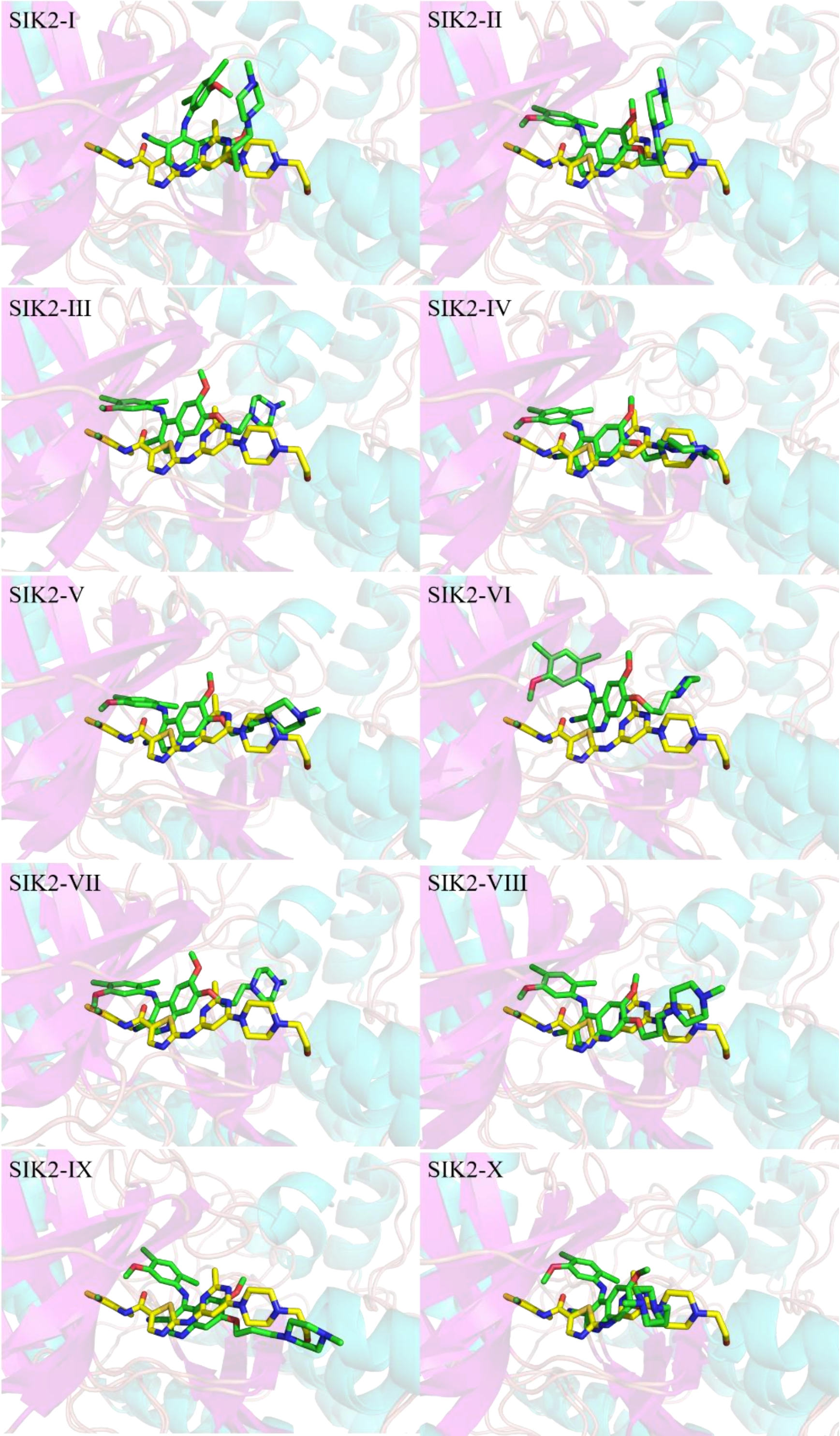

Figure S38. Superimposition structure for dasatinib/SIK2 and bosutinib/SIK2.

The dasatinib/SIK2 complex from our previous work (Shi, M.; Wang, L.; Li, P.; Liu, J.; Chen, L.; Xu, D., Dasatinib-SIK2 Binding Elucidated by Homology Modeling, Molecular Docking, and Dynamics Simulations. ACS Omega 2021, 6 (16), 11025-11038.) and shown yellow in those figures. The bosutinib/SIK2 complex from every system in this work.

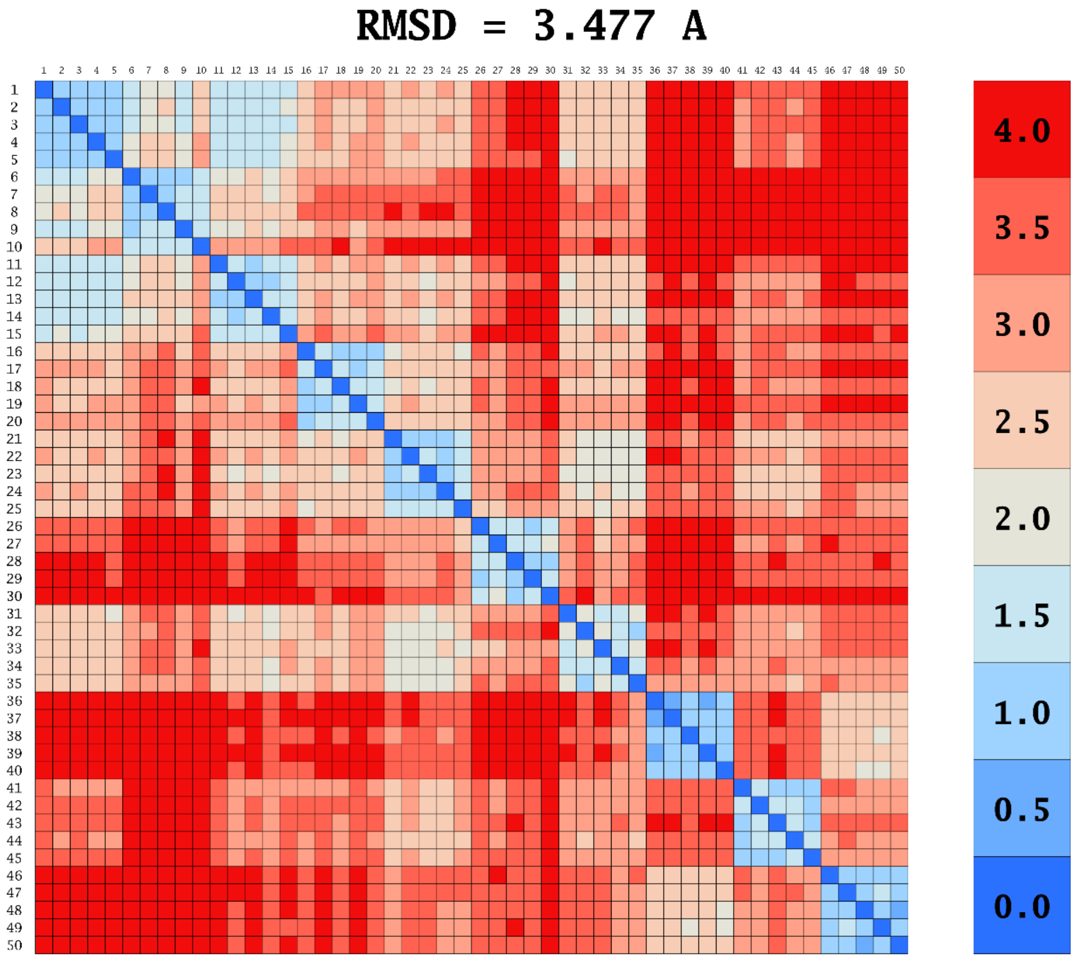

Figure S39. Root mean square deviation (RMSD) for the five representative frames from the ten bosutinib/SIK2 complex systems.

The 1-50 frames were for SIK2-I-1, SIK2-I-2, SIK2-I-3, SIK2-I-4, SIK2-I-5, SIK2-II-1, SIK2-II-2, SIK2-II-3, SIK2-II-4, SIK2-II-5, SIK2-III-1, SIK2-III-2, SIK2-III-3, SIK2-III-4, SIK2-III-5, SIK2-IV-1, SIK2-IV-2, SIK2-IV-3, SIK2-IV-4, SIK2-IV-5, SIK2-V-1, SIK2-V-2, SIK2-V-3, SIK2-V-4, SIK2-V-5, SIK2-VI-1, SIK2-VI-2, SIK2-VI-3, SIK2-VI-4, SIK2-VI-5, SIK2-VII-1, SIK2-VII-2, SIK2-VII-3, SIK2-VII-4, SIK2-VII-5, SIK2-VIII-1, SIK2-VIII-2, SIK2-VIII-3, SIK2-VIII-4, SIK2-VIII-5, SIK2-IX-1, SIK2-IX-2, SIK2-IX-3, SIK2-IX-4, SIK2-IX-5, SIK2-X-1, SIK2-X-2, SIK2-X-3, SIK2-X-4, SIK2-X-5 frames.

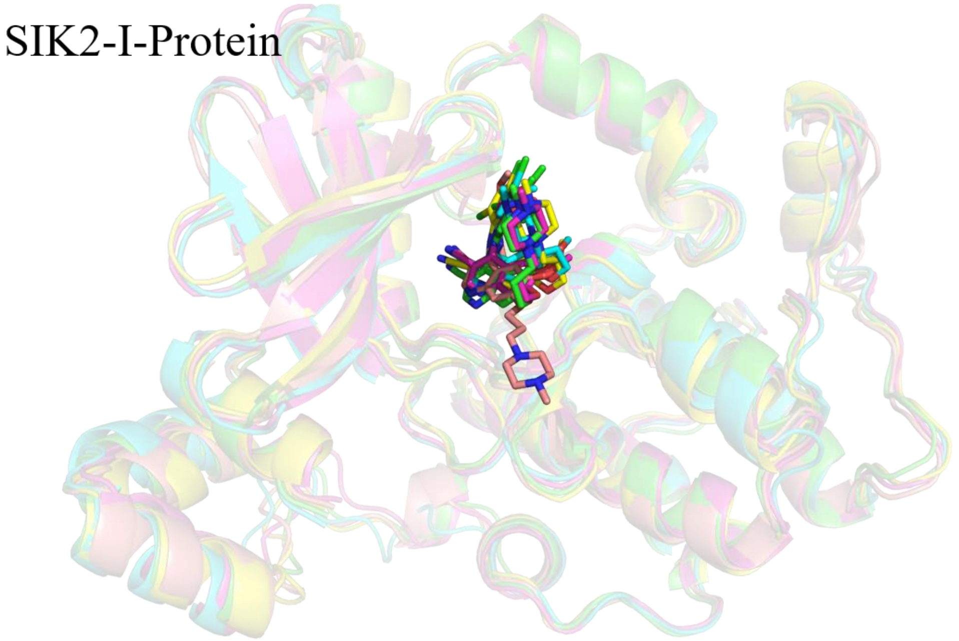

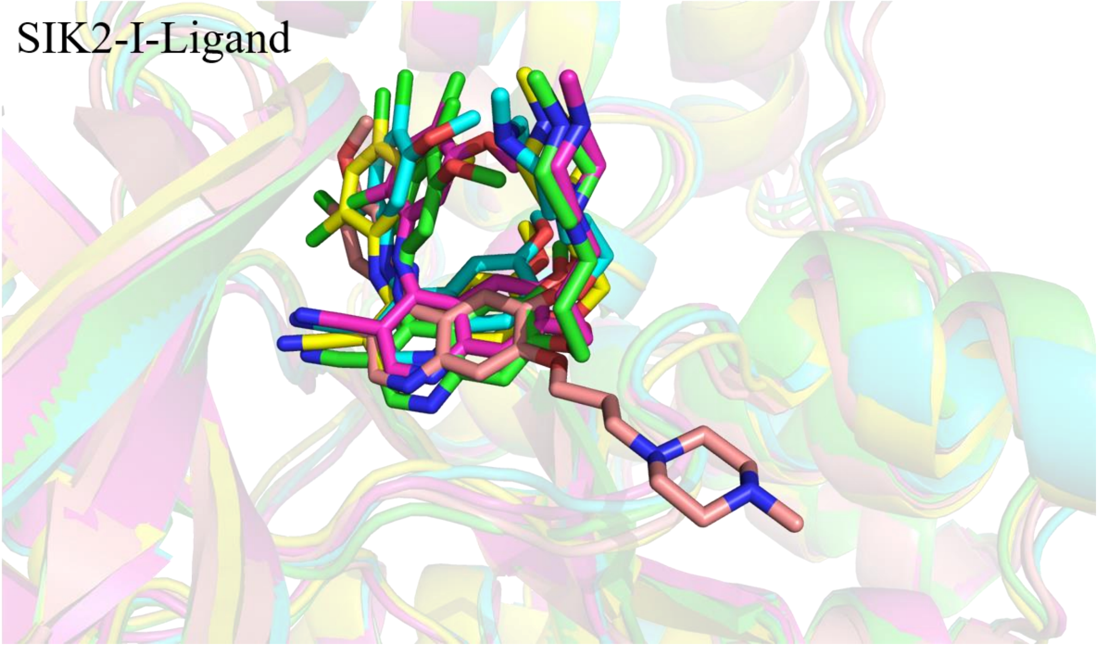

Figure S40. Five representative frames from the cluster analysis for bosutinib/SIK2-I systems.

For clarity, the water molecules have been removed. The inhibitor bosutinib is plotted using stick style, while cartoon style for SIK2.

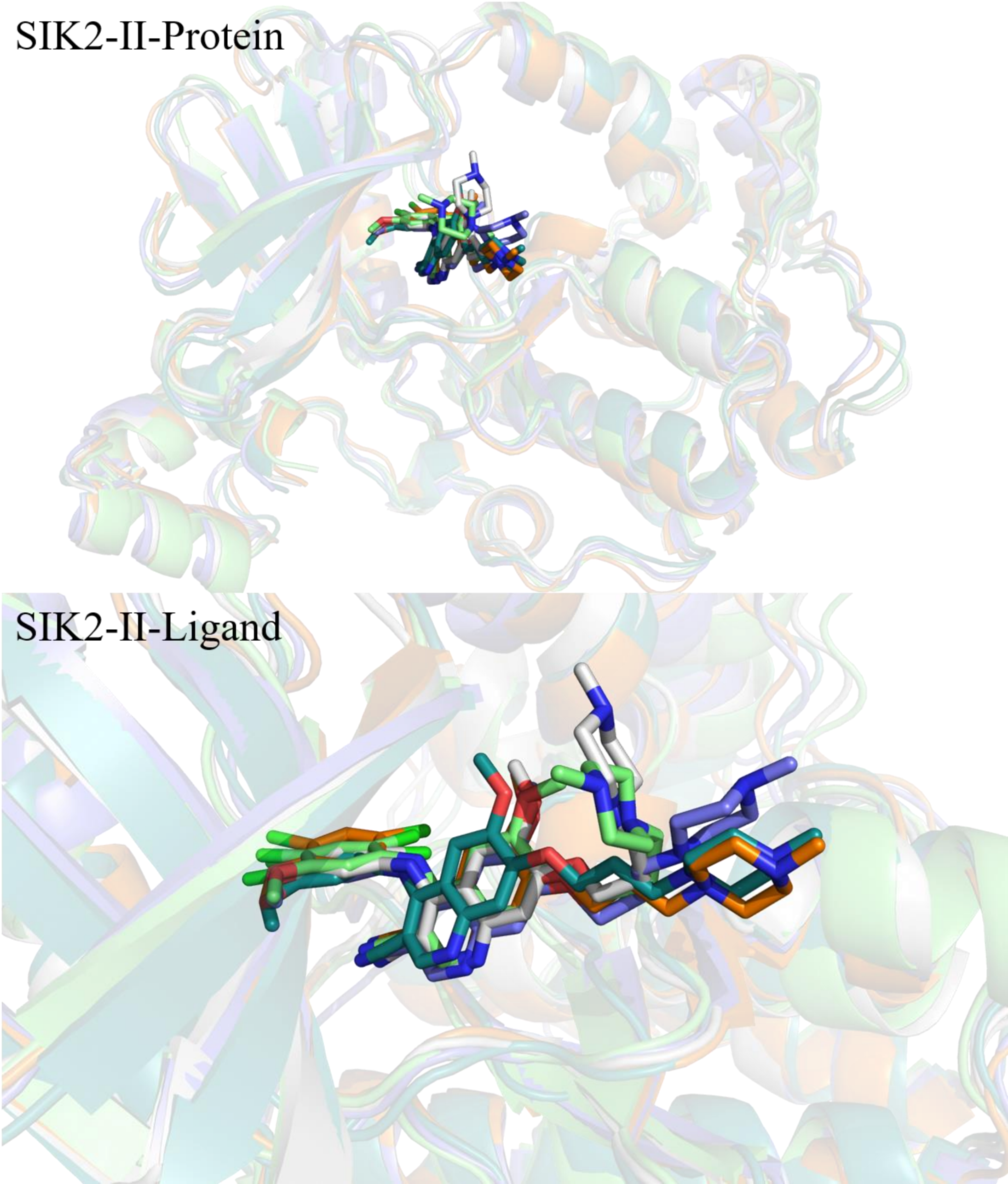

Figure S41. Five representative frames from the cluster analysis for bosutinib/SIK2-II systems.

For clarity, the water molecules have been removed. The inhibitor bosutinib is plotted using stick style, while cartoon style for SIK2.

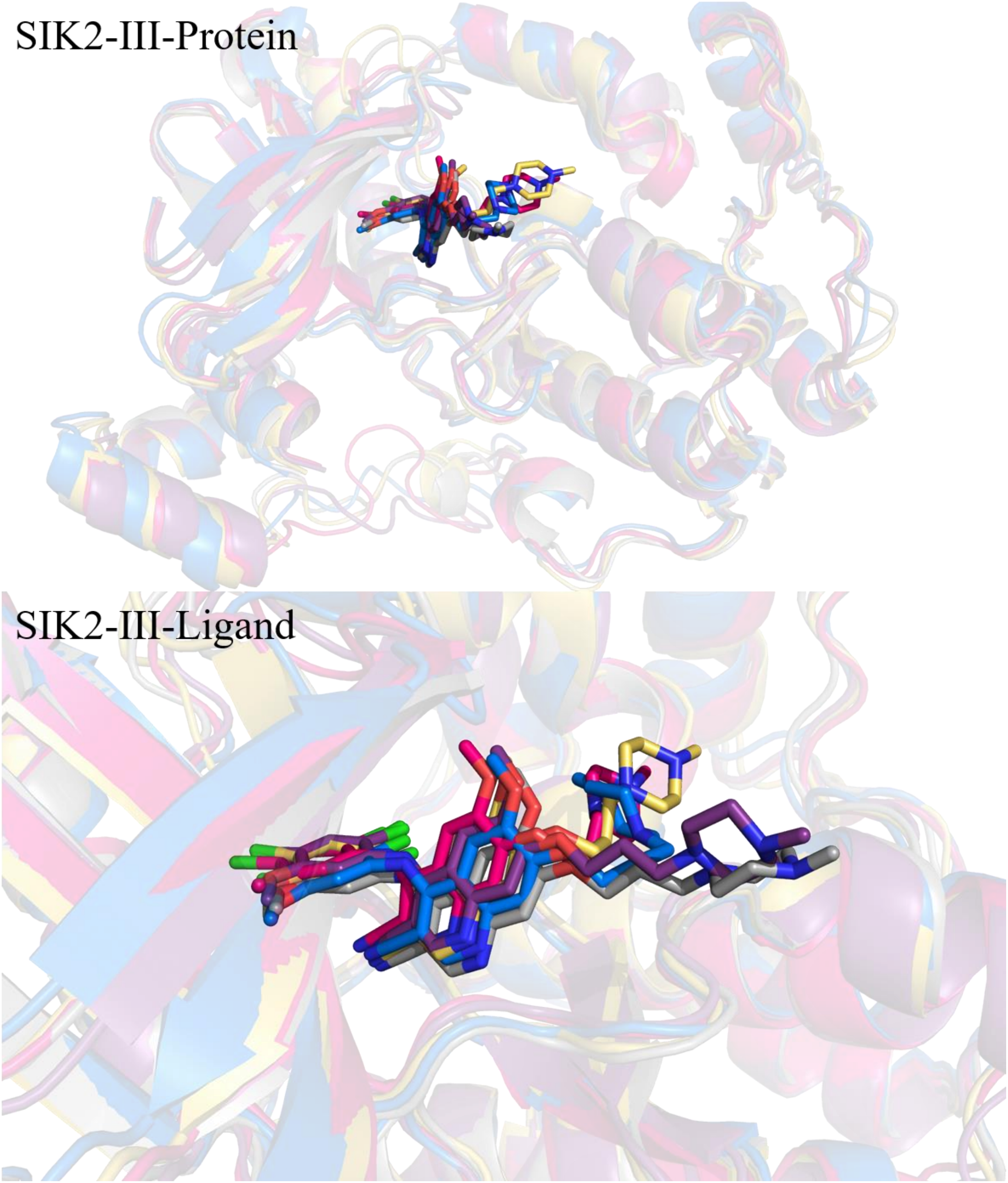

Figure S42. Five representative frames from the cluster analysis for bosutinib/SIK2-III systems.

For clarity, the water molecules have been removed. The inhibitor bosutinib is plotted using stick style, while cartoon style for SIK2.

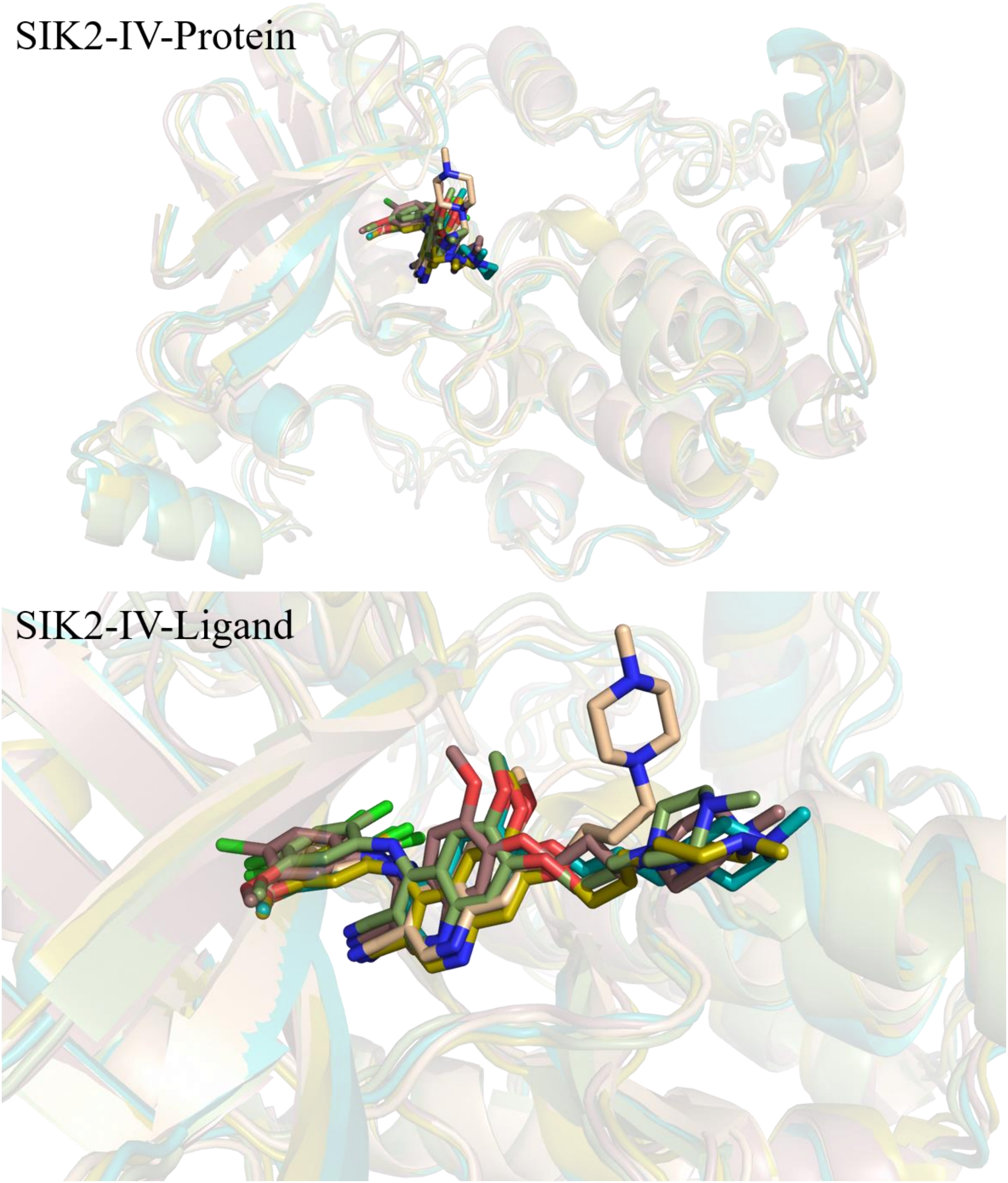

Figure S43. Five representative frames from the cluster analysis for bosutinib/SIK2-IV systems.

For clarity, the water molecules have been removed. The inhibitor bosutinib is plotted using stick style, while cartoon style for SIK2.

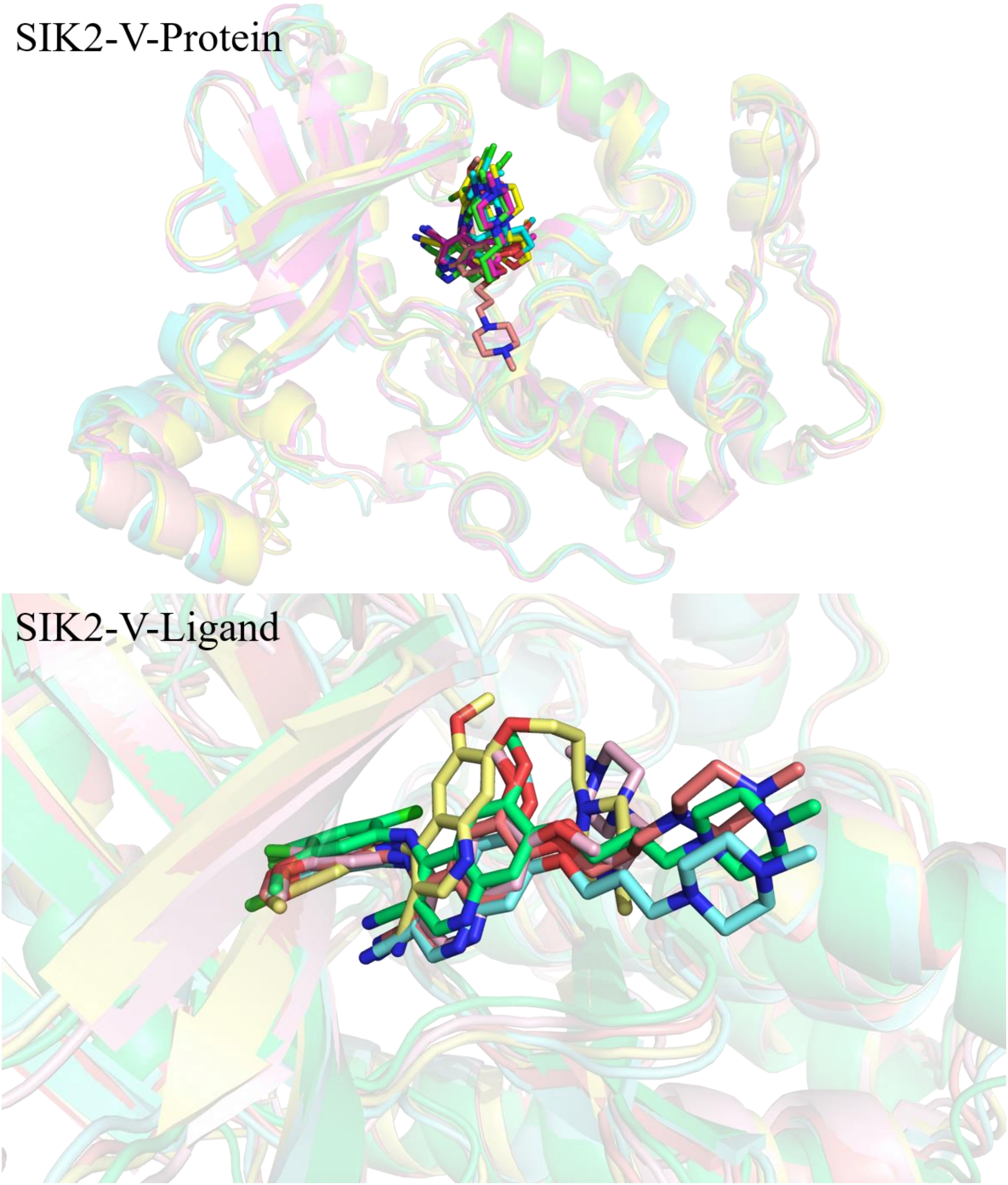

Figure S44. Five representative frames from the cluster analysis for bosutinib/SIK2-V systems.

For clarity, the water molecules have been removed. The inhibitor bosutinib is plotted using stick style, while cartoon style for SIK2.

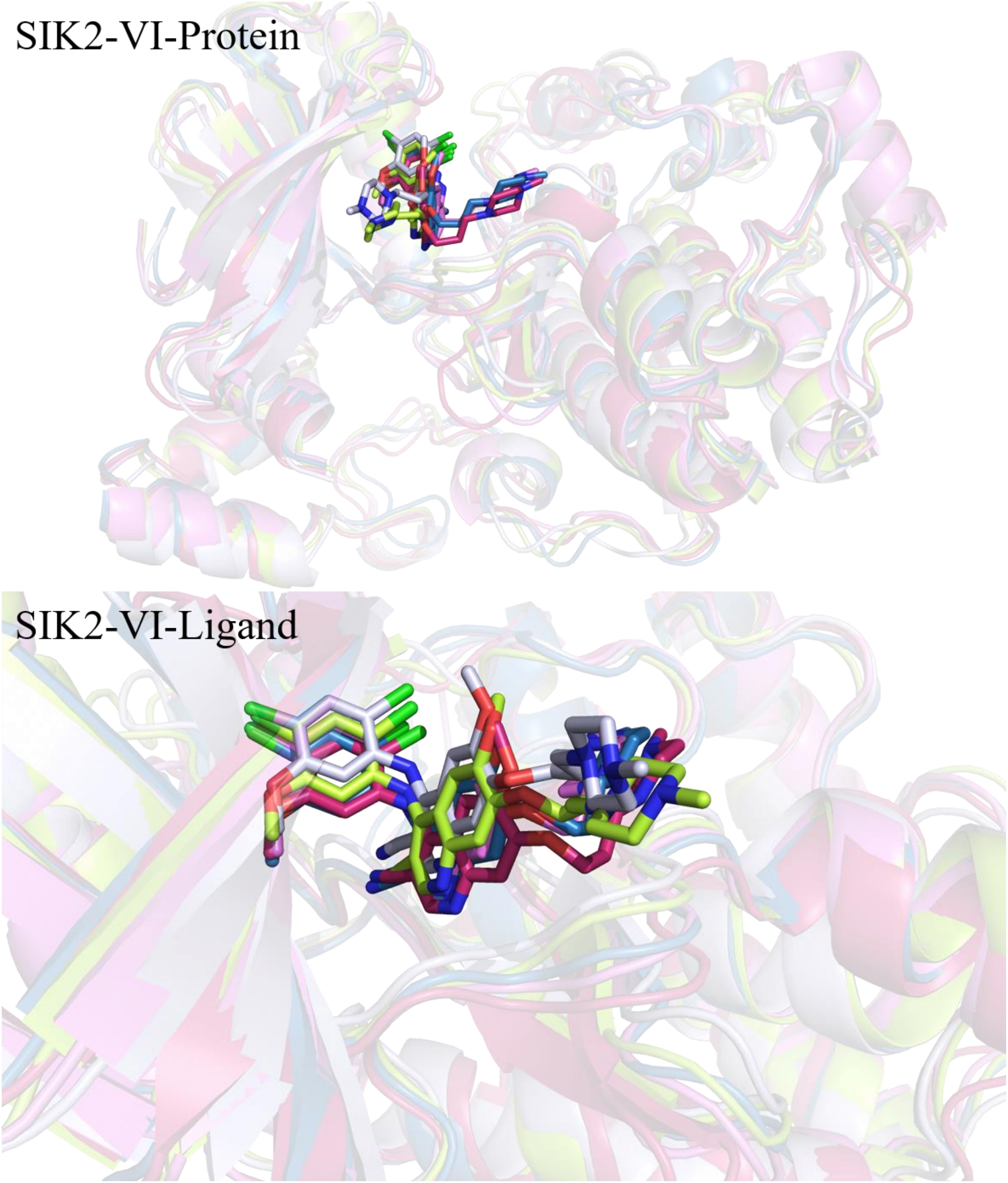

Figure S45. Five representative frames from the cluster analysis for bosutinib/SIK2-VI systems.

For clarity, the water molecules have been removed. The inhibitor bosutinib is plotted using stick style, while cartoon style for SIK2.

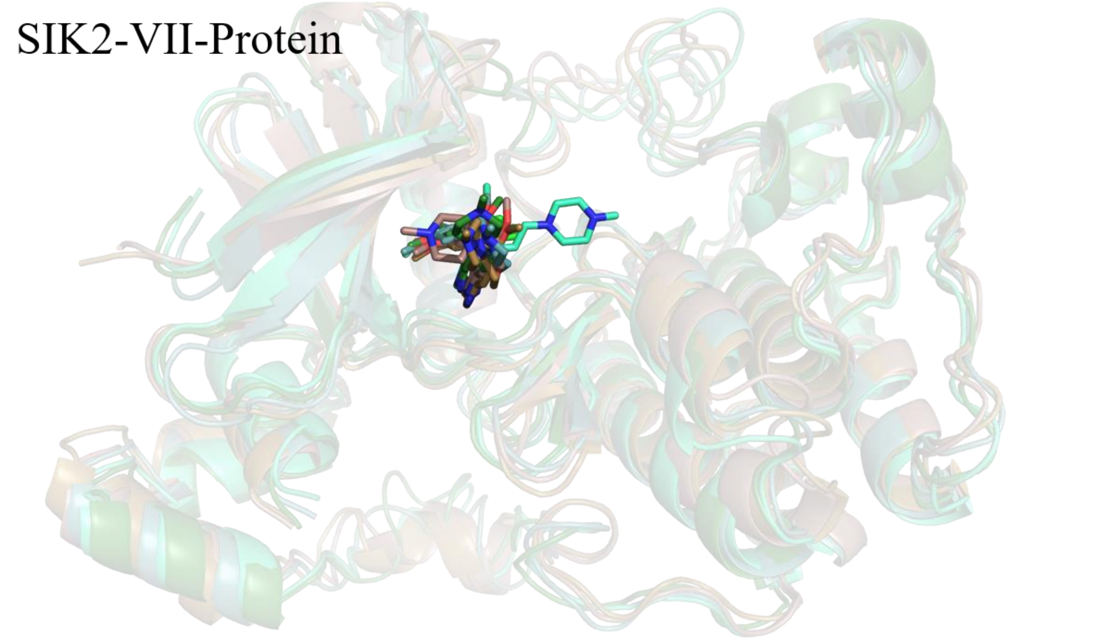

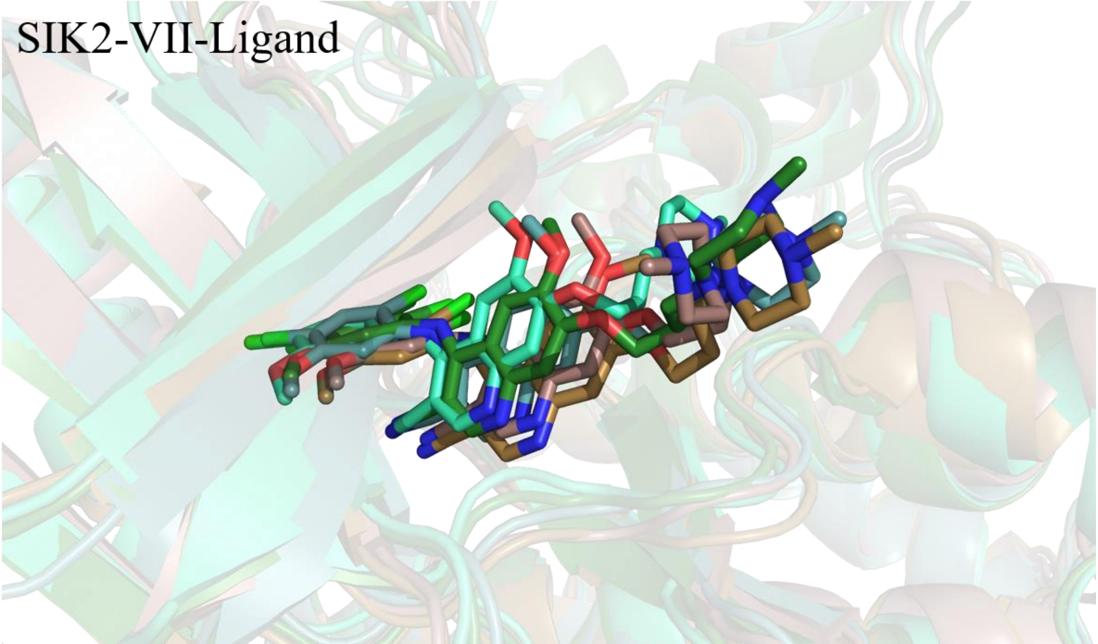

Figure S46. Five representative frames from the cluster analysis for bosutinib/SIK2-VII systems.

For clarity, the water molecules have been removed. The inhibitor bosutinib is plotted using stick style, while cartoon style for SIK2.

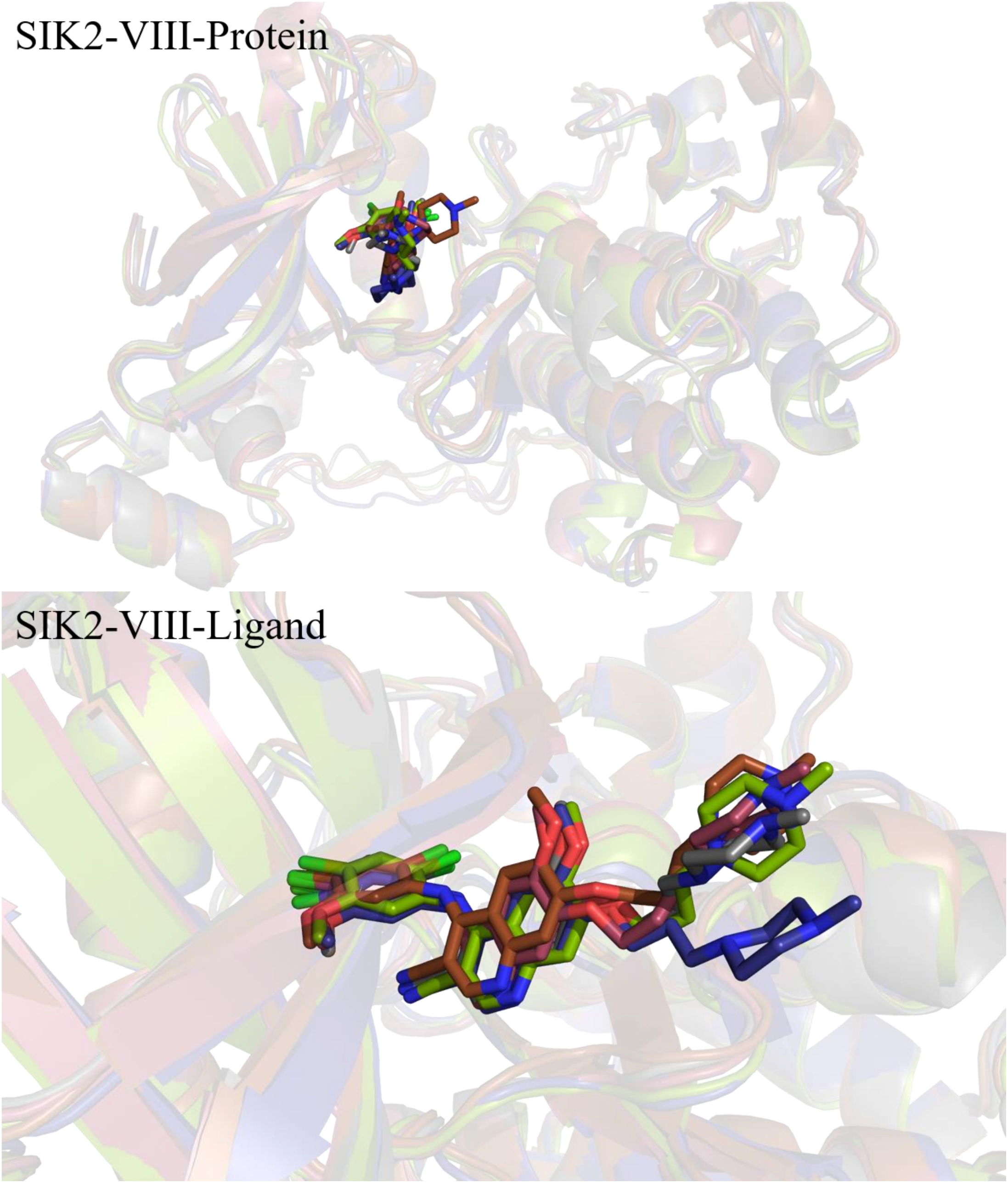

Figure S47. Five representative frames from the cluster analysis for bosutinib/SIK2-VIII systems.

For clarity, the water molecules have been removed. The inhibitor bosutinib is plotted using stick style, while cartoon style for SIK2.

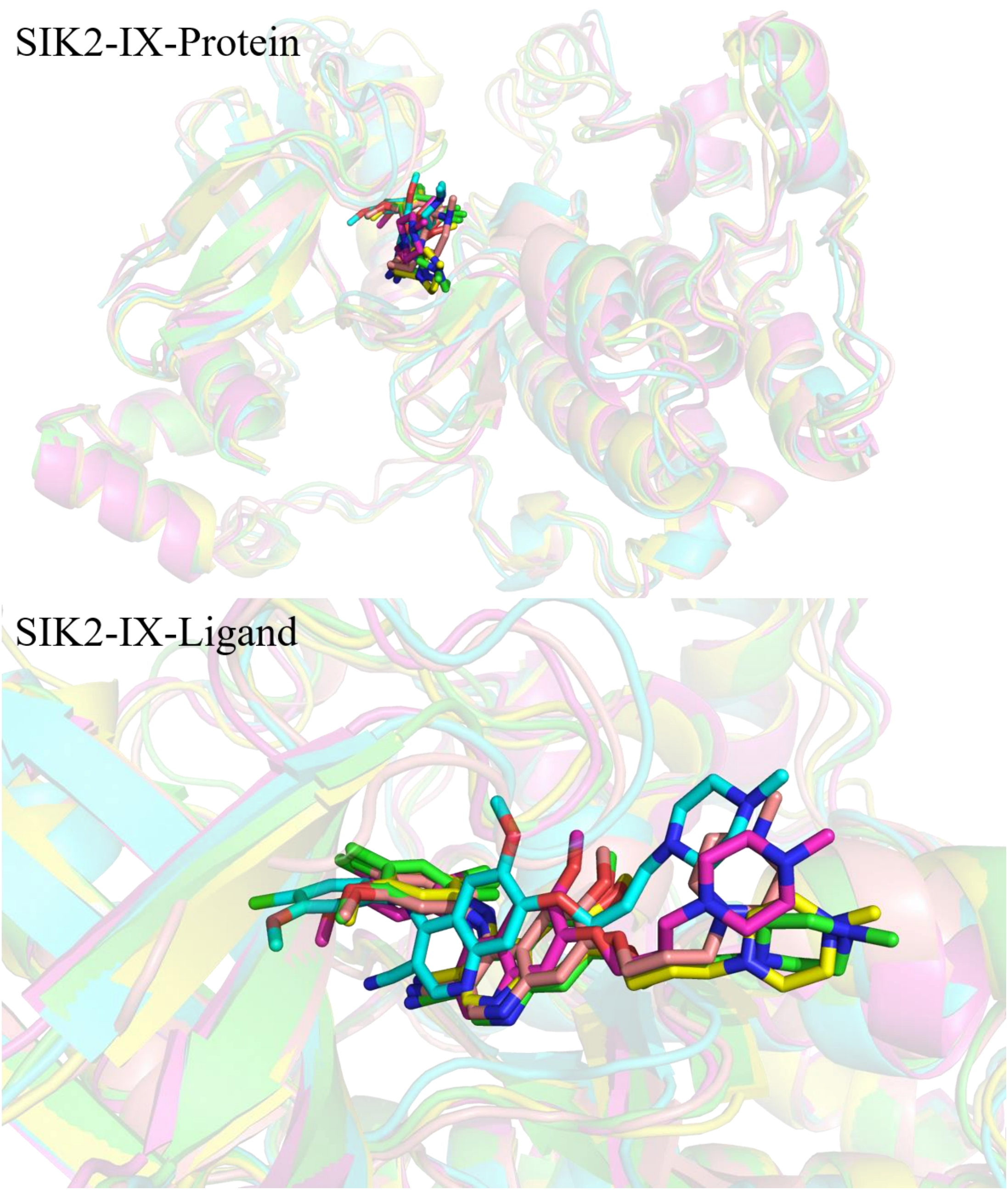

Figure S48. Five representative frames from the cluster analysis for bosutinib/SIK2-IX systems.

For clarity, the water molecules have been removed. The inhibitor bosutinib is plotted using stick style, while cartoon style for SIK2.

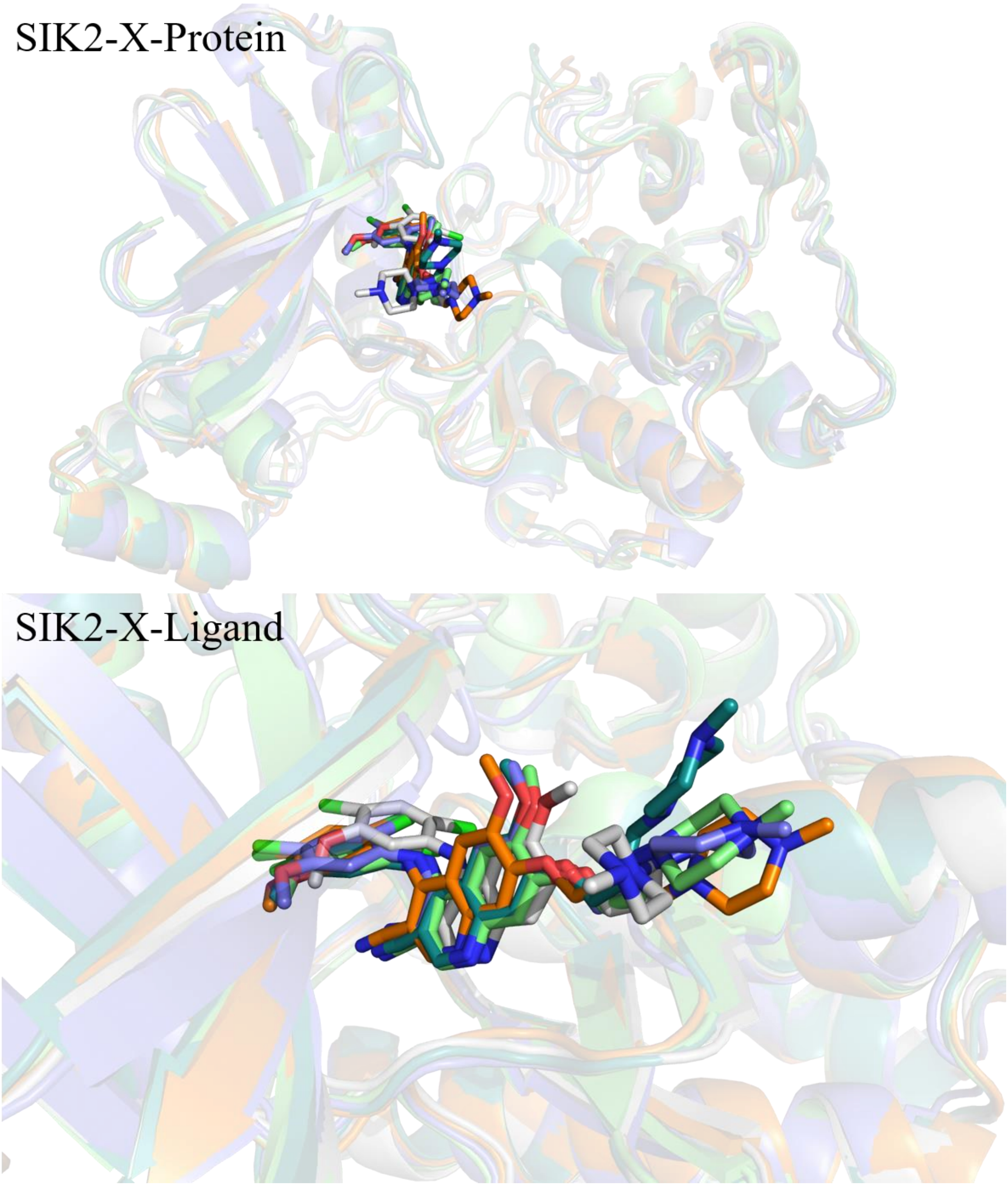

Figure S49. Five representative frames from the cluster analysis for bosutinib/SIK2-X systems.

For clarity, the water molecules have been removed. The inhibitor bosutinib is plotted using stick style, while cartoon style for SIK2.

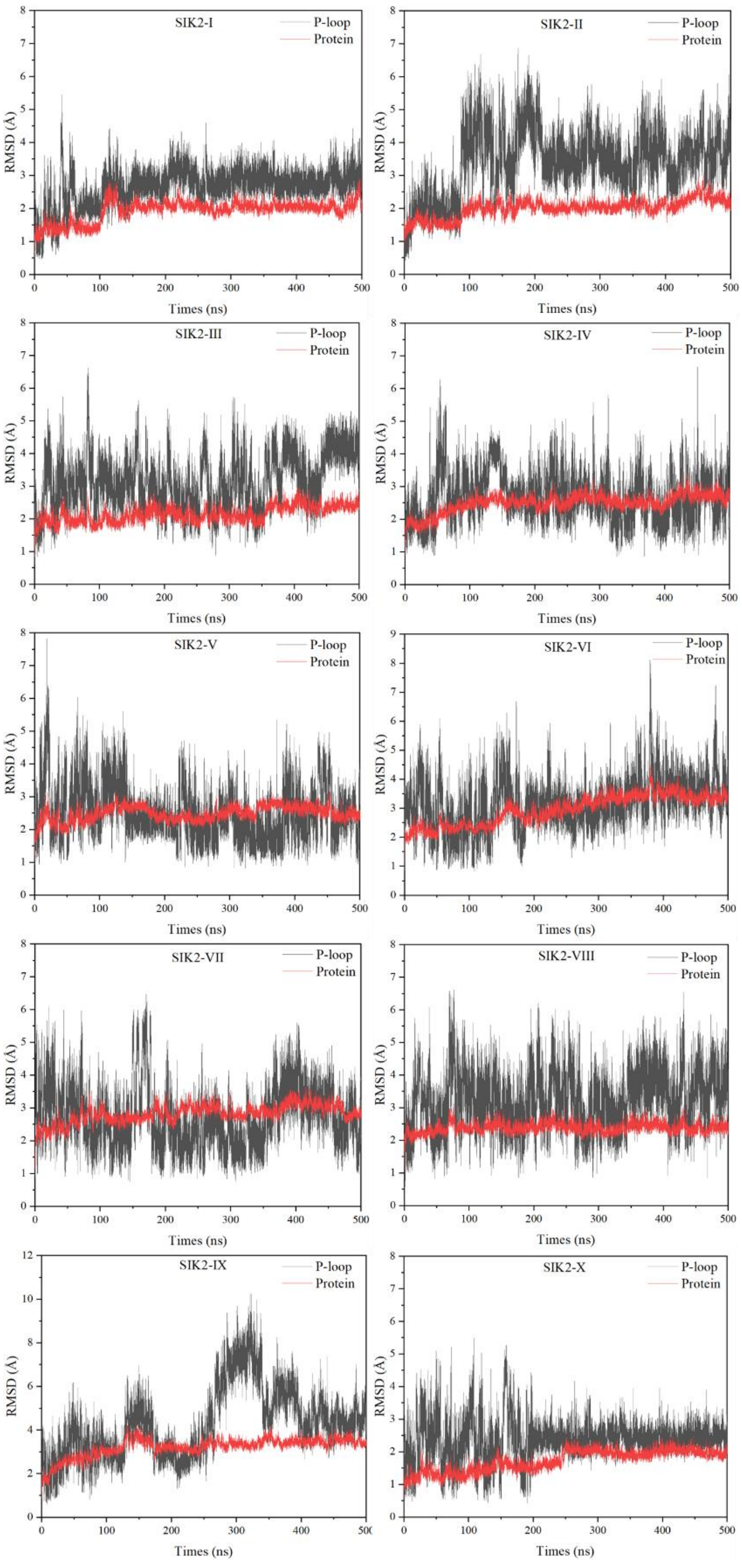

Figure S50. Root mean square deviation (RMSD) value of heavy atoms of backbone of protein and of P-loop along 500 ns MD simulation for bosutinib/SIK2 systems.

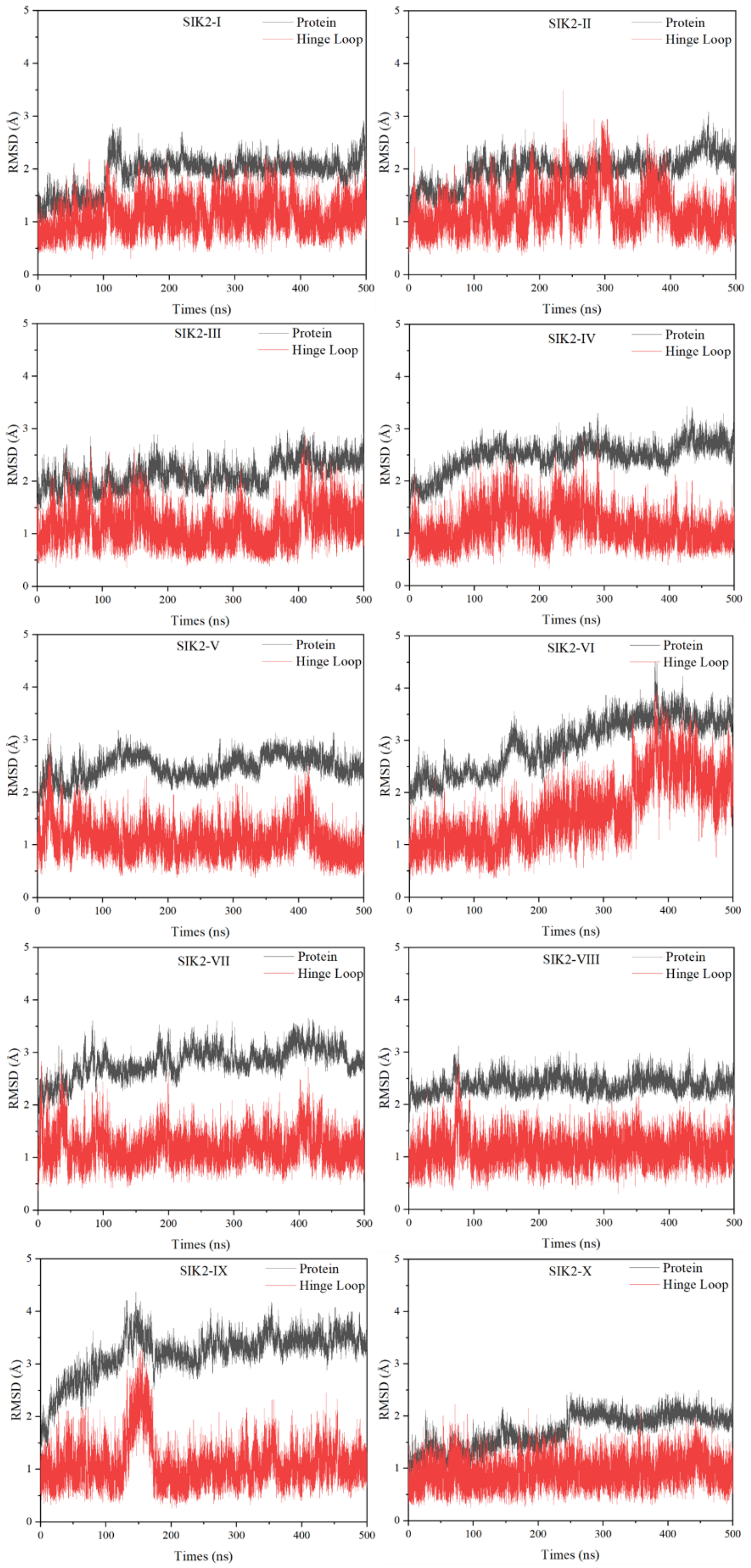

Figure S51. Root mean square deviation (RMSD) value of heavy atoms of backbone of protein and hinge loop of SIK2 along 500 ns MD simulation for bosutinib/SIK2 systems.

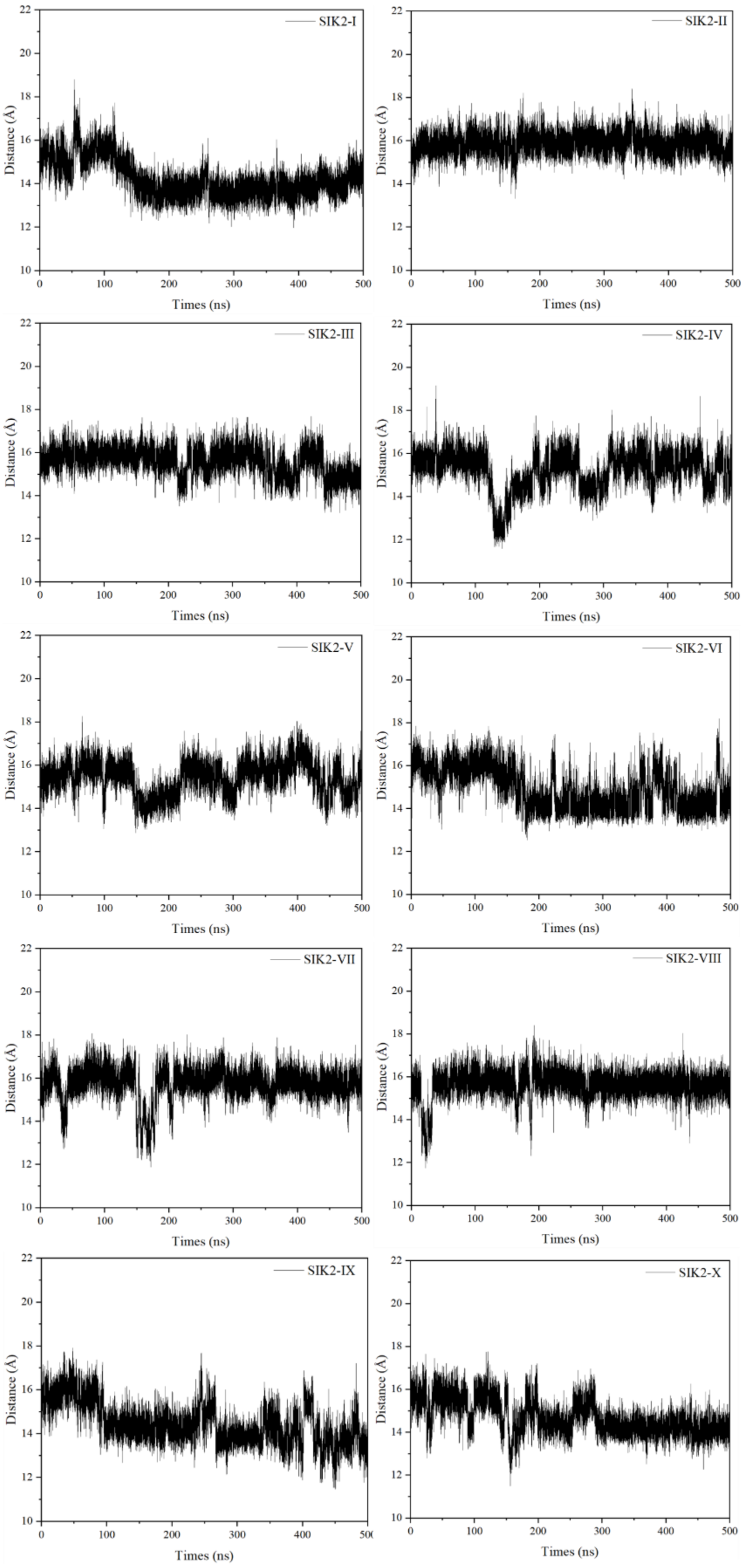

Figure S52. Distance between ^{27}GKG^{29} in P-loop with the ^{97}EYA^{99} in hinge loop although the 500 ns MD simulation.

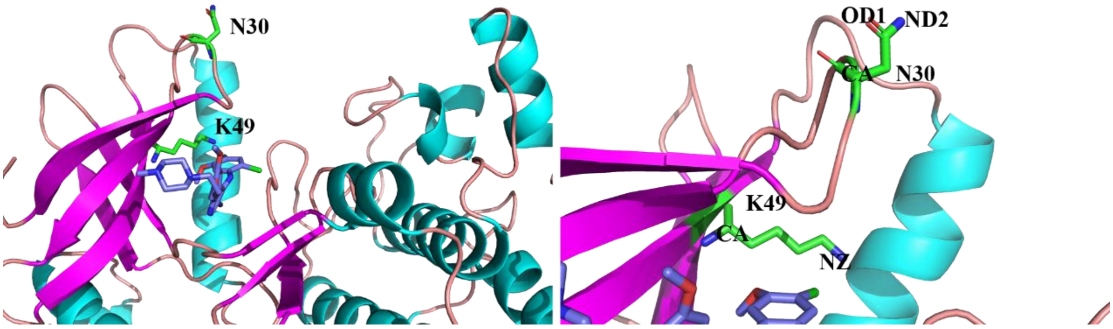

Figure S53. Residues and atoms label for N30 and K49.

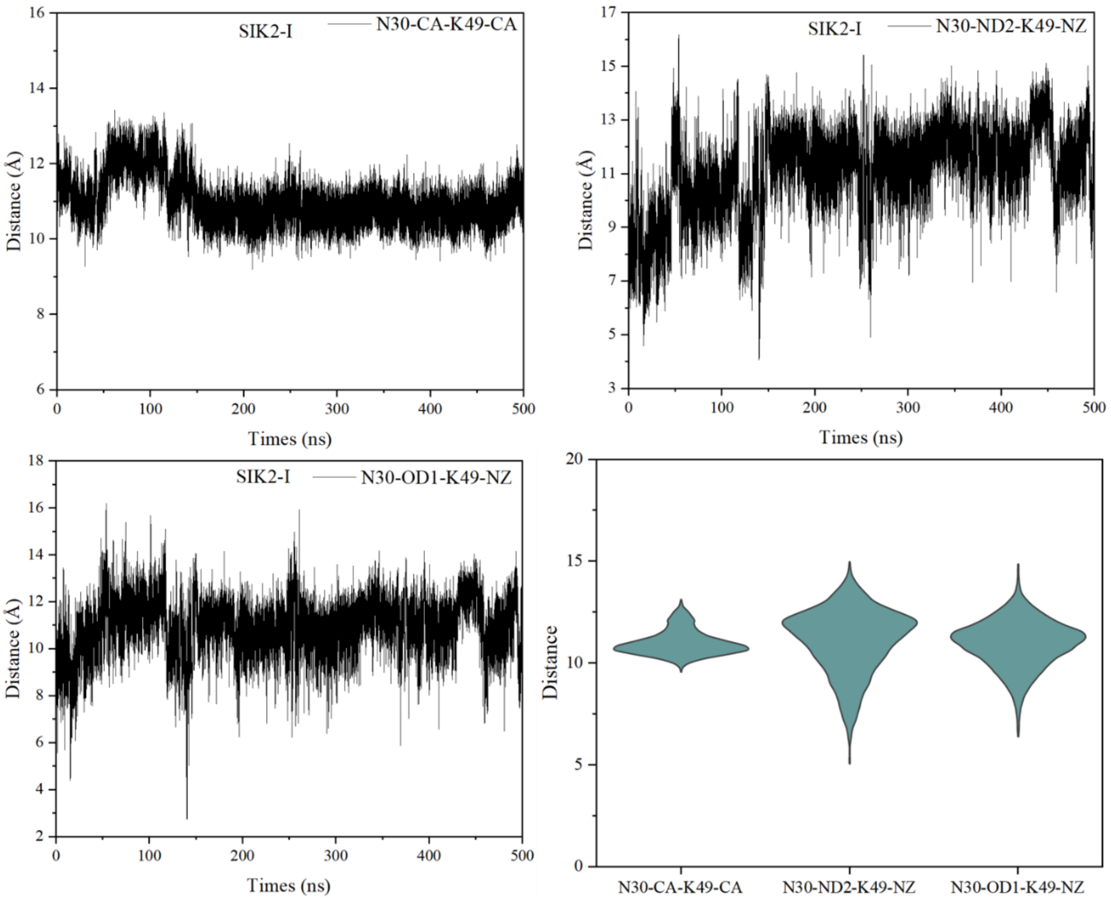

Figure S54. Distance between N30 and K49 for bosutinib/SIK2-I systems.

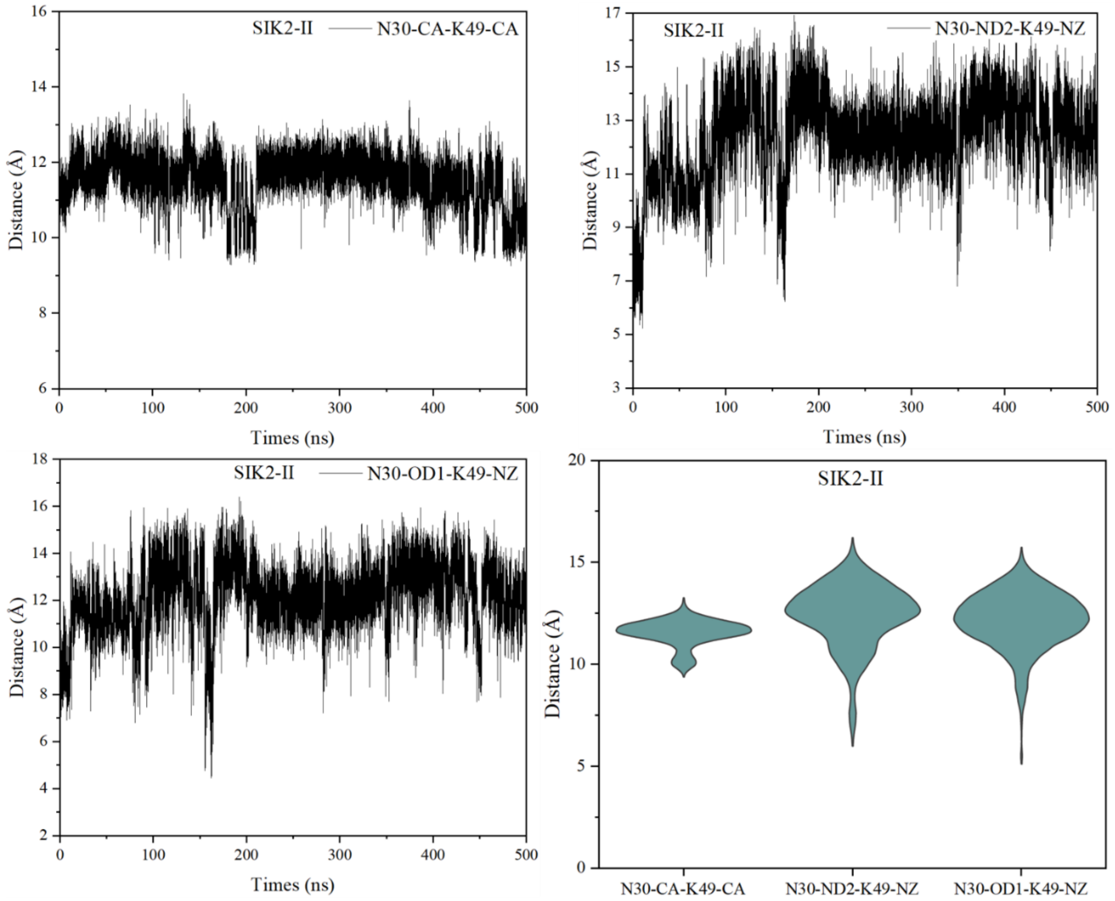

Figure S55. Distance between N30 and K49 for bosutinib/SIK2-II systems.

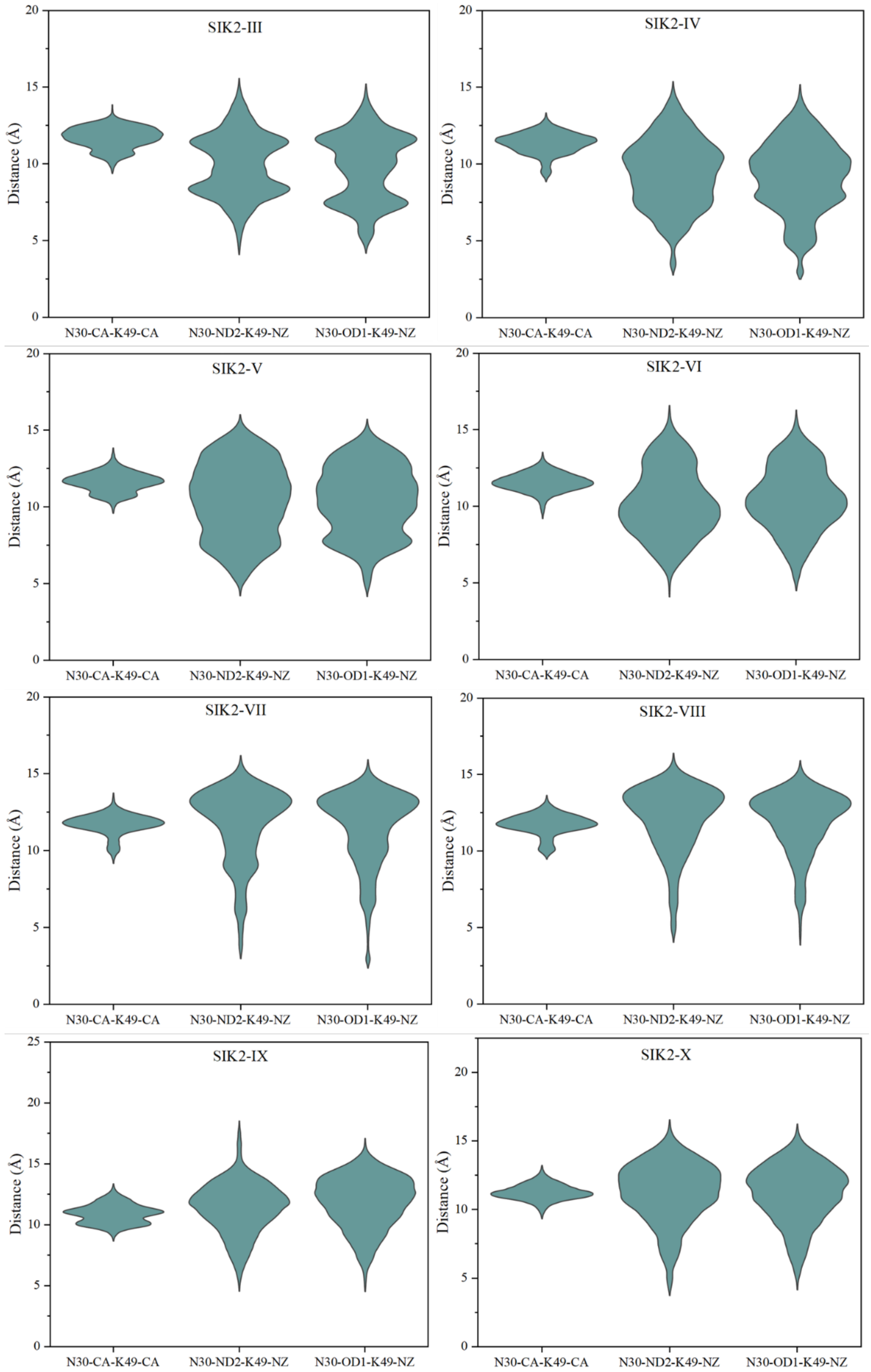

Figure S56. Distance between N30 and K49 for bosutinib/SIK2-III, IV, V, VI, VII, VIII, IX and X systems.

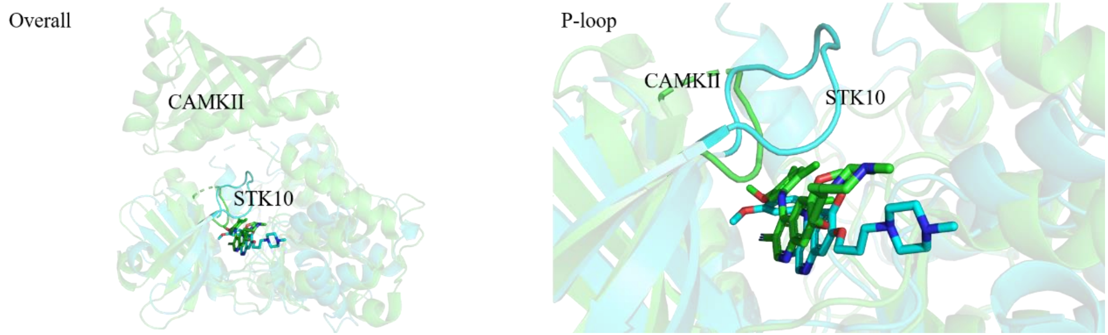

Figure S57. P-loop with down conformation when the bosutinib bound with CAMKII (PDB ID: 3SOA) and STK10 (PDB ID: 5AJQ).

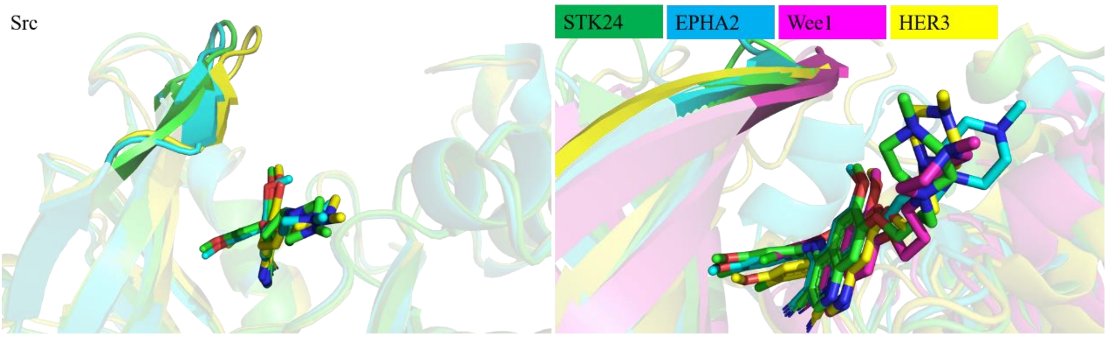

Figure S58. P-loop with up conformation when the bosutinib bound with Src (PDB ID: 4MXO, 4MXX, 4MXY, 4MXZ), STK24 (PDB ID: 4QMN), EHPA2 (PDB ID: 5I9X), Wee1 (PDB ID: 5VC3) and HER3 (PDB ID: 6OP9).

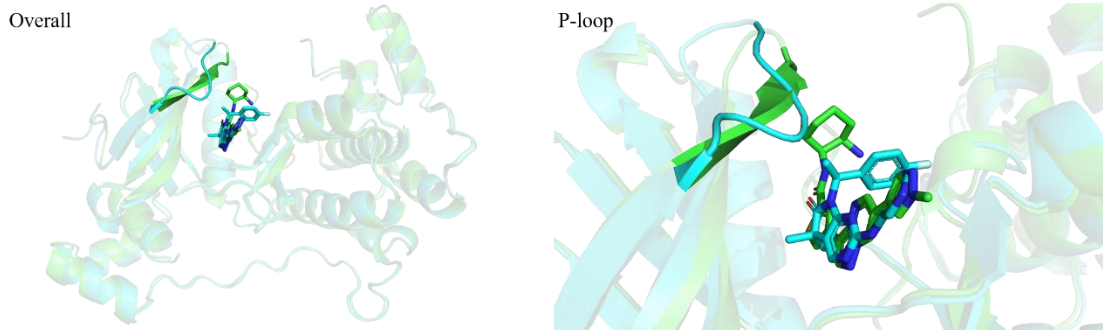

Figure S59. P-loop with up conformation (PDB ID: 5EAK) and down conformation (PDB ID: 5KZ7) for MARK2.

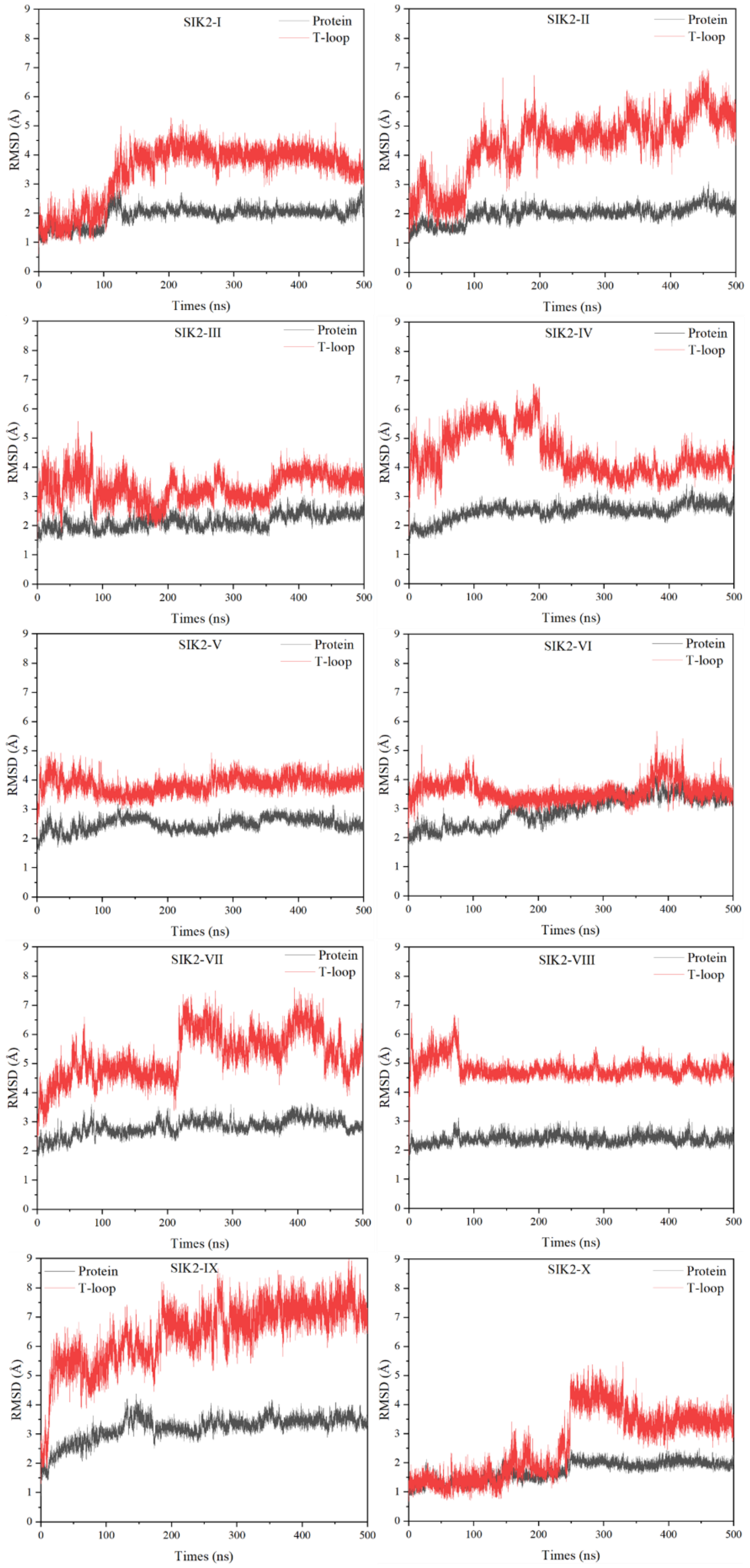

Figure S60. Root mean square deviation (RMSD) value of heavy atoms of backbone of protein and of T-loop along 500 ns MD simulation for bosutinib/SIK2 systems.

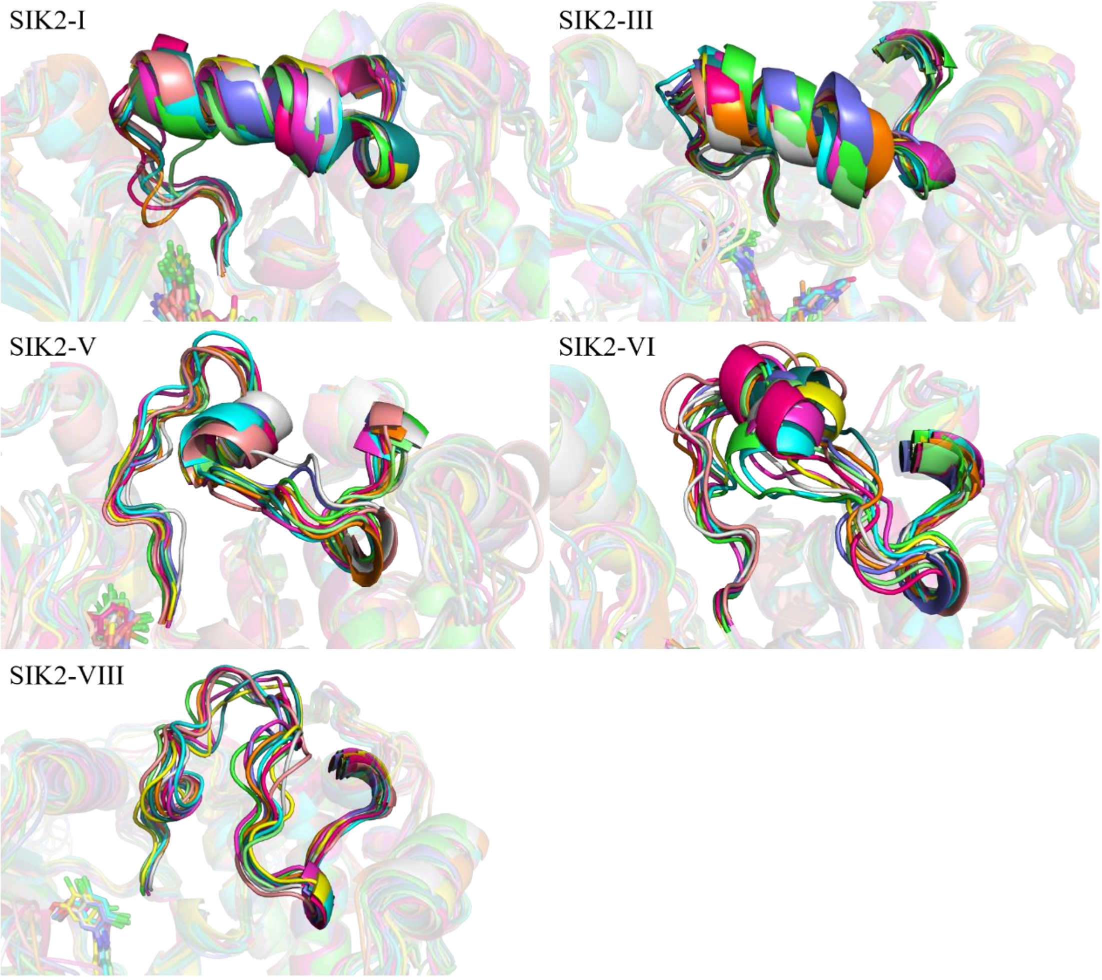

Figure S61. Snapshots of the bosutinib/SIK2-I, III, V, VI, and VIII systems for T-loop conformation along the dynamic simulation time for 300, 320, 340, 360, 380, 400, 420, 440, 460, 480, and 500 ns.

For clarity, the water molecules have been removed. The bosutinib is plotted using stick style, while cartoon style for SIK2.

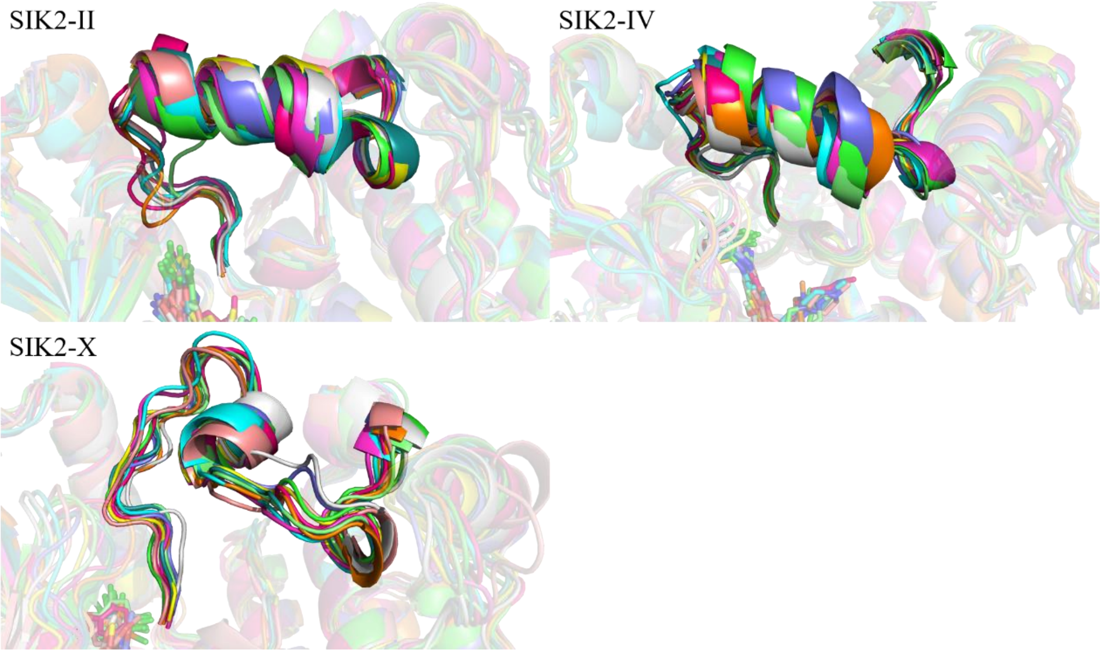

Figure S62. Snapshots of the bosutinib/SIK2-II, IV and X systems for T-loop conformation along the dynamic simulation time for 300, 320, 340, 360, 380, 400, 420, 440, 460, 480, and 500 ns.

For clarity, the water molecules have been removed. The bosutinib is plotted using stick style, while cartoon style for SIK2.

SIK2-VII

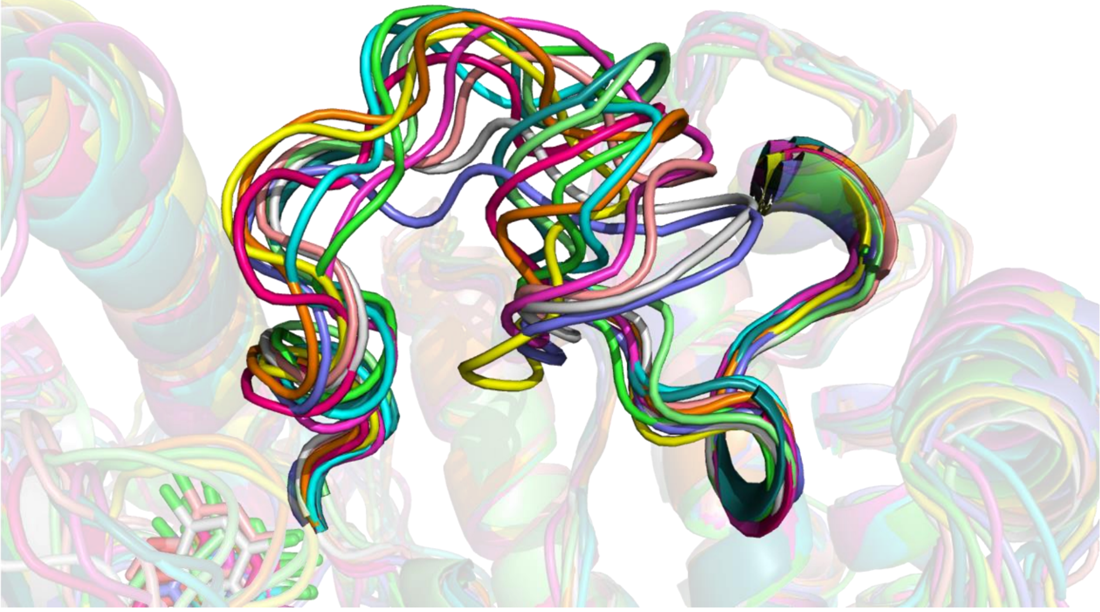

Figure S63. Snapshots of the bosutinib/SIK2-VII system for T-loop conformation along the dynamic simulation time for 300, 320, 340, 360, 380, 400, 420, 440, 460, 480, and 500 ns.

For clarity, the water molecules have been removed. The bosutinib is plotted using stick style, while cartoon style for SIK2.

SIK2-IX

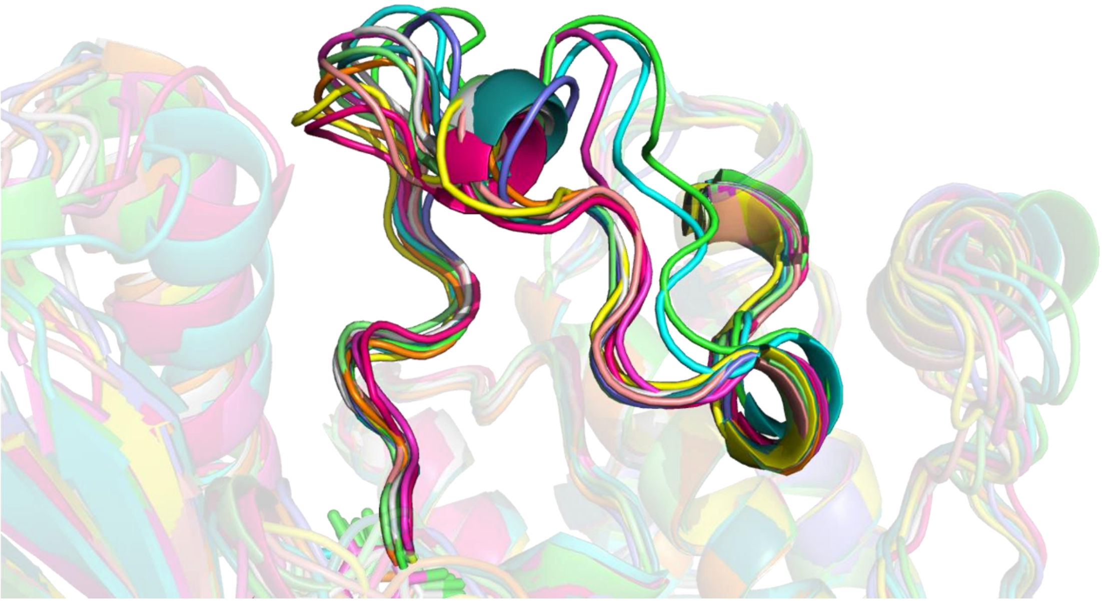

Figure S64. Snapshots of the bosutinib/SIK2-IX system for T-loop conformation along the dynamic simulation time for 300, 320, 340, 360, 380, 400, 420, 440, 460, 480, and 500 ns.

For clarity, the water molecules have been removed. The bosutinib is plotted using stick style, while cartoon style for SIK2.

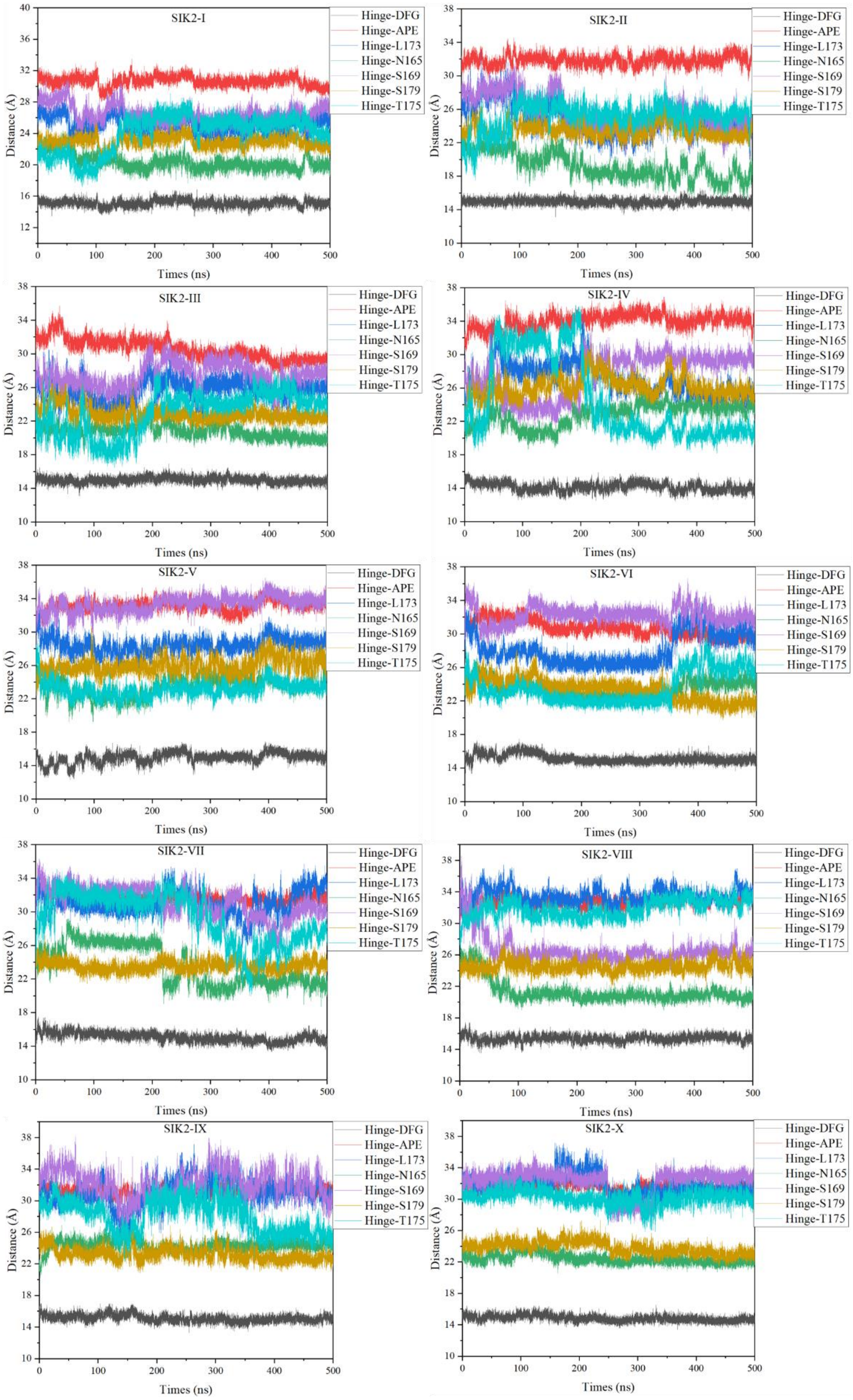

Figure S65. Distance between the T-loop and the hinge loop for bosutinib/SIK2 systems.

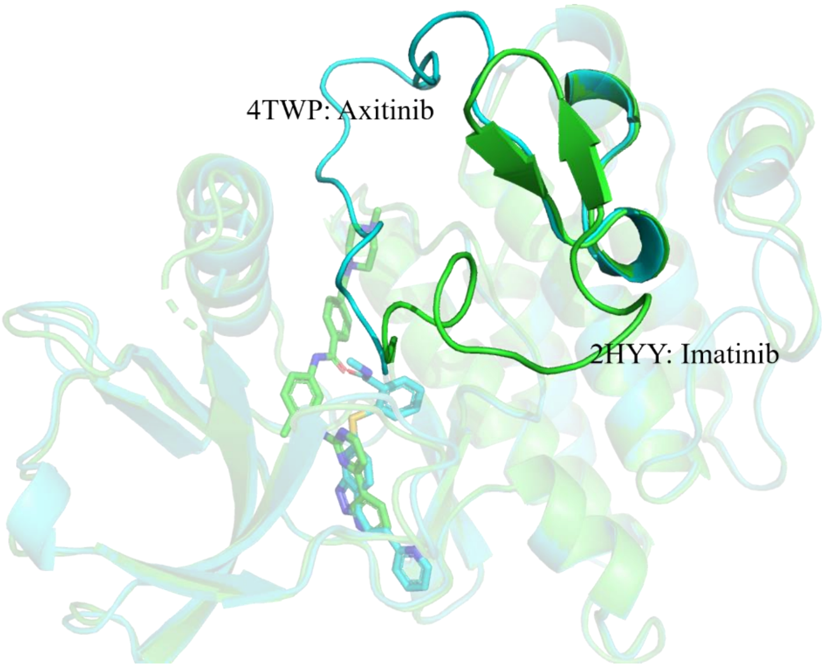

Figure S66. T-loop conformation for ABL1, closed conformation for imatinib (PDB ID: 2HYY) and open conformation for axitinib (PDB ID: 4TWP).

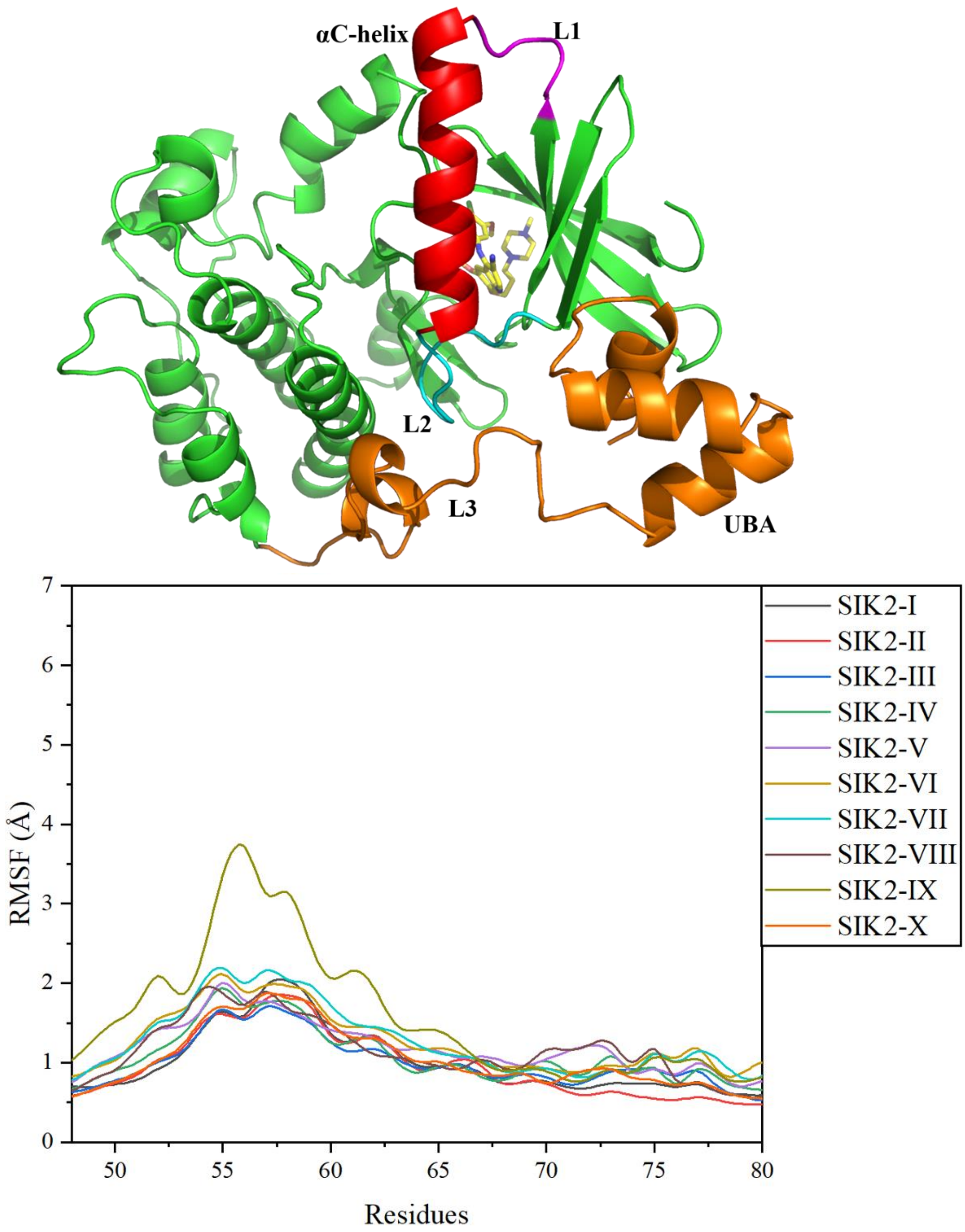

Figure S67. Label for αC-helix of SIK2 in this work and RMSF of the Cα atoms of αC-Helix of SIK2 from simulation trajectory of bosutinib/SIK2 systems with 500 ns MD.

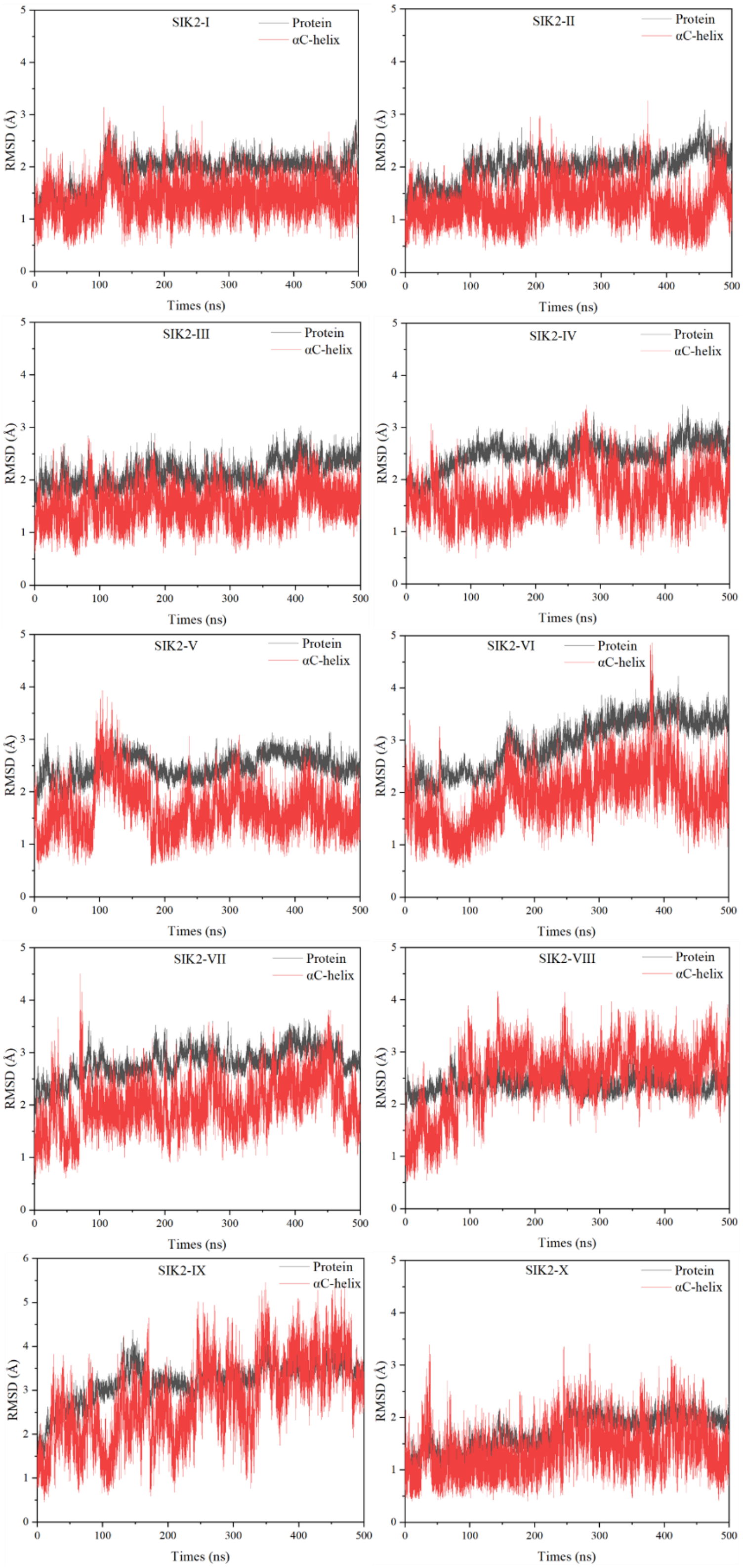

Figure S68. Root mean square deviation (RMSD) value of heavy atoms of backbone of protein and of αC-helix along 500 ns MD simulation for bosutinib/SIK2 systems.

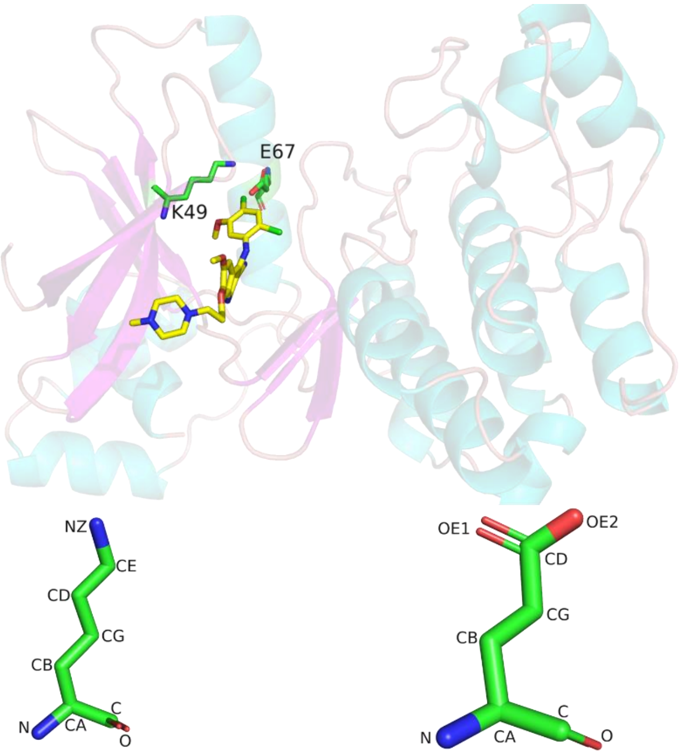

Figure S69. Label for salt bridge between K49 and E67 of SIK2 in this work.

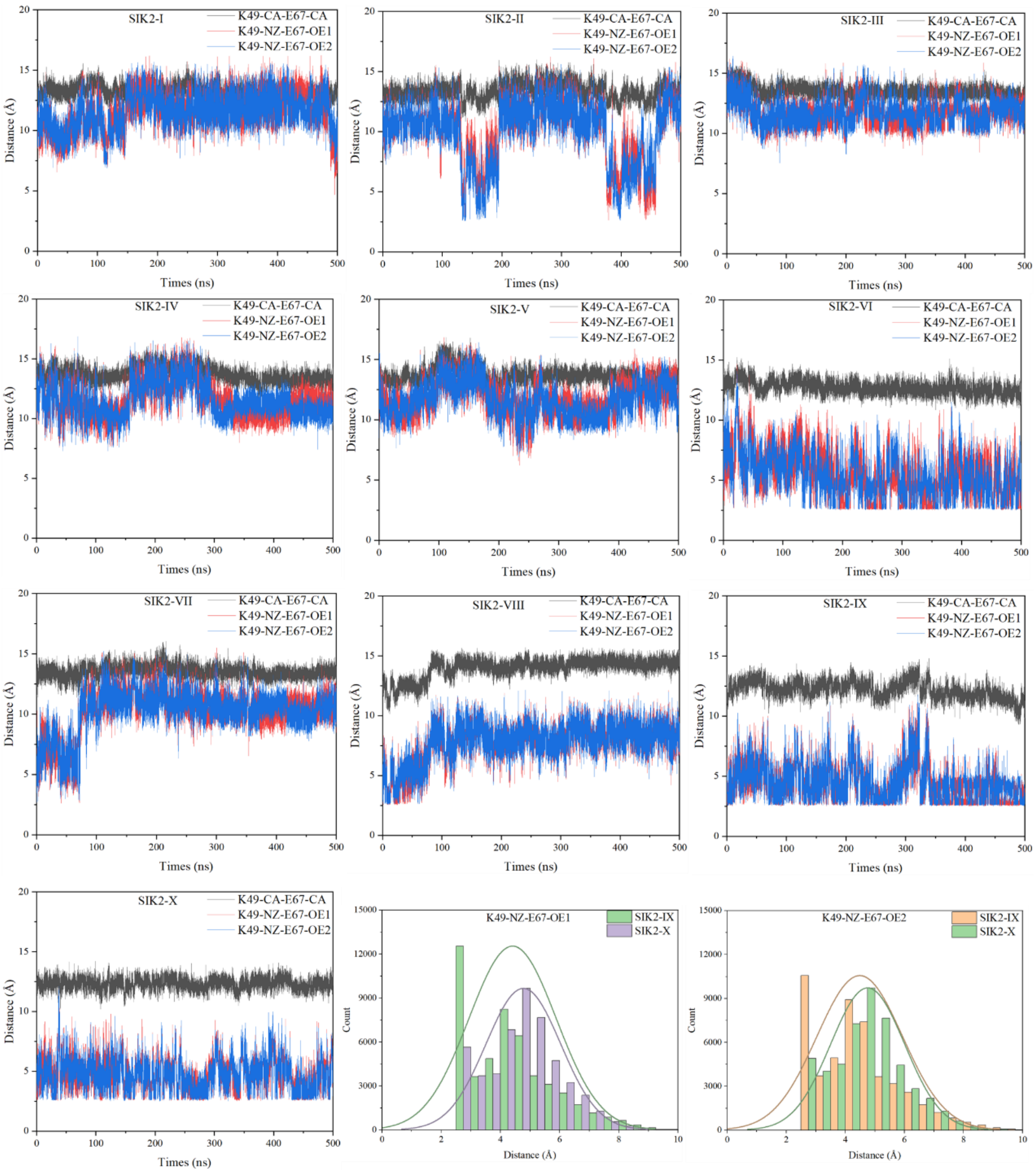

Figure S70. Distance between K49 and E67 for bosutinib/SIK2 systems.

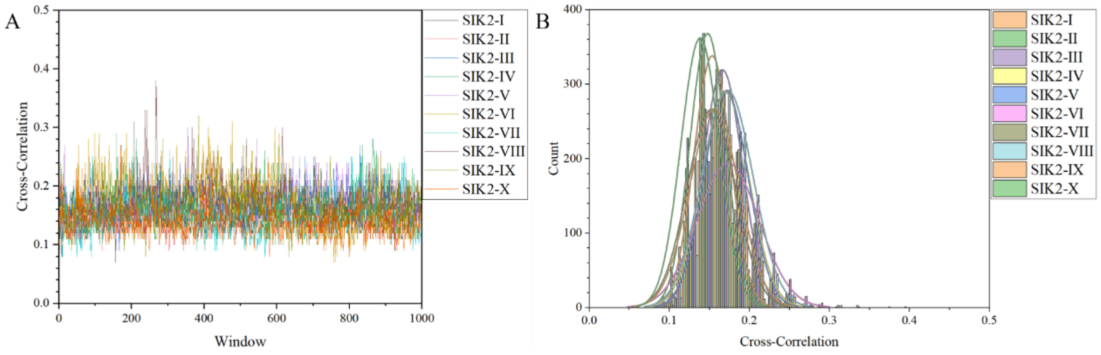

C

SIK2-I	SIK2-II	SIK2-III	SIK2-IV	SIK2-V	SIK2-VI	SIK2-VII	SIK2-VIII	SIK2-IX	SIK2-X
0.15 ± 0.02	0.15 ± 0.02	0.17 ± 0.02	0.17 ± 0.03	0.17 ± 0.03	0.17 ± 0.04	0.15 ± 0.03	0.17 ± 0.03	0.16 ± 0.03	0.14 ± 0.02

Figure S71. Cross-correlation analysis between T-loop and σC-helix for the bosutinib/SIK2 systems.

(A) The cross-correlations between the fluctuations of residues in T-loop (Residues: 160-180) and residues in σC-helix (Residues: 58-73) were calculated although the simulation time with 1 ns every window. (B) The Distribution for the count of cross-correlation for total 998 windows. (C) The cross-correlations between the fluctuations of residues in T-loop (Residues: 160-180) and residues in σC-helix (Residues: 58-73) were calculated in the 500 ns simulation. The detail information for the method to calculate the cross correlation was shown in the **Supporting Information**.

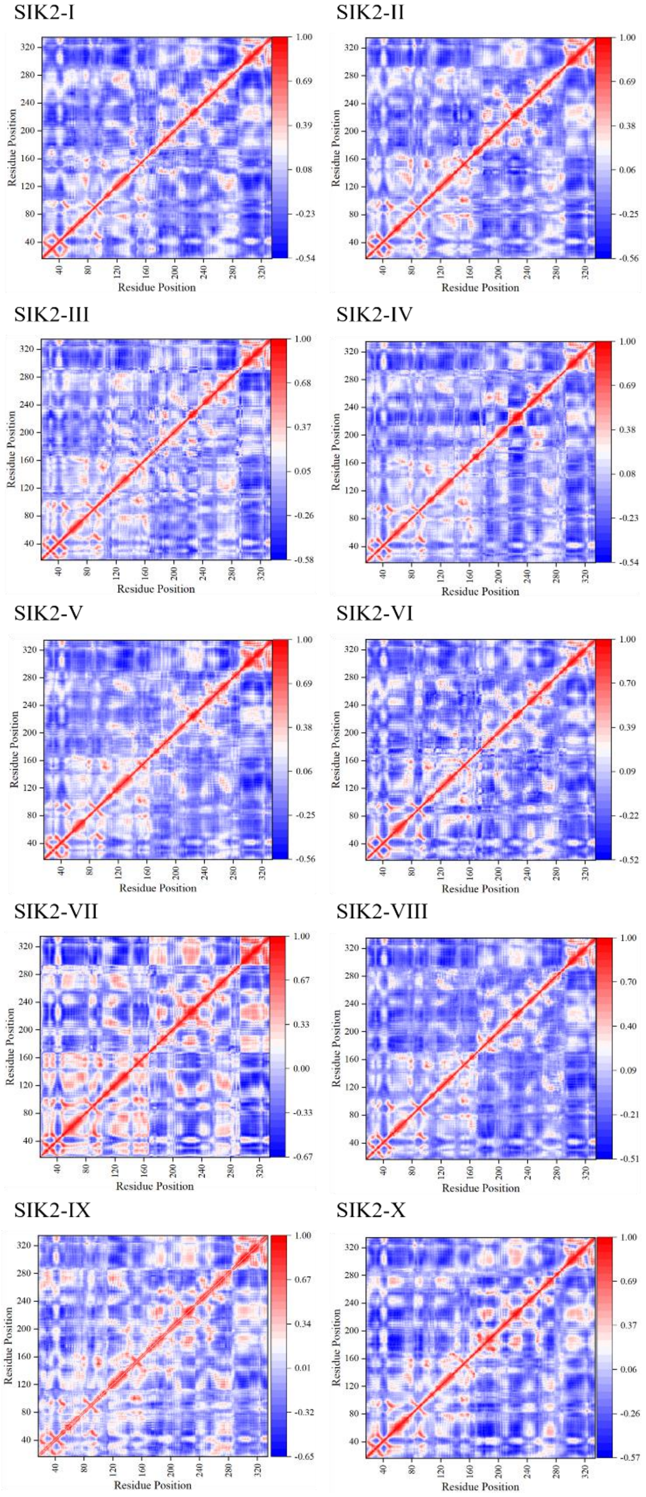

Figure S72. Cross-correlation maps for SIK2 protein of bosutinib/SIK2 complex systems from the last 200 ns of MD simulation for every system.

The red portion is called the "positive region," and it represents pairs of residues moving in the same direction. The blue portion is called the "negative region," which represents pairs of residues moving in the opposite direction.

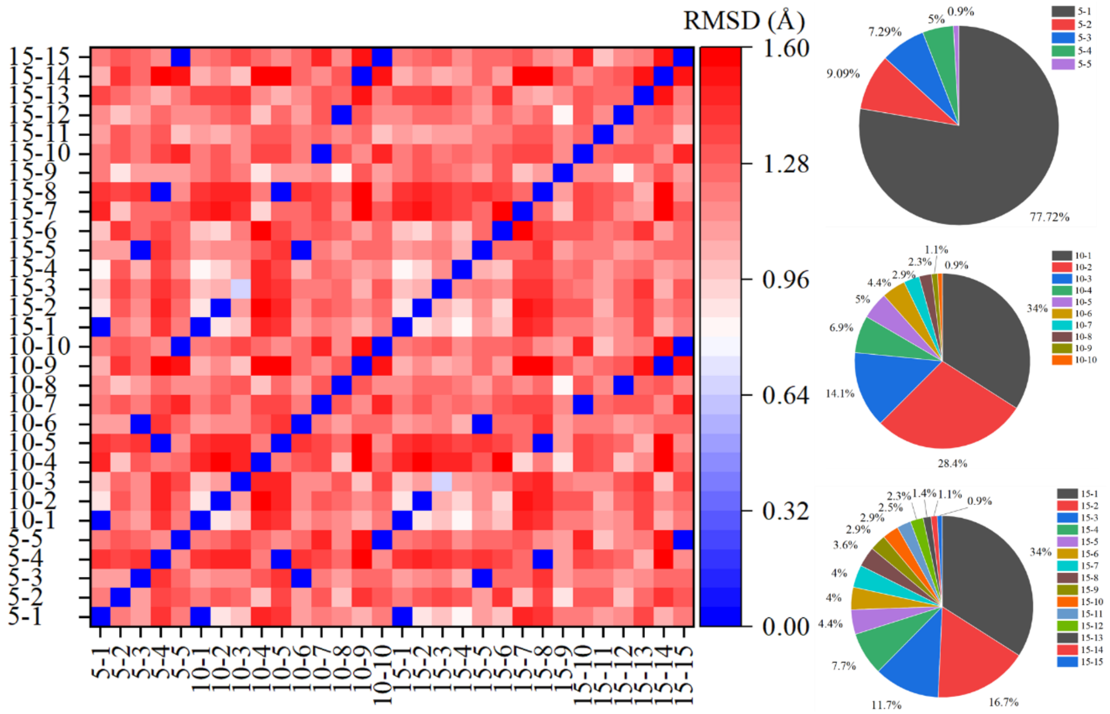

Figure S73. Cluster analysis results for the SIK2-I (bosutinib/SIK2) system.

The frames were defined as the cluster class and cluster number, such as, the 5-1 conformation defined as the representative conformation of the first cluster in the cluster analysis with cluster number 5.

Table S1. Crystal structures for bosutinib binding with protein kinase in the protein database bank.

ID PDB	Macromolecule	DOI
3SOA	Calcium/calmodulin-dependent protein kinase type II subunit alpha with a beta 7 linker(protein)	10.1016/j.cell.2011.07.038
3UE4	Tyrosine-protein kinase ABL1 (protein)	10.1371/journal.pone.0029828
4MXO	Proto-oncogene tyrosine-protein kinase Src (protein)	10.1038/nchembio.1404
4MXX	Proto-oncogene tyrosine-protein kinase Src (protein)	10.1038/nchembio.1404
4MXY	Proto-oncogene tyrosine-protein kinase Src (protein)	10.1038/nchembio.1404
4MXZ	Proto-oncogene tyrosine-protein kinase Src (protein)	10.1038/nchembio.1404
4QMN	Serine/threonine-protein kinase 24 (protein)	10.1002/cmdc.201600115
5AJQ	SERINE/THREONINE-PROTEIN KINASE 10 (protein)	10.2210/pdb5AJQ/pdb
5I9X	Ephrin type-A receptor 2 (protein)	10.1021/acschembio.6b00709
5VC3	Wee1-like protein kinase (protein)	10.1021/acs.jmedchem.7b00996
5VCY	Membrane-associated tyrosine- and threonine-specific cdc2-inhibitory kinase (protein)	10.1021/acs.jmedchem.7b00996
6FDY	Serine/threonine-protein kinase ULK3 (protein)	10.2210/pdb6FDY/pdb
6OP9	Receptor tyrosine-protein kinase erbB-3 (protein)	10.2210/pdb6OP9/pdb

The date for search the structure of bosutinib binding with protein kinase was 2022-02-15 from the Protein Data Bank (PDB) database (https://www.rcsb.org/).

Table S2. Crystal structures for MARK in the protein database bank.

ID PDB	Macromolecule	T-loop Conformation	Domain	DOI
2HAK	MARK1_HUMAN	closed, missing	kinase UBA	10.1074/jbc.M604865200
3OSE	MARK1_HUMAN	NA	KA-1	10.1016/j.cell.2010.11.028
6C9D	MARK1_HUMAN	open	kinase UBA	10.1016/j.str.2018.05.008
3IEC	MARK2_HUMAN	open	kinase UBA	10.1038/nsmb.1705
5EAK	MARK2_HUMAN	closed, missing	kinase UBA	10.1016/j.bmcl.2016.02.003
5KZ7	MARK2_HUMAN	closed, missing	kinase UBA	10.1016/j.bmcl.2016.08.068
5KZ8	MARK2_HUMAN	closed, missing	kinase UBA	10.1016/j.bmcl.2016.08.068
1Y8G	MARK2_RAT	missing	kinase UBA	10.1016/j.str.2005.09.022
1ZMU	MARK2_RAT	missing	kinase UBA	10.1016/j.str.2005.09.022
1ZMV	MARK2_RAT	missing	kinase UBA	10.1016/j.str.2005.09.022
1ZMW	MARK2_RAT	missing	kinase UBA	10.1016/j.str.2005.09.022
2R0I	MARK2_RAT	missing	kinase UBA	10.2210/pdb2R0I/pdb
2WZJ	MARK2_RAT	closed	kinase UBA	10.1096/fj.09-148064
2QNJ	MARK3_HUMAN	closed, missing	kinase UBA	10.1073/pnas.0703012104
3FE3	MARK3_HUMAN	closed	kinase UBA	10.2210/pdb3FE3/pdb
7O94	MARK3_HUMAN	closed, missing	kinase UBA	10.2210/pdb7O94/pdb
1UL7	MARK3_MOUSE	NA	C-terminal	10.1110/ps.062391106
1V5S	MARK3_MOUSE	NA	C-terminal	10.2210/pdb1V5S/pdb
5ES1	MARK4_HUMAN	open, missing	kinase UBA	10.1107/S2053230X15024747

The date for search the structures was 2022-02-15 from the Protein Data Bank (PDB) database (https://www.rcsb.org/).

Table S3. Root mean square deviation (RMSD) value of the overall protein and the T-loop of SIK2 for the ten bosutinib/SIK2 complex systems although 500 ns MD simulations.

RMSD (Å)	Protein	T-loop
bosutinib/**SIK2-I**	1.92 ± 0.32	3.45 ± 0.95
bosutinib/**SIK2-II**	1.99 ± 0.28	4.39 ± 1.07
bosutinib/**SIK2-III**	2.13 ± 0.25	3.30 ± 0.48
bosutinib/**SIK2-IV**	2.48 ± 0.28	4.46 ± 0.76
bosutinib/**SIK2-V**	2.48 ± 0.23	3.85 ± 0.29
bosutinib/**SIK2-VI**	2.94 ± 0.51	3.60 ± 0.35
bosutinib/**SIK2-VII**	2.82 ± 0.28	5.27 ± 0.82
bosutinib/**SIK2-VIII**	2.37 ± 0.16	4.82 ± 0.31
bosutinib/**SIK2-IX**	3.20 ± 0.44	6.42 ± 1.07
bosutinib/**SIK2-X**	1.72 ± 0.33	2.66 ± 1.13

Table S4. Distance value of K49 and E67 of SIK2 for the ten bosutinib/SIK2 complex systems although 500 ns MD simulations.

Distance (Å)	$D_{K49:CA-E67:CA}$	$D_{K49:NZ-E67:OE1}$	$D_{K49:NZ-E67:OE2}$
bosutinib/**SIK2-I**	13.35±0.52	11.62±1.54	11.55±1.41
bosutinib/**SIK2-II**	13.44±0.71	10.03±2.46	9.80±2.68
bosutinib/**SIK2-III**	13.56±0.50	11.75±1.03	11.77±1.01
bosutinib/**SIK2-IV**	13.78±0.66	11.58±1.44	11.55±1.41
bosutinib/**SIK2-V**	13.76±0.69	11.87±1.40	11.65±1.41
bosutinib/**SIK2-VI**	12.73±0.56	5.56±1.88	5.38±1.77
bosutinib/**SIK2-VII**	13.55±0.49	10.25±1.85	10.16±1.77
bosutinib/**SIK2-VIII**	13.97±0.83	7.75±1.62	7.78±1.55
bosutinib/**SIK2-IX**	12.19±0.72	4.41±1.48	4.49±1.45
bosutinib/**SIK2-X**	12.32±0.47	4.77±1.27	4.76±1.25

Table S5. Binding free energies (ΔG_{bind}^{cal}) for bosutinib/SIK2-I complexes.

Energy	Complex		Receptor		Ligand		Delta	
	Average	Std. Dev.	Average	Std. Dev.	Average	Std. Dev.	Average	Std. Dev.
E_{vdW}	-2633.00	25.51	-2581.38	24.86	-7.11	1.89	-44.51	3.86
E_{ele}	-22791.46	126.62	-22954.30	126.35	186.69	1.87	-23.84	4.21
E_{GB}	-3518.42	100.24	-3542.72	100.11	-14.27	0.75	38.57	3.84
E_{surf}	118.39	2.21	119.47	2.17	4.37	0.19	-5.45	0.43
G_{gas}	-6482.40	120.93	-6238.20	119.75	-175.85	6.19	-68.35	6.19
G_{solv}	-3400.03	99.17	-3423.25	99.11	-9.90	0.68	33.12	3.68
$E_{gas} + G_{sol}$	-9882.43	55.30	-9661.45	54.29	-185.75	6.18	-35.23	4.48
TS_{total}	3643.35	9.68	3599.43	10.02	65.77	0.88	-21.85	3.85
ΔG_{bind}^{cal}							-13.38	5.91

The binding free energies ($\mathbf{\Delta G_{bind}^{cal}}$) for bosutinib/SIK2-I complexes and decomposition to electrostatic interaction ($\mathbf{E_{ele}}$), van der Walls interaction ($\mathbf{E_{vdW}}$), solvation free energies ($\mathbf{E_{GB}}$), and entropy ($\mathbf{TS_{total}}$). Energy values are presented in kcal/mol. Uncertainties shown in parentheses were calculated as the root mean square error for all frames extracted from the trajectories. Energy values are in kcal/mol.

Table S6. Binding free energies (ΔG_{bind}^{cal}) for bosutinib/SIK2-II complexes.

Energy(kcal/mol)	Complex		Receptor		Ligand		Delta	
	Average	Std. Dev.	Average	Std. Dev.	Average	Std. Dev.	Average	Std. Dev.
E_{vdW}	-2640.53	24.51	-2583.47	24.37	-4.98	2.07	-52.07	3.61
E_{ele}	-22548.49	124.63	-22723.42	124.88	187.28	2.01	-12.35	3.33
E_{GB}	-3711.35	103.25	-3723.78	103.45	-15.20	0.70	27.62	3.08
E_{surf}	120.21	2.27	122.20	2.30	4.48	0.16	-6.48	0.40
G_{gas}	-6235.09	123.99	-5995.17	124.21	-175.49	5.78	-64.43	4.66
G_{solv}	-3591.14	102.64	-3601.57	102.77	-10.71	0.69	21.14	3.01
$E_{gas} + G_{sol}$	-9826.23	54.43	-9596.74	54.43	-186.20	5.78	-43.29	3.61
TS_{total}	3653.08	8.88	3611.48	9.04	66.18	0.57	-24.58	4.72
ΔG_{bind}^{cal}							-18.70	5.94

The binding free energies ($\mathbf{\Delta G_{bind}^{cal}}$) for bosutinib/SIK2-II complexes and decomposition to electrostatic interaction ($\mathbf{E_{ele}}$), van der Walls interaction ($\mathbf{E_{vdW}}$), solvation free energies ($\mathbf{E_{GB}}$), and entropy ($\mathbf{TS_{total}}$). Energy values are presented in kcal/mol. Uncertainties shown in parentheses were calculated as the root mean square error for all frames extracted from the trajectories. Energy values are in kcal/mol.

Table S7. Binding free energies (ΔG_{bind}^{cal}) for bosutinib/SIK2-III complexes.

Energy(kcal/mol)	Complex		Receptor		Ligand		Delta	
	Average	Std. Dev.	Average	Std. Dev.	Average	Std. Dev.	Average	Std. Dev.
E_{vdW}	-2650.60	30.50	-2587.78	29.55	-5.25	1.90	-57.57	3.19
E_{ele}	-22710.12	119.88	-22886.16	119.71	187.23	1.93	-11.19	3.26
E_{GB}	-3564.12	89.40	-3576.45	89.32	-15.18	0.56	27.51	3.02
E_{surf}	116.78	3.04	119.47	2.97	4.45	0.14	-7.14	0.34
G_{gas}	-6406.03	113.17	-6161.62	112.71	-175.65	5.60	-68.76	4.09
G_{solv}	-3447.34	88.96	-3456.98	88.85	-10.73	0.52	20.37	3.07
$E_{gas} + G_{sol}$	-9853.37	54.74	-9618.61	54.22	-186.37	5.62	-48.39	3.43
TS_{total}	3650.04	10.21	3607.57	9.78	66.14	0.48	-23.67	5.09
ΔG_{bind}^{cal}							-24.72	6.13

The binding free energies ($\mathbf{\Delta G_{bind}^{cal}}$) for bosutinib/SIK2-III complexes and decomposition to electrostatic interaction ($\mathbf{E_{ele}}$), van der Walls interaction ($\mathbf{E_{vdW}}$), solvation free energies ($\mathbf{E_{GB}}$), and entropy ($\mathbf{TS_{total}}$). Energy values are presented in kcal/mol. Uncertainties shown in parentheses were calculated as the root mean square error for all frames extracted from the trajectories. Energy values are in kcal/mol.

Table S8. Binding free energies (ΔG_{bind}^{cal}) for bosutinib/SIK2-IV complexes.

Energy (kcal/mol)	Complex		Receptor		Ligand		Delta	
	Average	Std. Dev.	Average	Std. Dev.	Average	Std. Dev.	Average	Std. Dev.
E_{vdW}	-2623.46	24.94	-2562.05	24.82	-4.14	1.74	-57.27	3.47
E_{ele}	-22543.56	128.71	-22720.46	128.96	187.51	1.96	-10.60	3.50
E_{GB}	-3666.54	101.52	-3678.88	101.46	-15.19	0.65	27.54	3.12
E_{surf}	119.74	2.42	121.98	2.41	4.55	0.14	-6.79	0.31
G_{gas}	-6232.60	124.50	-5989.58	124.24	-175.15	5.60	-67.87	4.87
G_{solv}	-3546.80	100.51	-3556.90	100.50	-10.64	0.62	20.74	3.05
$E_{gas} + G_{sol}$	-9779.40	53.59	-9546.49	53.39	-185.79	5.59	-47.13	3.40
TS_{total}	3655.24	11.53	3612.46	11.80	66.41	0.31	-23.64	6.05
ΔG_{bind}^{cal}							-23.49	6.94

The binding free energies ($\mathbf{\Delta G_{bind}^{cal}}$) for bosutinib/SIK2-IV complexes and decomposition to electrostatic interaction ($\mathbf{E_{ele}}$), van der Walls interaction ($\mathbf{E_{vdW}}$), solvation free energies ($\mathbf{E_{GB}}$), and entropy ($\mathbf{TS_{total}}$). Energy values are presented in kcal/mol. Uncertainties shown in parentheses were calculated as the root mean square error for all frames extracted from the trajectories. Energy values are in kcal/mol.

Table S9. Binding free energies (ΔG_{bind}^{cal}) for bosutinib/SIK2-V complexes.

Energy (kcal/mol)	Complex		Receptor		Ligand		Delta	
	Average	Std. Dev.	Average	Std. Dev.	Average	Std. Dev.	Average	Std. Dev.
E_{vdW}	-2621.88	28.04	-2560.48	27.81	-5.80	3.00	-55.60	4.04
E_{ele}	-22592.23	131.96	-22769.87	131.58	186.66	2.02	-9.01	4.70
E_{GB}	-3700.88	111.70	-3714.51	111.03	-14.31	1.09	27.94	4.02
E_{surf}	120.43	2.57	122.80	2.51	4.41	0.18	-6.78	0.43
G_{gas}	-6223.84	131.90	-5983.04	130.27	-176.18	5.80	-64.62	6.86
G_{solv}	-3580.45	110.89	-3591.71	110.35	-9.90	1.01	21.16	3.85
$E_{gas} + G_{sol}$	-9804.29	54.73	-9574.75	54.19	-186.08	5.76	-43.46	4.75
TS_{total}	3653.30	9.74	3610.63	11.16	65.63	0.92	-22.97	6.93
ΔG_{bind}^{cal}							-20.49	8.40

The binding free energies ($\mathbf{\Delta G_{bind}^{cal}}$) for bosutinib/SIK2-V complexes and decomposition to electrostatic interaction ($\mathbf{E_{ele}}$), van der Walls interaction ($\mathbf{E_{vdW}}$), solvation free energies ($\mathbf{E_{GB}}$), and entropy ($\mathbf{TS_{total}}$). Energy values are presented in kcal/mol. Uncertainties shown in parentheses were calculated as the root mean square error for all frames extracted from the trajectories. Energy values are in kcal/mol.

Table S10. Binding free energies (ΔG_{bind}^{cal}) for bosutinib/SIK2-VI complexes.

Energy (kcal/mol)	Complex		Receptor		Ligand		Delta	
	Average	Std. Dev.	Average	Std. Dev.	Average	Std. Dev.	Average	Std. Dev.
E_{vdW}	-2624.96	26.85	-2573.51	25.45	-6.21	2.09	-45.24	4.44
E_{ele}	-22691.71	118.75	-22866.57	119.05	185.50	1.80	-10.64	3.91
E_{GB}	-3565.16	98.24	-3576.39	98.31	-14.13	0.60	25.37	3.77
E_{surf}	118.90	2.14	120.30	2.04	4.48	0.15	-5.88	0.52
G_{gas}	-6352.67	124.84	-6120.23	124.72	-176.55	5.59	-55.88	7.19
G_{solv}	-3446.26	97.59	-3456.09	97.64	-9.66	0.52	19.49	3.45
$E_{gas} + G_{sol}$	-9798.93	56.53	-9576.32	56.57	-186.21	5.56	-36.39	4.77
TS_{total}	3653.27	9.77	3610.59	9.51	66.27	0.31	-23.59	5.04
ΔG_{bind}^{cal}							-12.81	6.94

The binding free energies ($\mathbf{\Delta G_{bind}^{cal}}$) for bosutinib/SIK2-VI complexes and decomposition to electrostatic interaction ($\mathbf{E_{ele}}$), van der Walls interaction ($\mathbf{E_{vdW}}$), solvation free energies ($\mathbf{E_{GB}}$), and entropy ($\mathbf{TS_{total}}$). Energy values are presented in kcal/mol. Uncertainties shown in parentheses were calculated as the root mean square error for all frames extracted from the trajectories. Energy values are in kcal/mol.

Table S11. Binding free energies (ΔG_{bind}^{cal}) for bosutinib/SIK2-VII complexes.

Energy (kcal/mol)	Complex		Receptor		Ligand		Delta	
	Average	Std. Dev.	Average	Std. Dev.	Average	Std. Dev.	Average	Std. Dev.
E_{vdW}	-2620.68	26.97	-2558.71	27.85	-4.76	1.81	-57.20	3.99
E_{ele}	-22575.95	130.39	-22751.91	131.22	186.98	2.04	-11.02	3.67
E_{GB}	-3661.07	105.85	-3673.64	106.41	-15.26	0.69	27.83	3.41
E_{surf}	119.38	2.48	121.87	2.55	4.52	0.11	-7.02	0.46
G_{gas}	-6236.64	126.99	-5992.73	126.85	-175.69	5.77	-68.22	5.01
G_{solv}	-3541.70	104.77	-3551.77	105.47	-10.74	0.66	20.81	3.38
$E_{gas} + G_{sol}$	-9778.33	55.38	-9544.49	55.50	-186.42	5.76	-47.41	3.93
TS_{total}	3663.03	9.76	3622.58	9.77	66.33	0.25	-25.87	5.27
ΔG_{bind}^{cal}							-21.54	6.58

The binding free energies ($\mathbf{\Delta G_{bind}^{cal}}$) for bosutinib/SIK2-VII complexes and decomposition to electrostatic interaction ($\mathbf{E_{ele}}$), van der Walls interaction ($\mathbf{E_{vdW}}$), solvation free energies ($\mathbf{E_{GB}}$), and entropy ($\mathbf{TS_{total}}$). Energy values are presented in kcal/mol. Uncertainties shown in parentheses were calculated as the root mean square error for all frames extracted from the trajectories. Energy values are in kcal/mol.

Table S12. Binding free energies (ΔG_{bind}^{cal}) for bosutinib/SIK2-VIII complexes.

Energy (kcal/mol)	Complex		Receptor		Ligand		Delta	
	Average	Std. Dev.	Average	Std. Dev.	Average	Std. Dev.	Average	Std. Dev.
E_{vdW}	-2633.12	25.52	-2576.32	25.60	-4.39	1.64	-52.41	3.03
E_{ele}	-22715.10	134.02	-22893.62	134.09	187.62	1.85	-9.09	3.17
E_{GB}	-3572.17	111.98	-3581.96	111.92	-15.52	0.57	25.31	3.06
E_{surf}	117.28	2.27	119.11	2.28	4.57	0.10	-6.40	0.31
G_{gas}	-6356.36	131.72	-6119.92	130.86	-174.93	5.89	-61.51	4.36
G_{solv}	-3454.90	111.11	-3462.85	111.05	-10.95	0.55	18.90	2.94
$E_{gas} + G_{sol}$	-9811.26	53.05	-9582.77	52.46	-185.88	5.89	-42.60	2.89
TS_{total}	3656.70	9.16	3614.33	9.48	66.47	0.15	-24.10	4.42
ΔG_{bind}^{cal}							-18.50	5.28

The binding free energies ($\mathbf{\Delta G_{bind}^{cal}}$) for bosutinib/SIK2-VIII complexes and decomposition to electrostatic interaction ($\mathbf{E_{ele}}$), van der Walls interaction ($\mathbf{E_{vdW}}$), solvation free energies ($\mathbf{E_{GB}}$), and entropy ($\mathbf{TS_{total}}$). Energy values are presented in kcal/mol. Uncertainties shown in parentheses were calculated as the root mean square error for all frames extracted from the trajectories. Energy values are in kcal/mol.

Table S13. Binding free energies (ΔG_{bind}^{cal}) for bosutinib/SIK2-IX complexes.

Energy (kcal/mol)	Complex		Receptor		Ligand		Delta	
	Average	Std. Dev.	Average	Std. Dev.	Average	Std. Dev.	Average	Std. Dev.
E_{vdW}	-2612.76	24.20	-2553.01	24.02	-4.26	1.69	-55.48	3.44
E_{ele}	-22751.43	133.66	-22925.57	133.59	186.40	1.93	-12.26	3.08
E_{GB}	-3496.16	109.52	-3511.29	109.12	-14.83	0.74	29.96	3.19
E_{surf}	120.16	2.34	122.38	2.43	4.59	0.08	-6.81	0.42
G_{gas}	-6397.64	130.18	-6153.25	130.28	-176.63	5.52	-67.76	4.58
G_{solv}	-3376.00	108.65	-3388.91	108.20	-10.24	0.71	23.15	3.05
$E_{gas} + G_{sol}$	-9773.64	55.00	-9542.16	54.91	-186.87	5.53	-44.61	3.48
TS_{total}	3656.86	9.72	3614.95	10.59	66.45	0.15	-24.54	6.12
ΔG_{bind}^{cal}							-20.07	7.04

The binding free energies ($\mathbf{\Delta G_{bind}^{cal}}$) for bosutinib/SIK2-IX complexes and decomposition to electrostatic interaction ($\mathbf{E_{ele}}$), van der Walls interaction ($\mathbf{E_{vdW}}$), solvation free energies ($\mathbf{E_{GB}}$), and entropy ($\mathbf{TS_{total}}$). Energy values are presented in kcal/mol. Uncertainties shown in parentheses were calculated as the root mean square error for all frames extracted from the trajectories. Energy values are in kcal/mol.

Table S14. Binding free energies (ΔG_{bind}^{cal}) for bosutinib/SIK2-X complexes.

Energy (kcal/mol)	Complex		Receptor		Ligand		Delta	
	Average	Std. Dev.	Average	Std. Dev.	Average	Std. Dev.	Average	Std. Dev.
E_{vdW}	-2679.60	27.12	-2621.49	26.94	-4.17	1.78	-53.94	3.62
E_{ele}	-22810.56	123.14	-22987.09	122.54	186.64	2.02	-10.11	3.41
E_{GB}	-3489.91	102.47	-3502.12	101.82	-14.93	0.89	27.13	3.60
E_{surf}	113.65	2.34	115.96	2.31	4.54	0.11	-6.86	0.38
G_{gas}	-6497.79	127.78	-6258.98	127.42	-174.75	5.57	-64.06	5.15
G_{solv}	-3376.26	101.59	-3386.16	100.94	-10.38	0.85	20.27	3.44
$E_{gas} + G_{sol}$	-9874.06	57.01	-9645.14	57.30	-185.14	5.63	-43.78	3.49
TS_{total}	3650.63	10.81	3608.43	11.59	66.40	0.22	-24.20	5.56
ΔG_{bind}^{cal}							-19.58	6.57

The binding free energies ($\mathbf{\Delta G_{bind}^{cal}}$) for bosutinib/SIK2-X complexes and decomposition to electrostatic interaction ($\mathbf{E_{ele}}$), van der Walls interaction ($\mathbf{E_{vdW}}$), solvation free energies ($\mathbf{E_{GB}}$), and entropy ($\mathbf{TS_{total}}$). Energy values are presented in kcal/mol. Uncertainties shown in parentheses were calculated as the root mean square error for all frames extracted from the trajectories. Energy values are in kcal/mol.
